# Supplementary material for: Synthesis of Bis(amino acids) Containing the Styryl-cyclobutane Core by Photosensitized [2+2]-Cross-cycloaddition of Allylidene-5(4H)-oxazolones
Source: Int J Mol Sci. 2023 Apr 20;24(8):7583. doi: 10.3390/ijms24087583 (PMC10140832; doi:10.3390/ijms24087583)
Supplement: Supplementary file 1 [file ijms-24-07583-s001.zip › Supplementary Material_revised.pdf]

Supplementary Material

Synthesis of bis(amino acids) containing the styryl-cyclobutane core by photosensitized [2+2]-cross-cycloaddition of allylidene-5(4*H*)-oxazolones

Sonia Sierra [1], David Dalmau [1], Juan V. Alegre-Requena [1], Alexandra Pop [2], Cristian Silvestru [2], Maria Luisa Marín [3], Francisco Boscá [3] and Esteban P. Urriolabeitia [1,*]

[1] Instituto de Síntesis Química y Catálisis Homogénea (ISQCH; CSIC - Universidad de Zaragoza), Pedro Cerbuna 12, 50009 Zaragoza (Spain); ssierrasainzaja@gmail.com (S. S.); ddalmau@unizar.es (D. D.); jvalegre@unizar.es (J. V. A. R.)

[2] Department of Chemistry, Supramolecular Organic and Organometallic Chemistry Centre (SOOMCC), Faculty of Chemistry and Chemical Engineering, Babeş- Bolyai University, 400028 Cluj-Napoca, Romania; alexandra.m.pop@ubbcluj.ro (A. P.); cristian.silvestru@ubbcluj.ro (C. S.)

[3] Instituto Universitario Mixto de Tecnología Química (ITQ-UPV), Universitat Politècnica de València-CSIC, 46022 Valencia, Spain; marmarin@qim.upv.es (M. L. M.); fbosca@itq.upv.es (F. B.)

*****Correspondence author: esteban@unizar.es

Contents

1.- NMR spectra of all prepared compounds

1.1.- NMR spectra of (*Z,E*)-4-((*E*)-3-aryl-allylidene)-2-phenyl-5(4*H*)-oxazolones **1a-1h**.

1.2.- NMR spectra of the cyclobutane-bis(oxazolone) intermediates **2a-2e**.

1.3.- NMR spectra of the 1,2-diaminotruxinic acid bis(amino acids) **3a-3b**.

2.- Absorption spectra of oxazolones 1a, 1b, 1c and **1f**

3.- Transient Absorption Spectra

Table S1. Half-life values at 470 nm and ruthenium deactivation rate constant at 660 nm

4.- Cyclic Voltammetry of oxazolone 1a

5.- X-ray crystallographic data of 1a

Table S2. Crystal data and structure refinement for **1a**.

Table S3. Atomic coordinates (x10^4) and equivalent isotropic displacement parameters (Å^{2}x 10^3) for **1a**.

Table S4. Bond lengths [Å] and angles [°] for **1a**.

Table S5. Anisotropic displacement parameters (Å^{2}x 10^3) for **1a**.

Table S6. Torsion angles [°] for **1a**.

6.- Computational Details

6.1.- Computational methods

6.2.- Thermochemical data calculation with *GoodVibes*

6.3.- Automation with *AQME*

6.4.- MD simulations

6.5.- T_1 rotation of **4**

1.- NMR spectra of all prepared compounds

1.1.- NMR spectra of (*Z,E*)-4-((*E*)-3-aryl-allylidene)-2-phenyl-5(4*H*)-oxazolones 1a-1h.

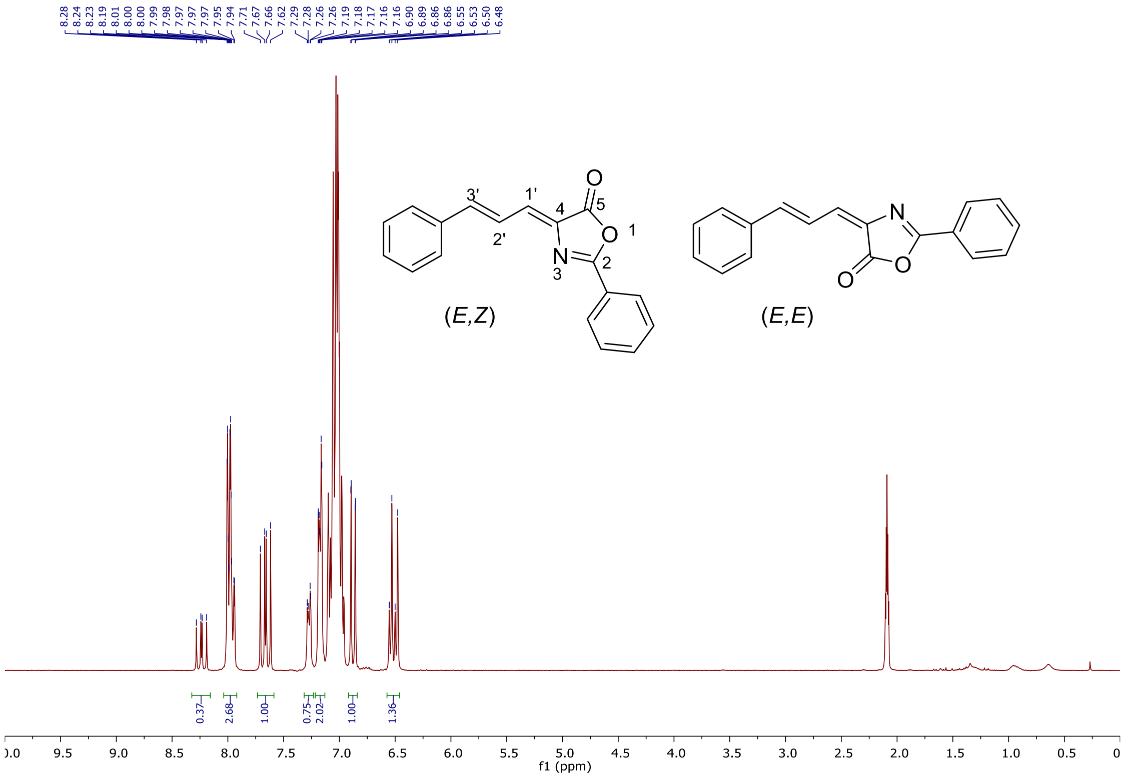

Figure S1. ^{1}H NMR (Toluene-d8, 500.13 MHz) of **1a**

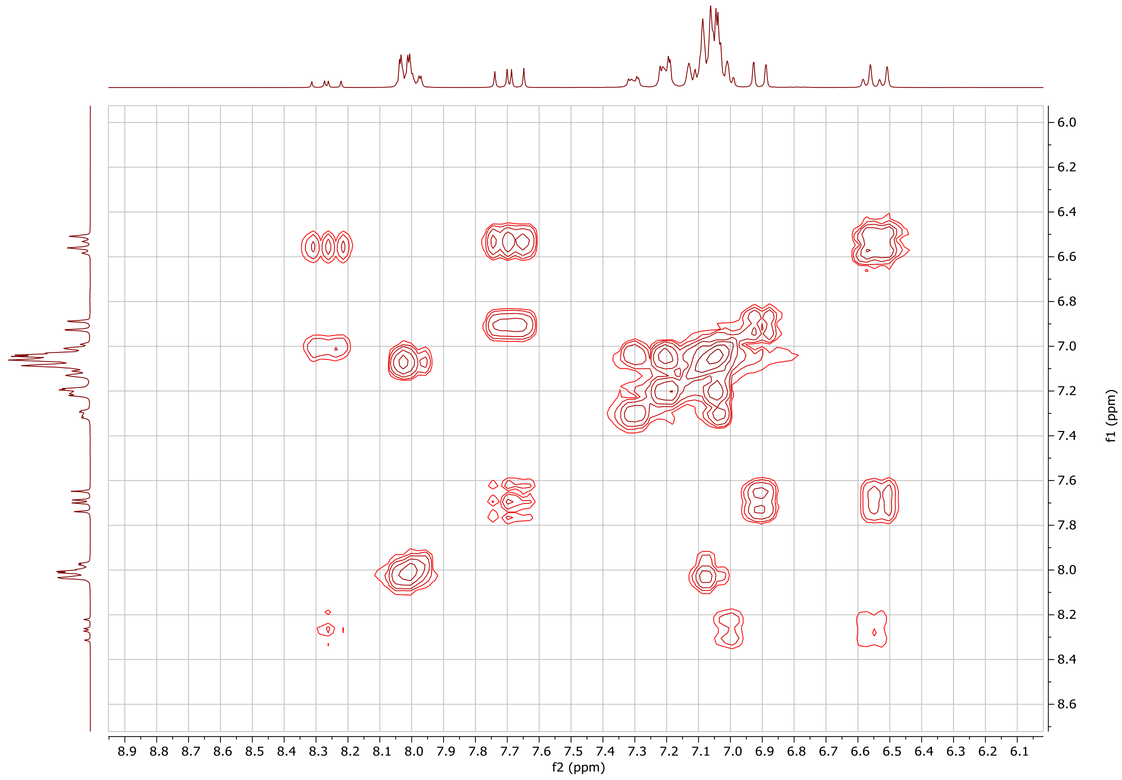

Figure S2. ^{1}H-COSY (Toluene-d8) of **1a**

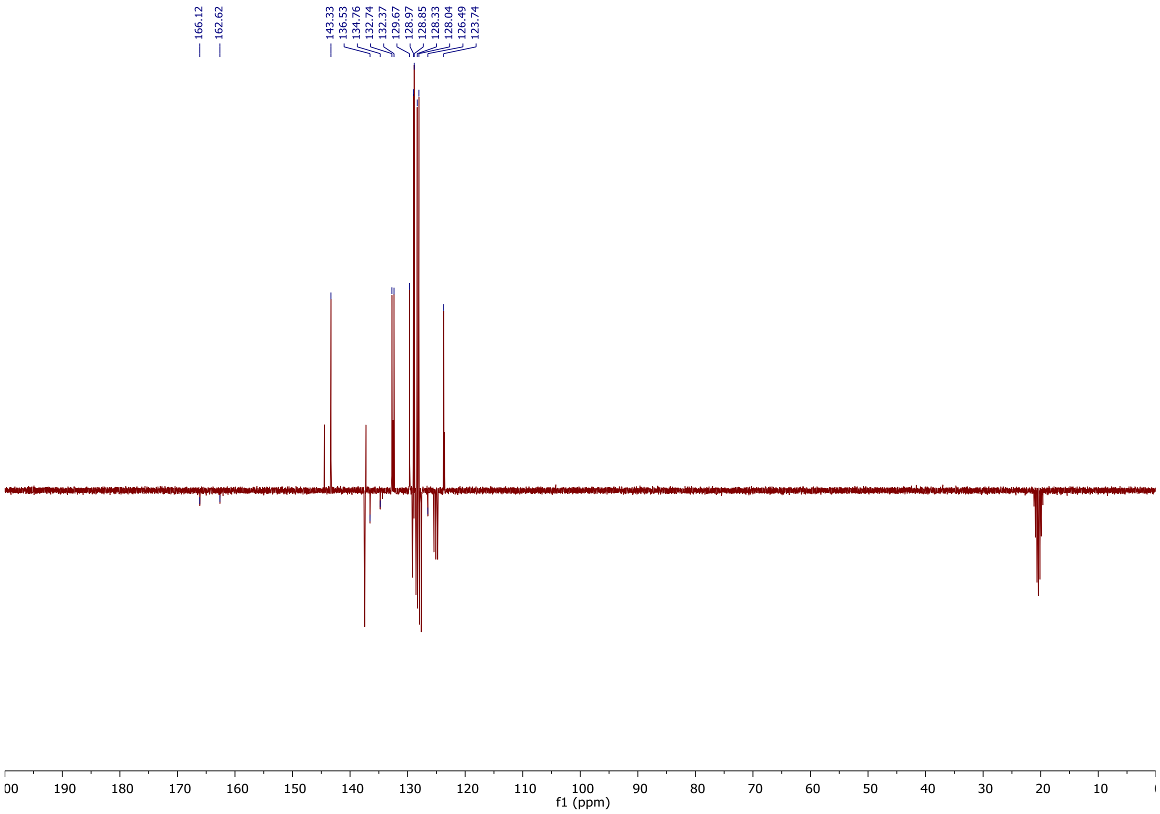

Figure S3. $^{13}C\{^{1}H\}$ (APT) NMR (Toluene-d8, 125.7 MHz) of **1a**

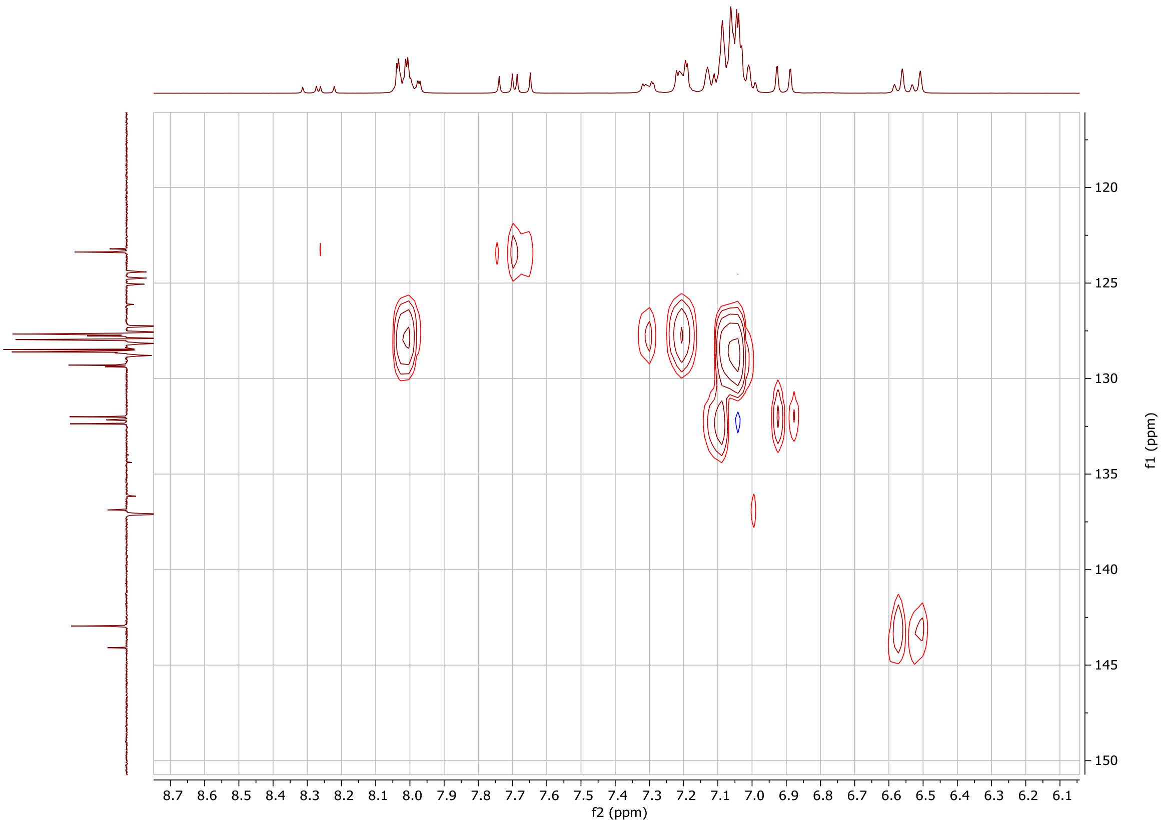

Figure S4. ^{1}H-^{13}C HSQC correlation (Toluene-d8) of **1a**

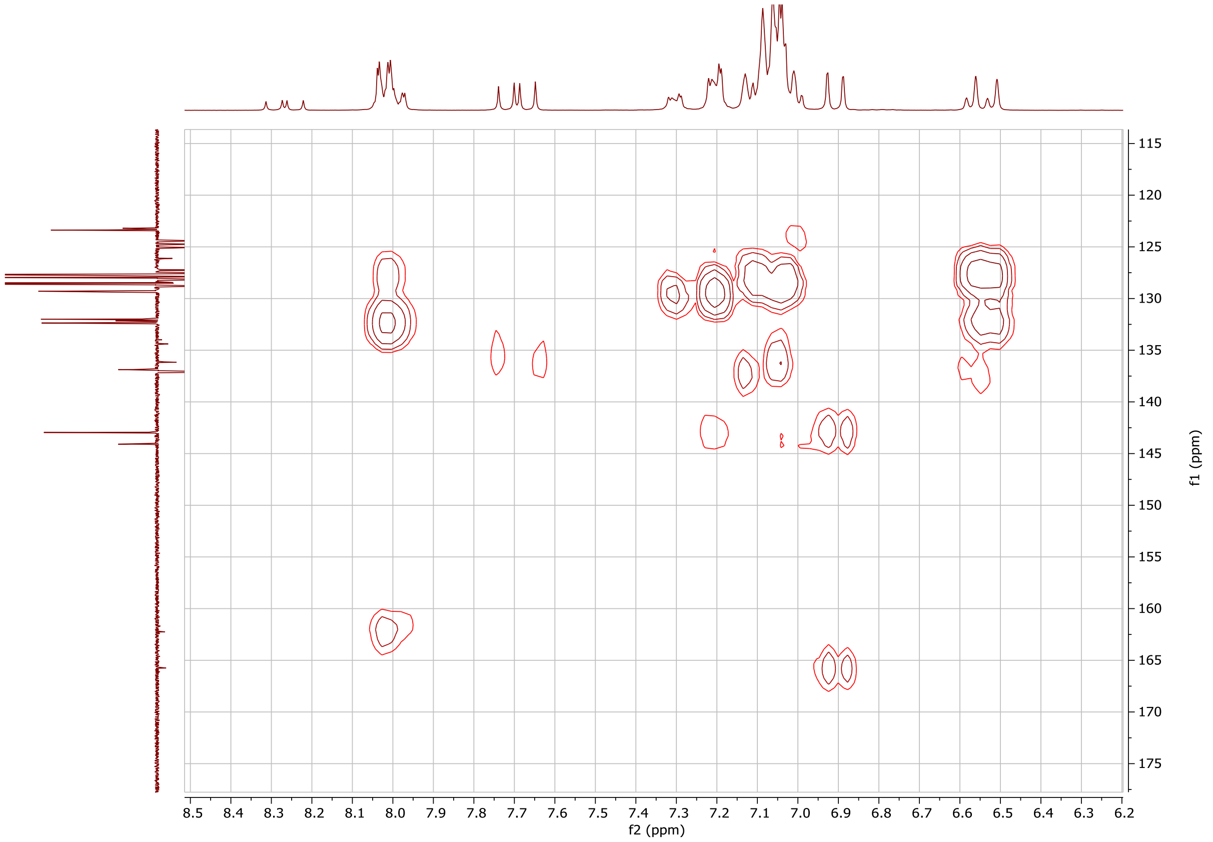

Figure S5. ^{1}H-^{13}C HMBC correlation (Toluene-d8) of **1a**

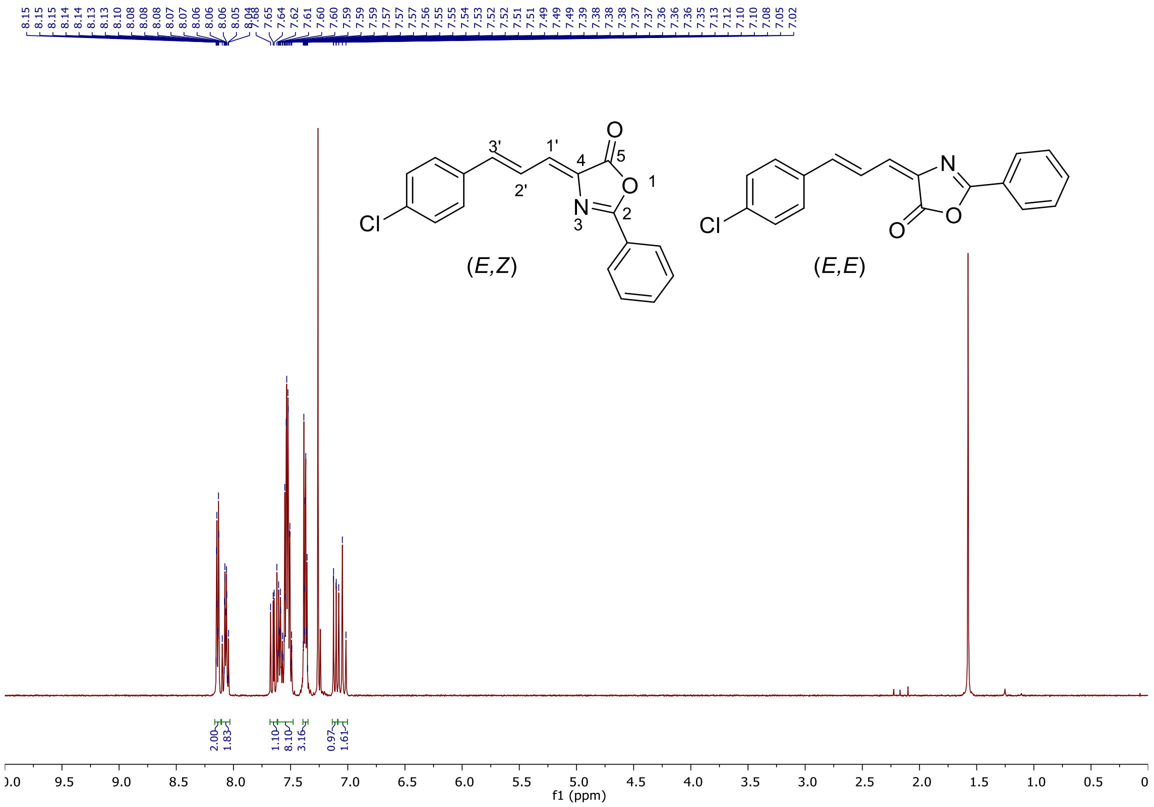

Figure S6. ^{1}H NMR ($CDCl_3$, 500.13 MHz) of **1b**

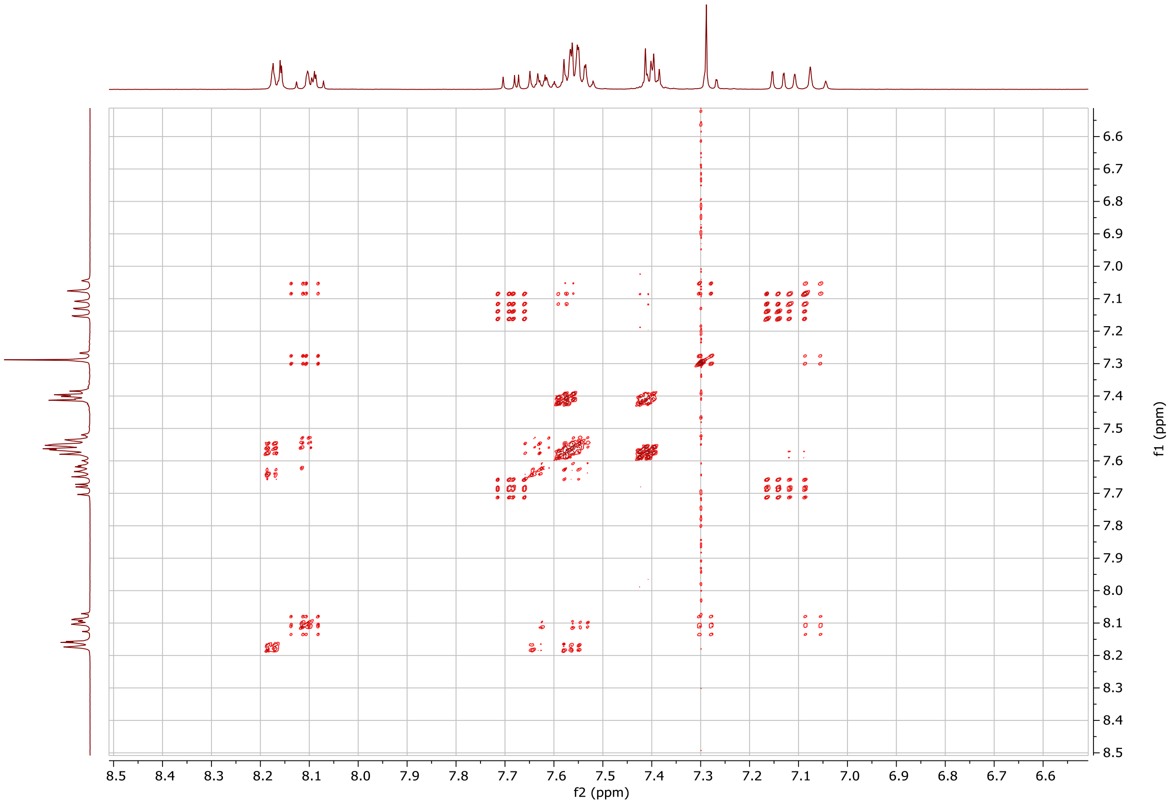

Figure S7. ^{1}H-COSY ($CDCl_3$) of **1b**

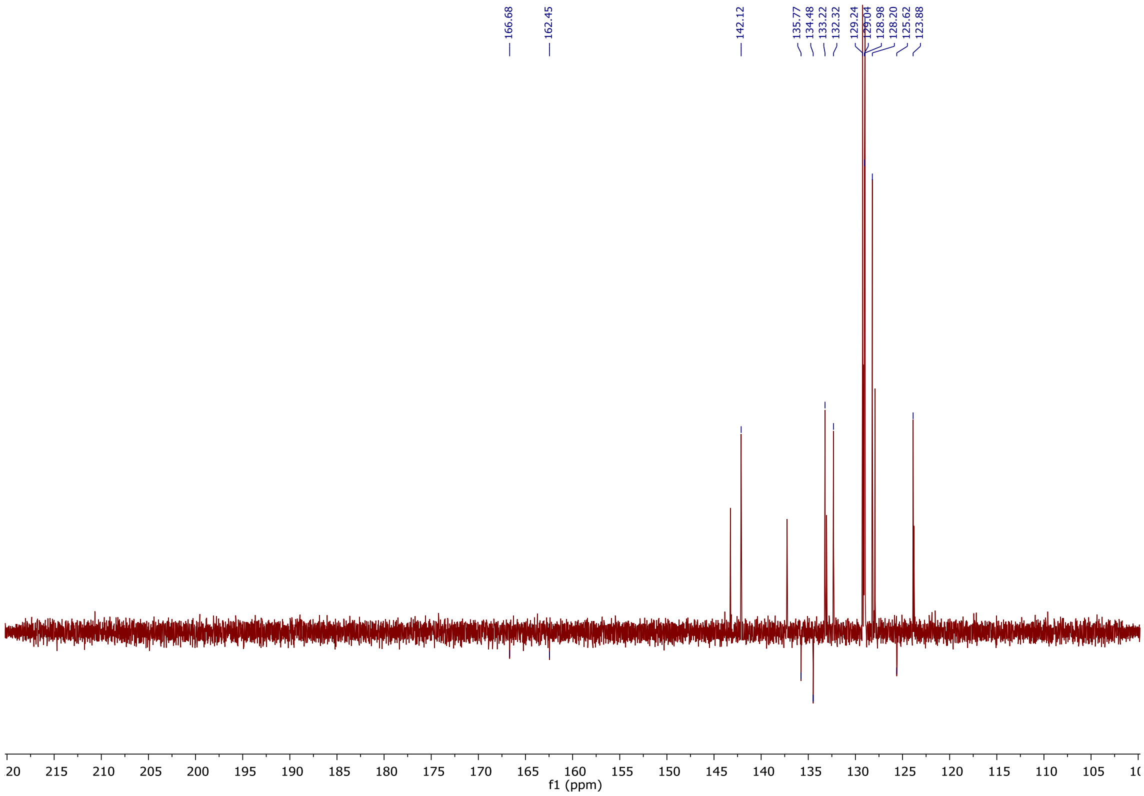

Figure S8. ^{13}C {^{1}H} (APT) NMR ($CDCl_3$, 125.7 MHz) of **1b**

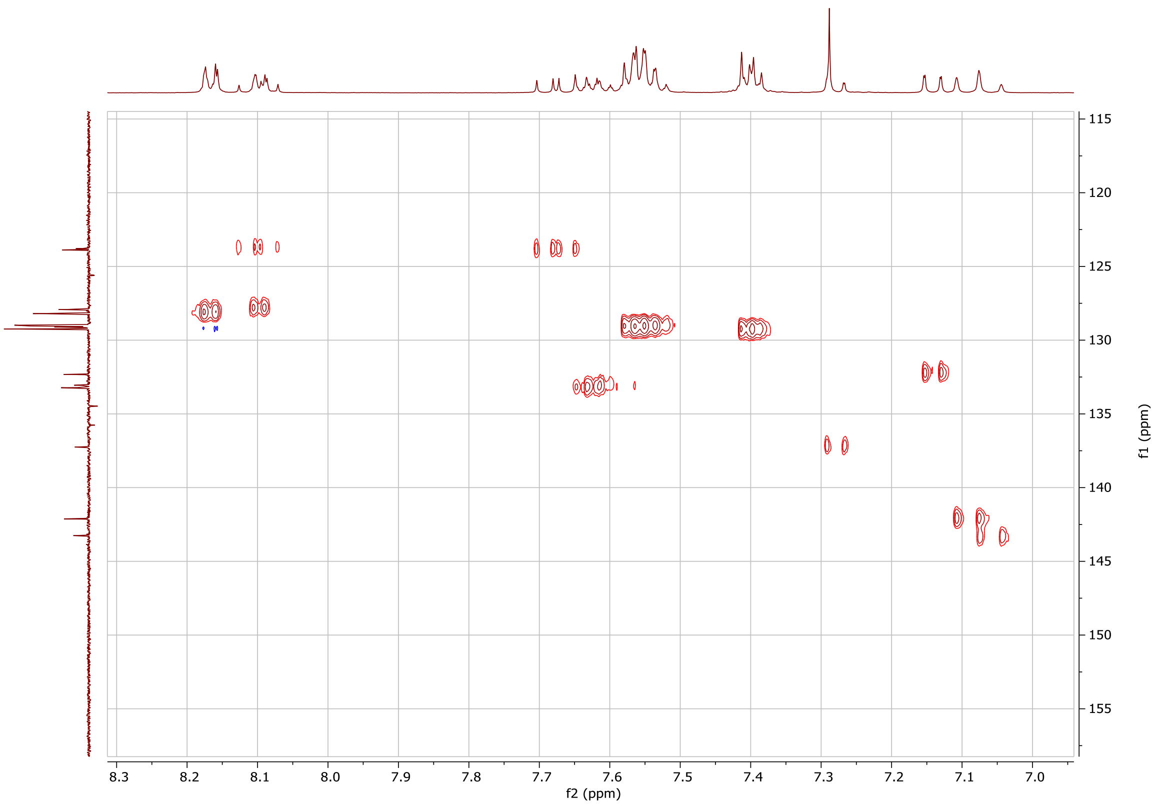

Figure S9. ^{1}H-^{13}C HSQC correlation ($CDCl_3$) of **1b**

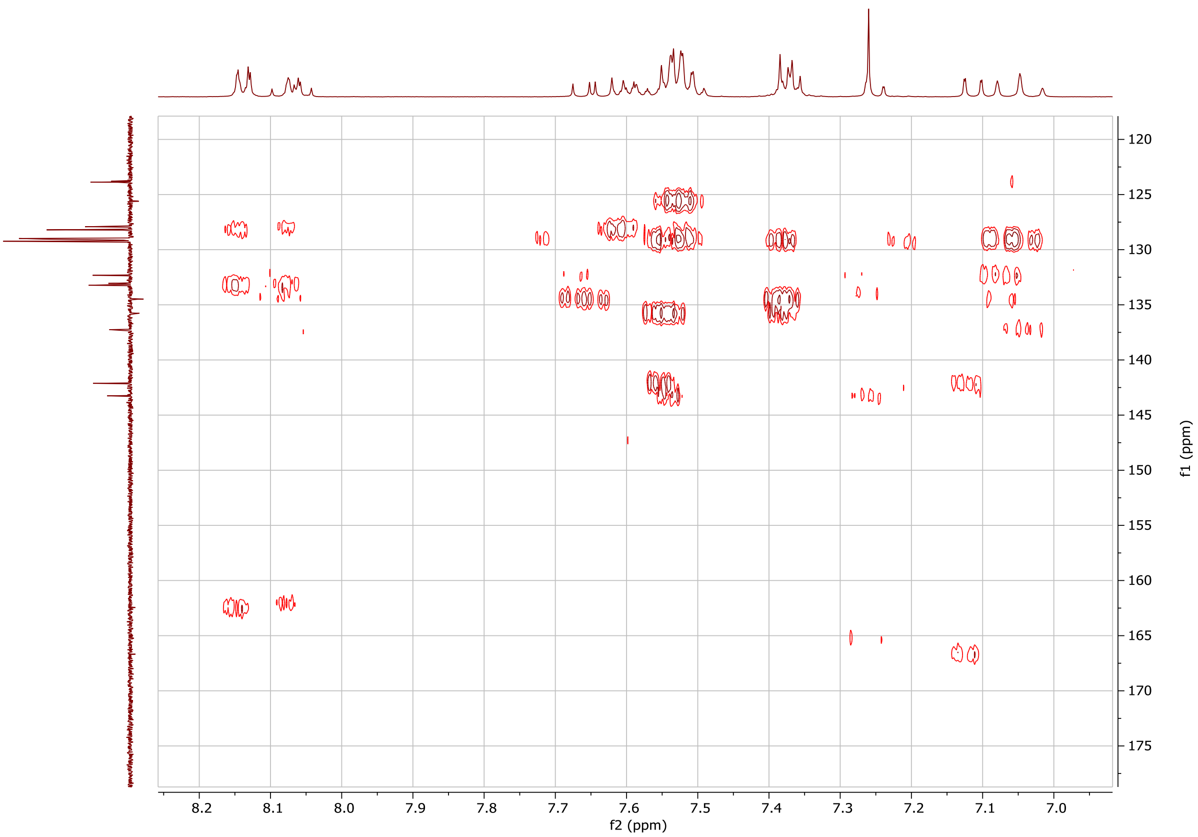

Figure S10. ^{1}H-^{13}C HMBC correlation ($CDCl_3$) of **1b**

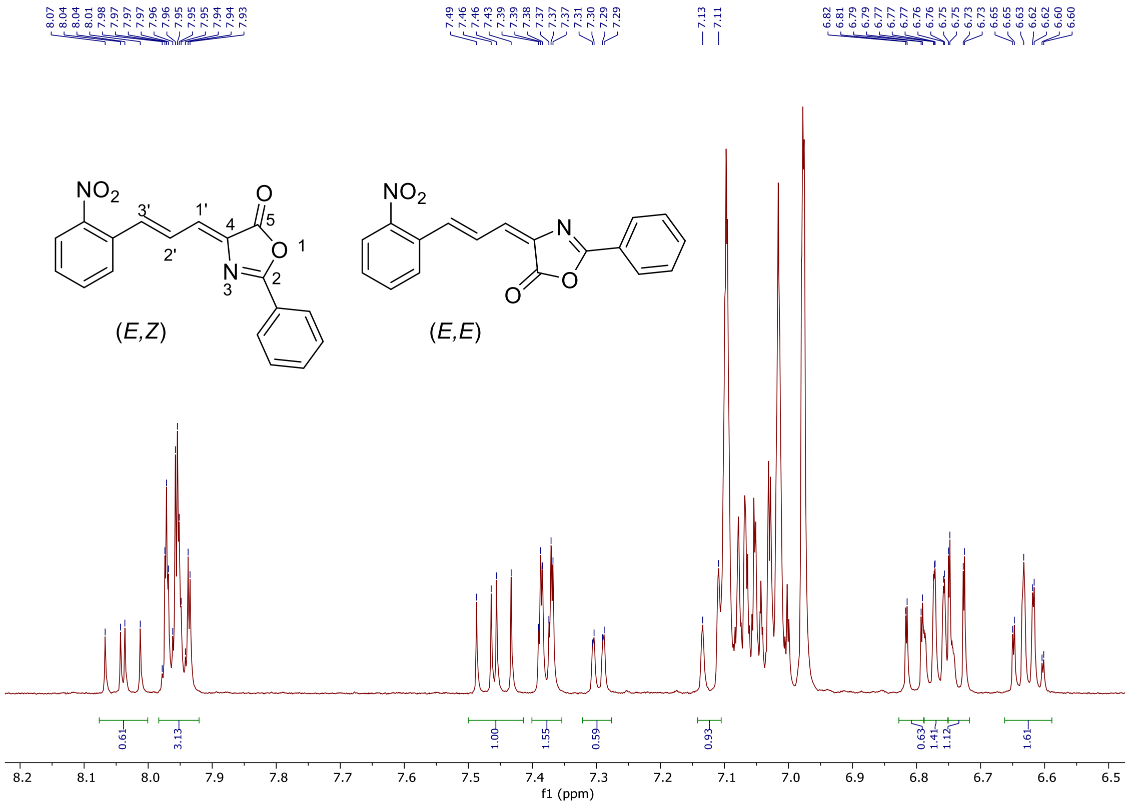

Figure S11. ^{1}H NMR (Toluene-d8, 500.13 MHz) of **1c**

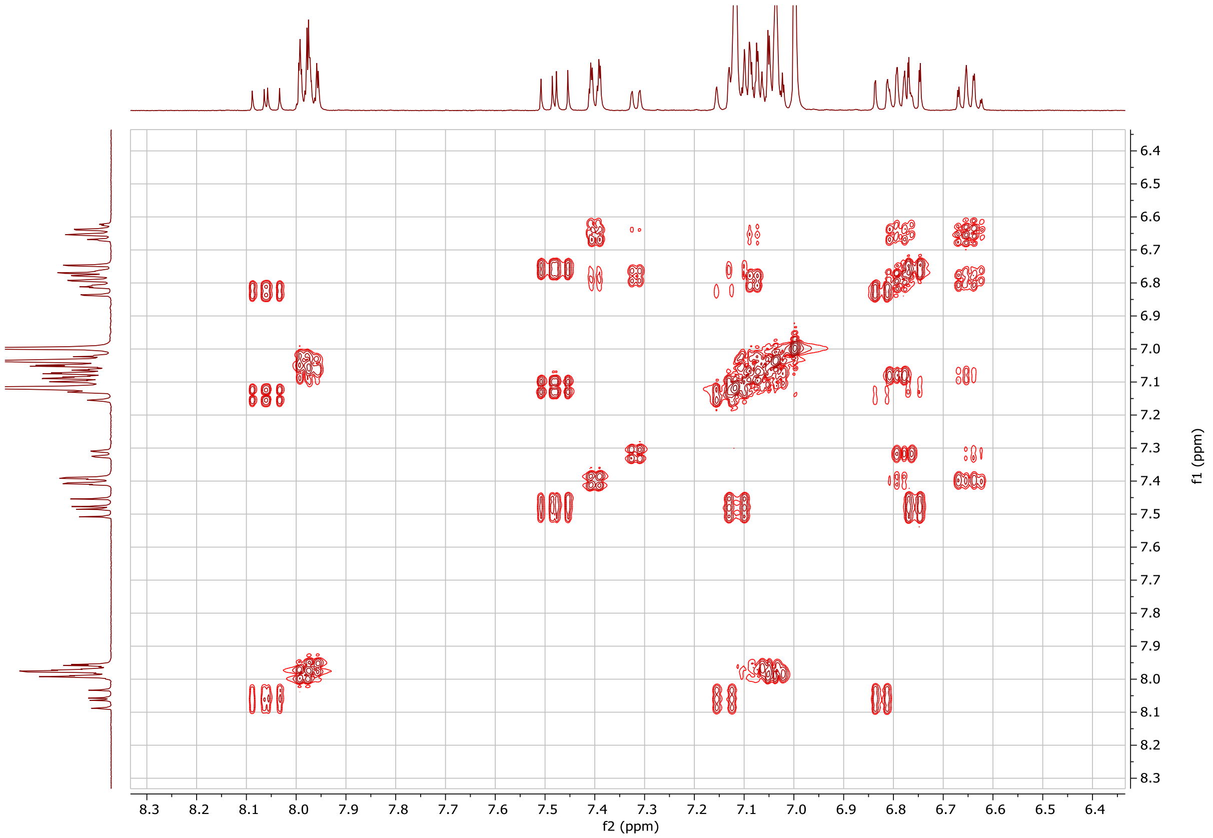

Figure S12. ^{1}H-COSY (Toluene-d8) of **1c**

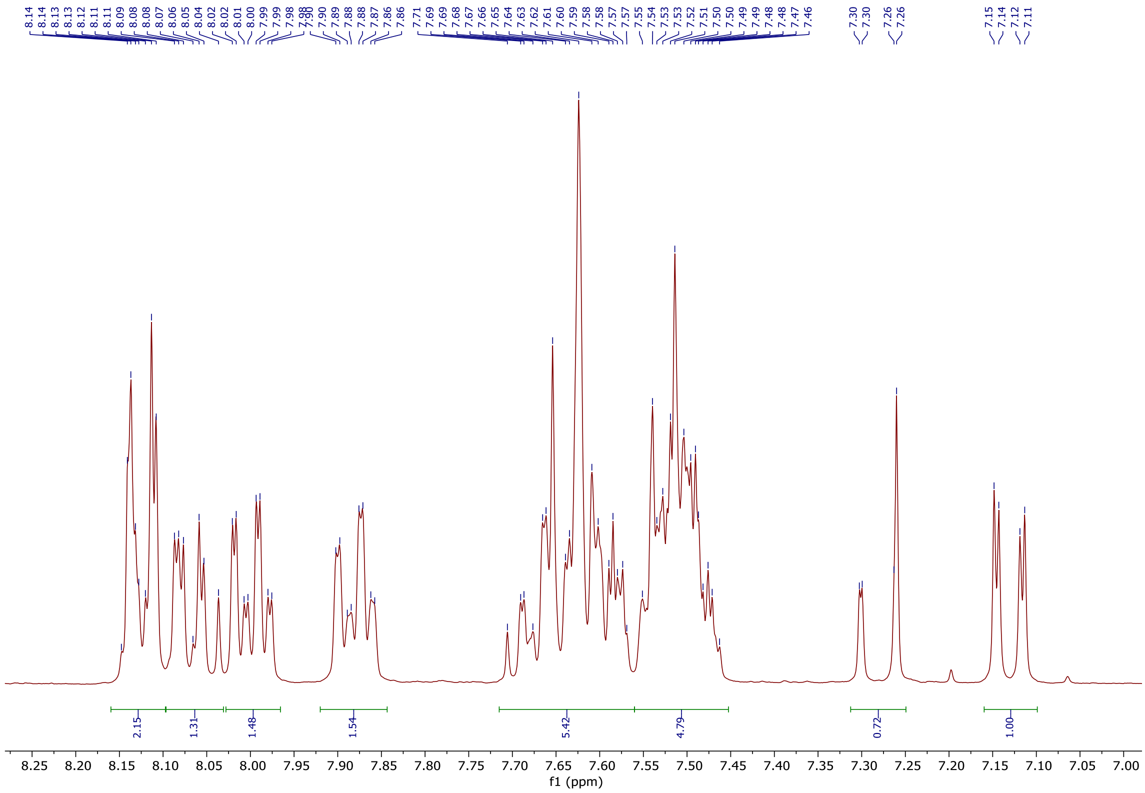

Figure S13. ^{1}H NMR ($CDCl_3$, 300.13 MHz) of **1c**

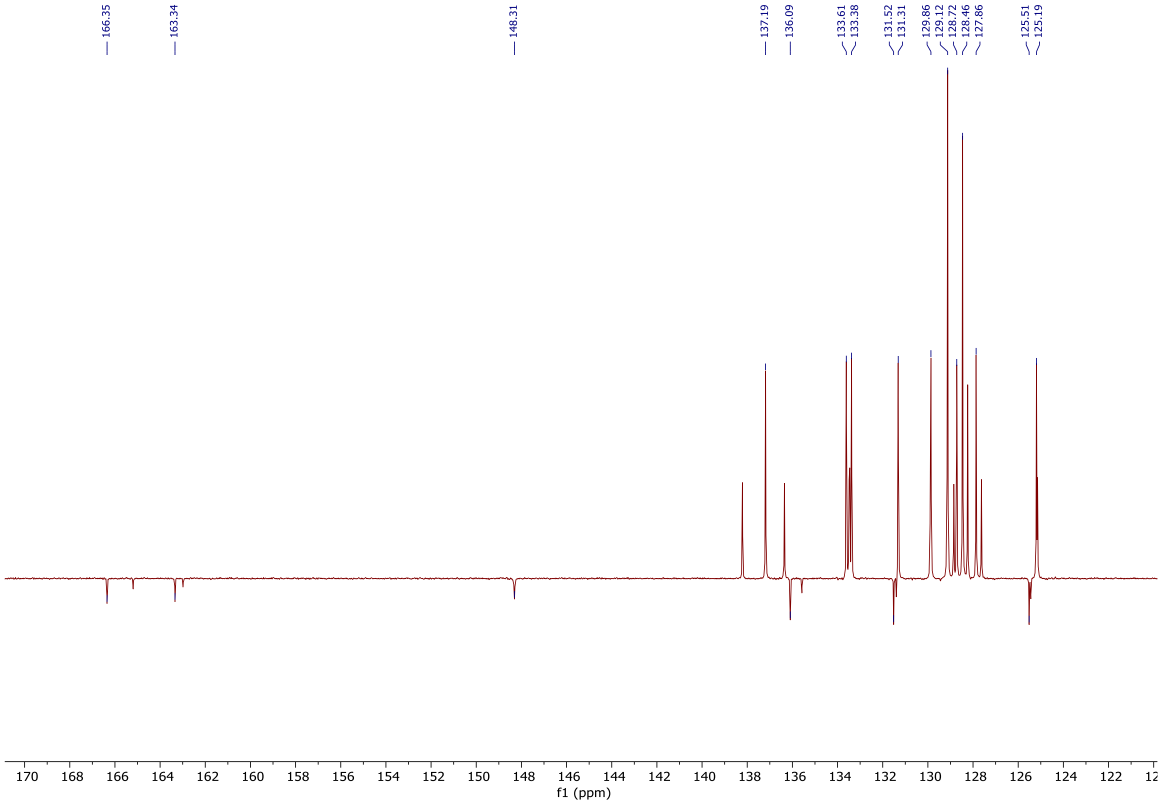

Figure S14. ^{13}C {^{1}H} (APT) NMR ($CDCl_3$, 75.5 MHz) of **1c**

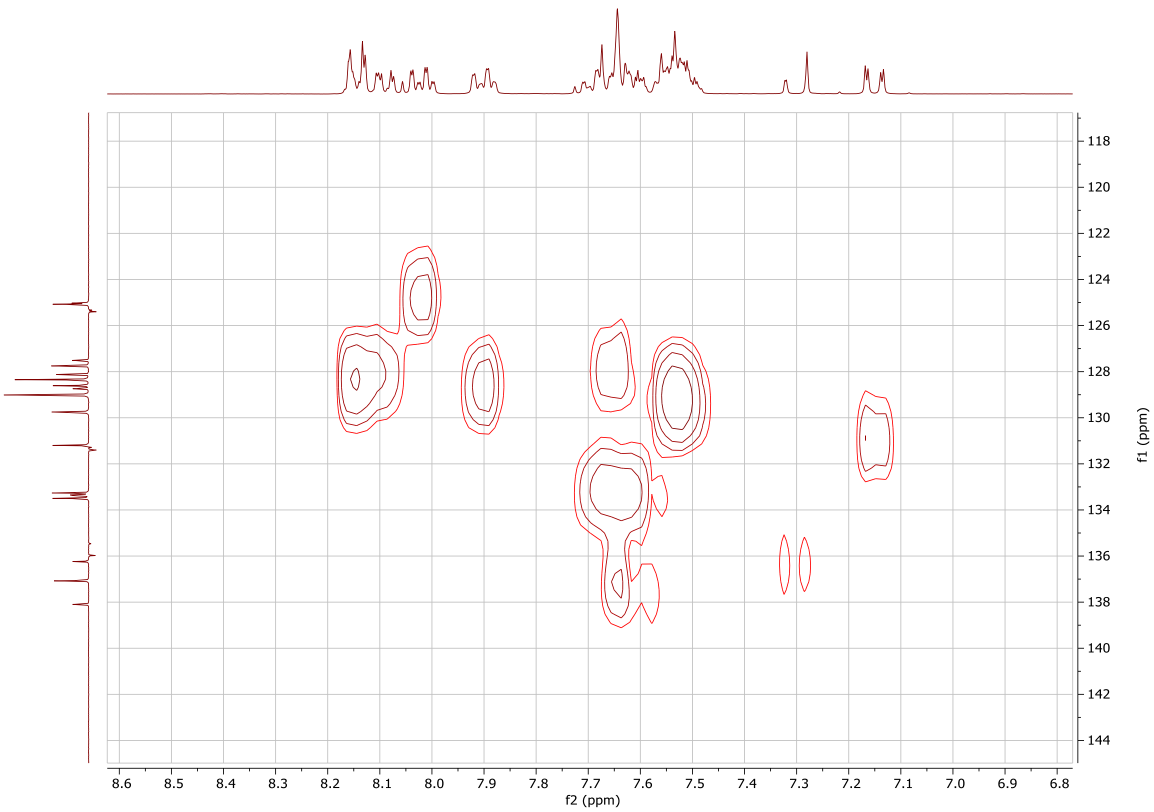

Figure S15. ^{1}H-^{13}C HSQC correlation ($CDCl_3$) of **1c**

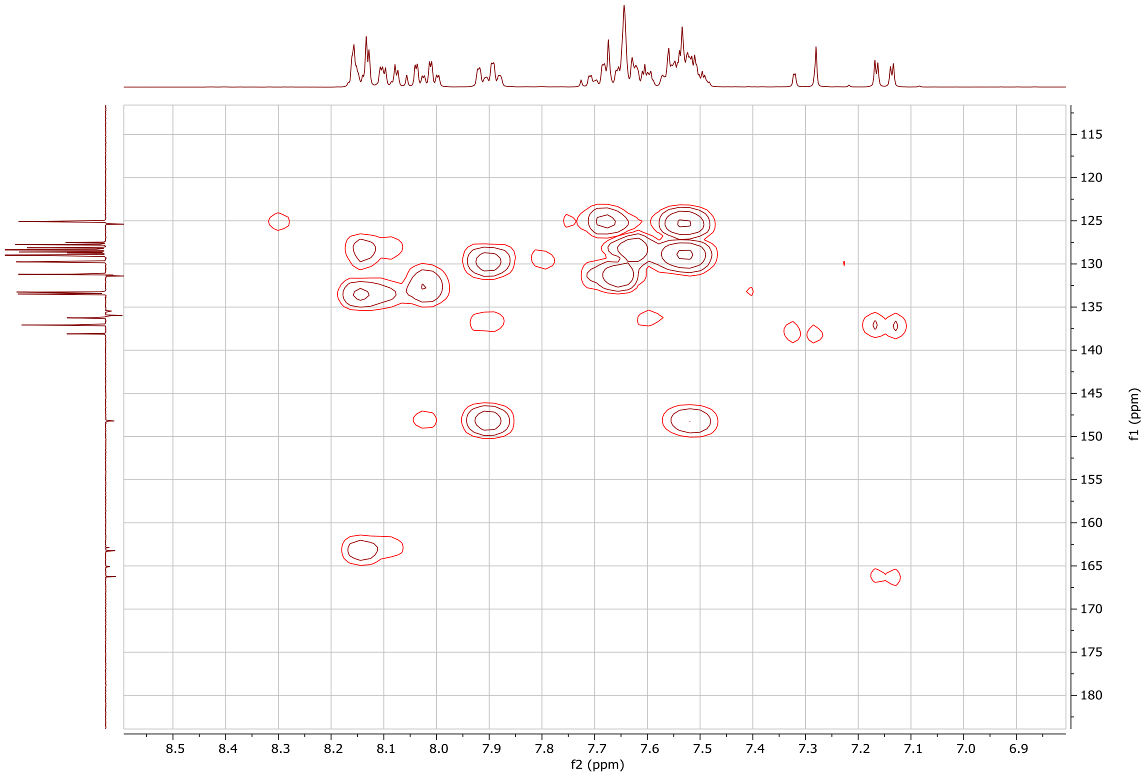

Figure S16. ^{1}H-^{13}C HMBC correlation ($CDCl_3$) of **1c**

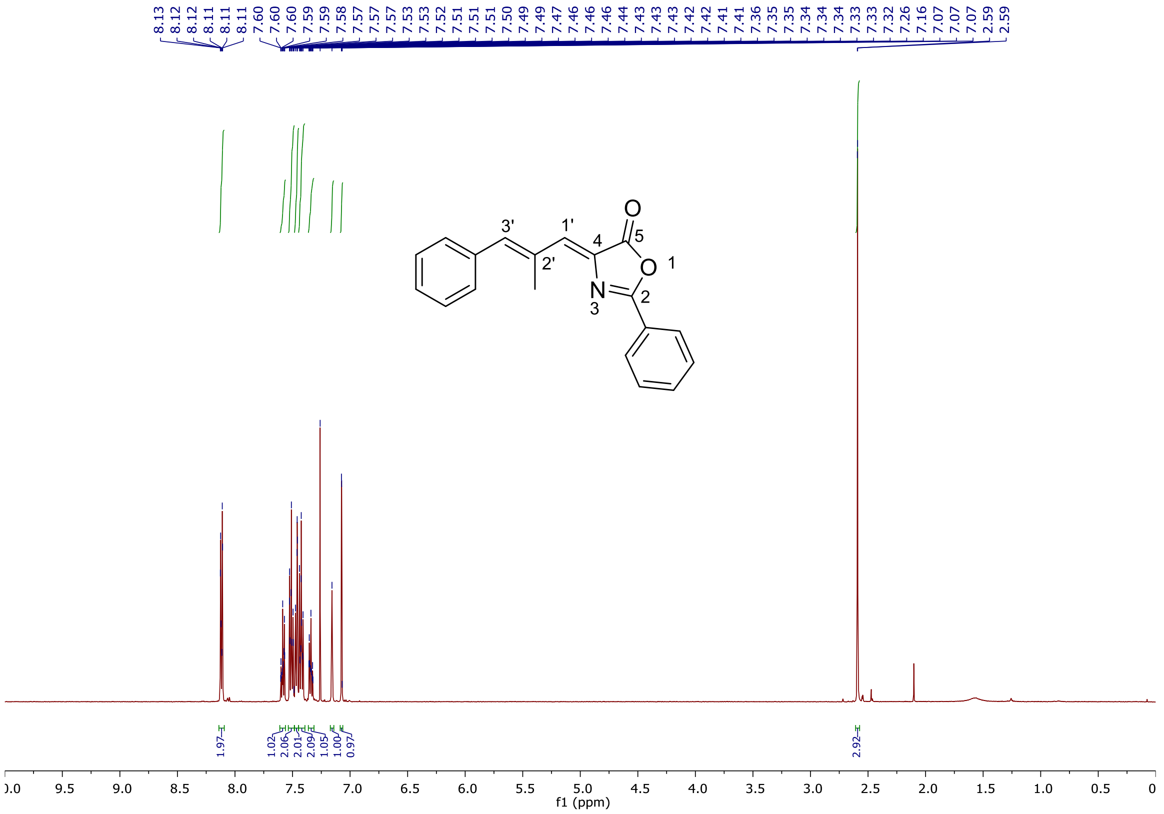

Figure S17. ^{1}H NMR ($CDCl_3$, 500.13 MHz) of **1d**

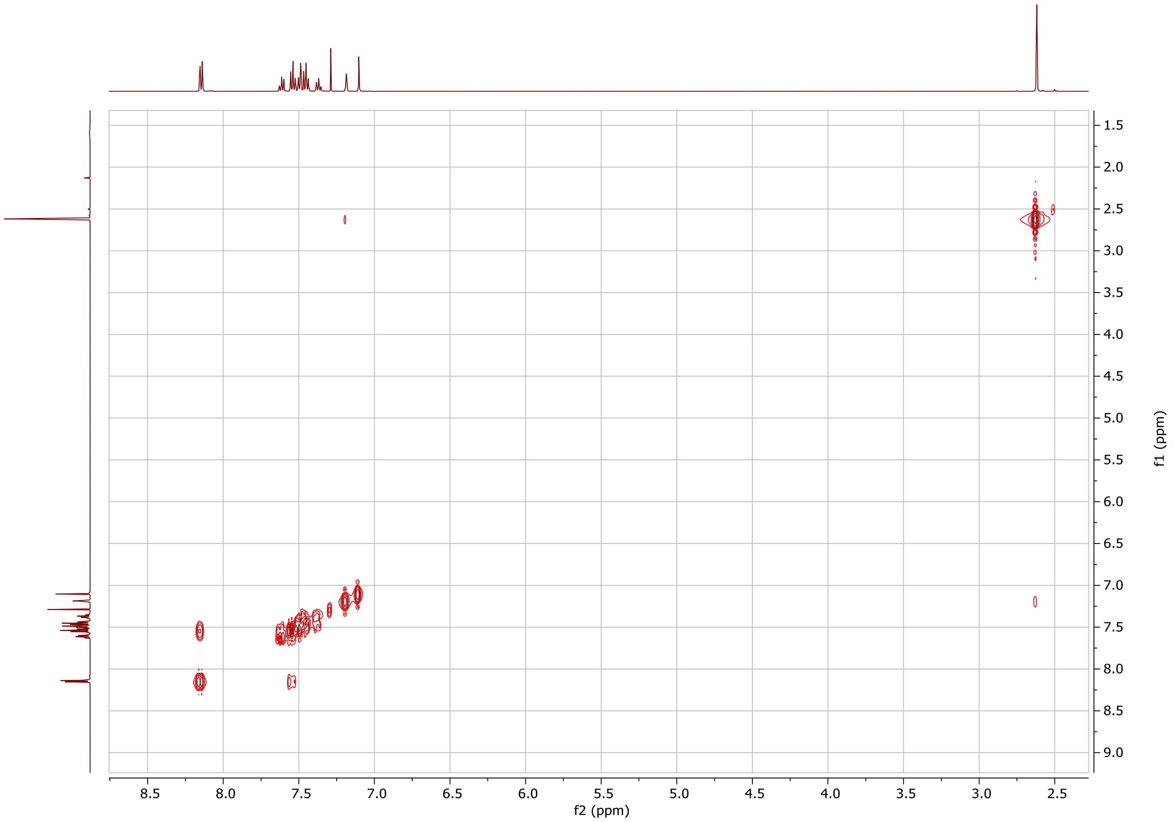

Figure S18. ^{1}H-COSY ($CDCl_3$) of **1d**

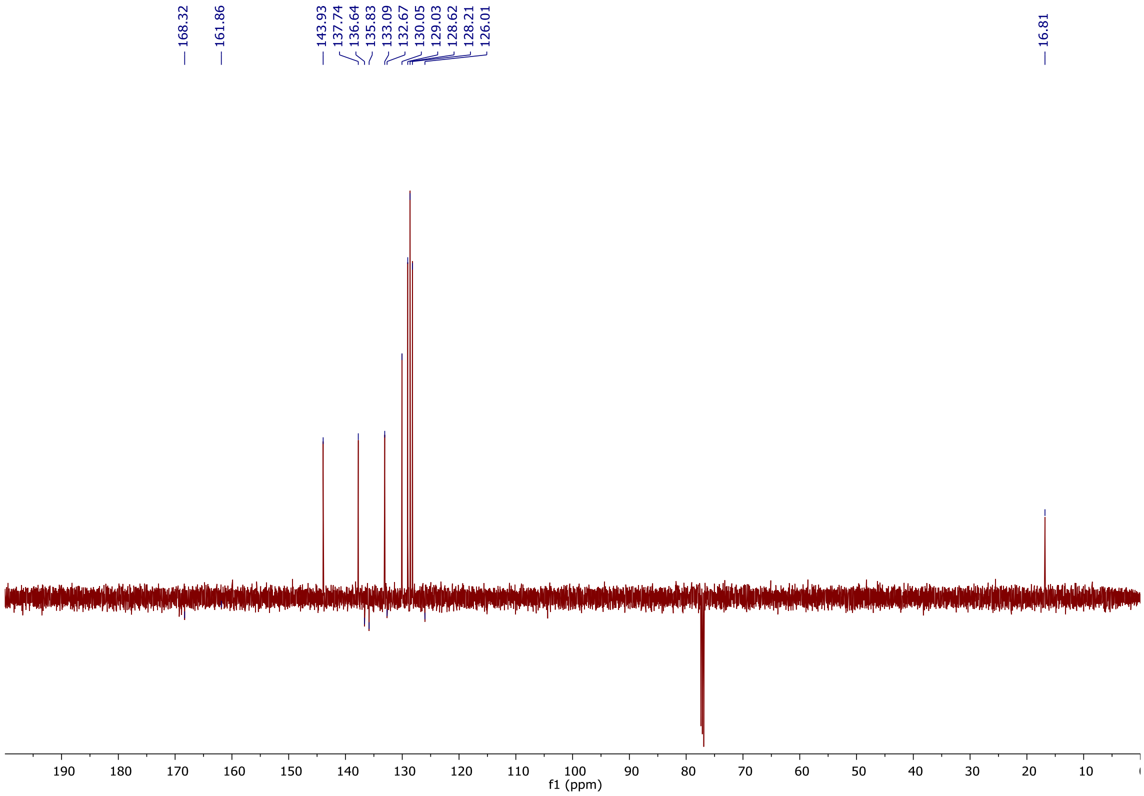

Figure S19. $^{13}C\{^{1}H\}$ (APT) NMR ($CDCl_3$, 125.7 MHz) of **1d**

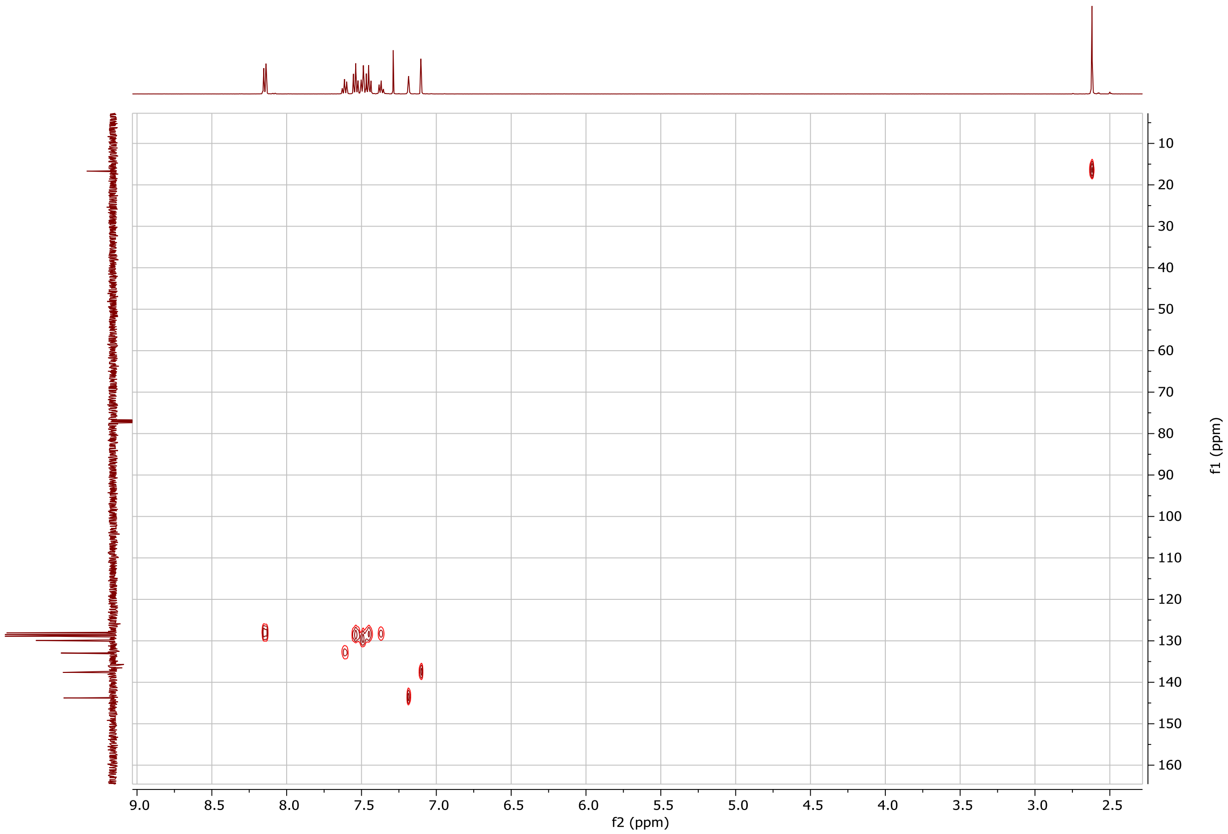

Figure S20. ^{1}H-^{13}C HSQC correlation ($CDCl_3$) of **1d**

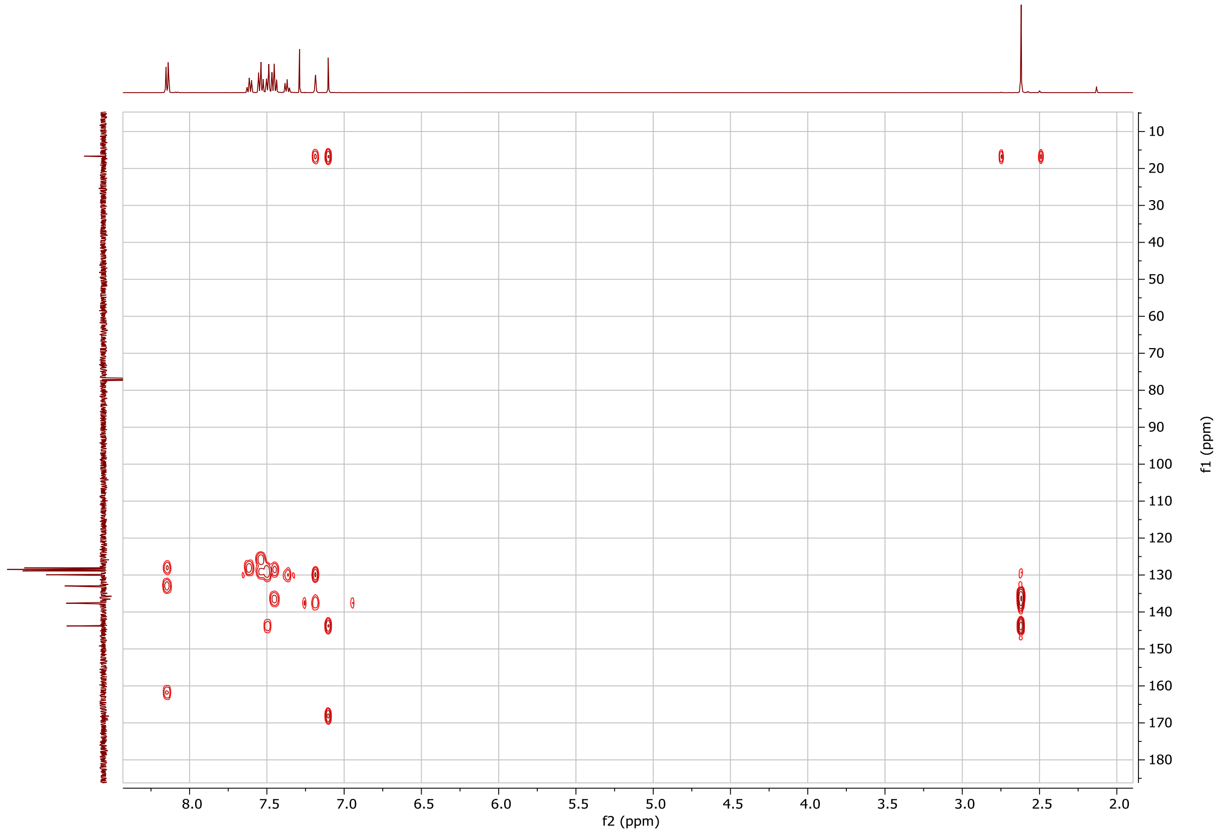

Figure S21. ^{1}H-^{13}C HMBC correlation ($CDCl_3$) of **1d**

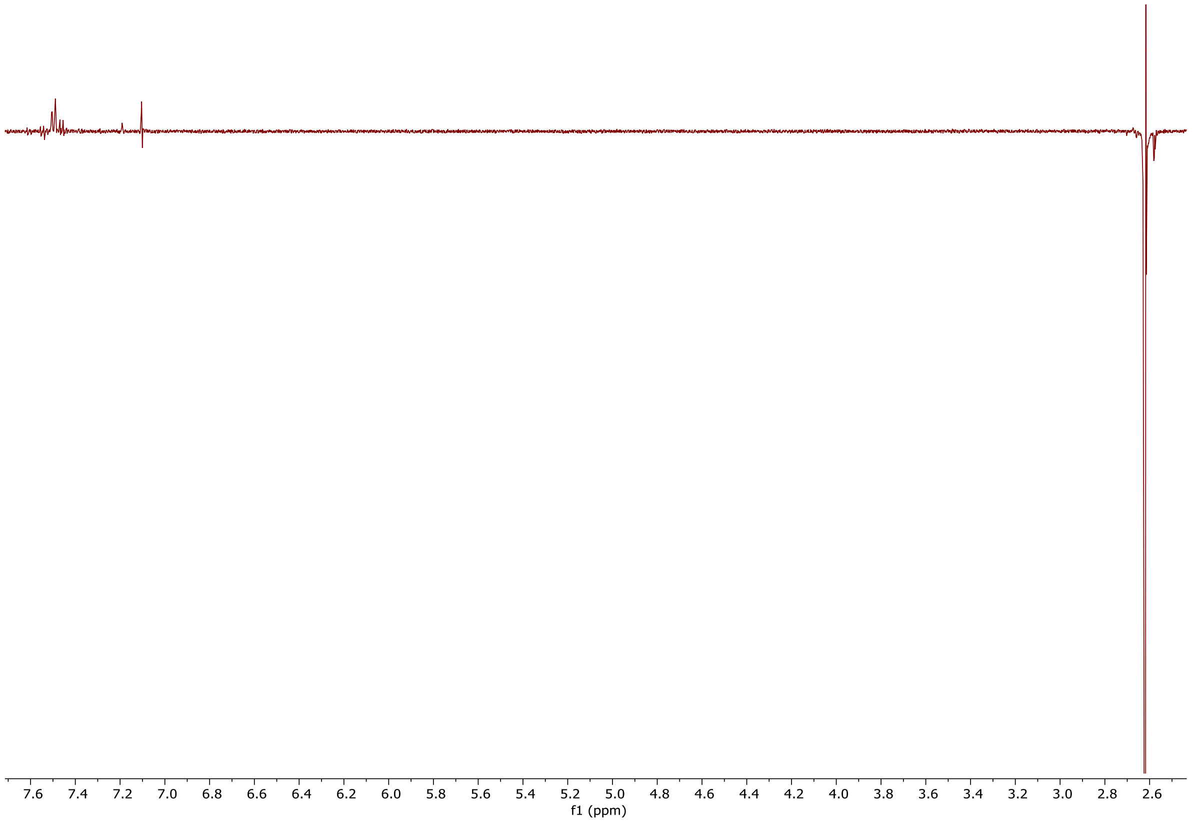

Figure S22. ^{1}H-selective 1D-NOESY ($CDCl_3$) of **1d**. Selective irradiation of the alpha-methyl signal.

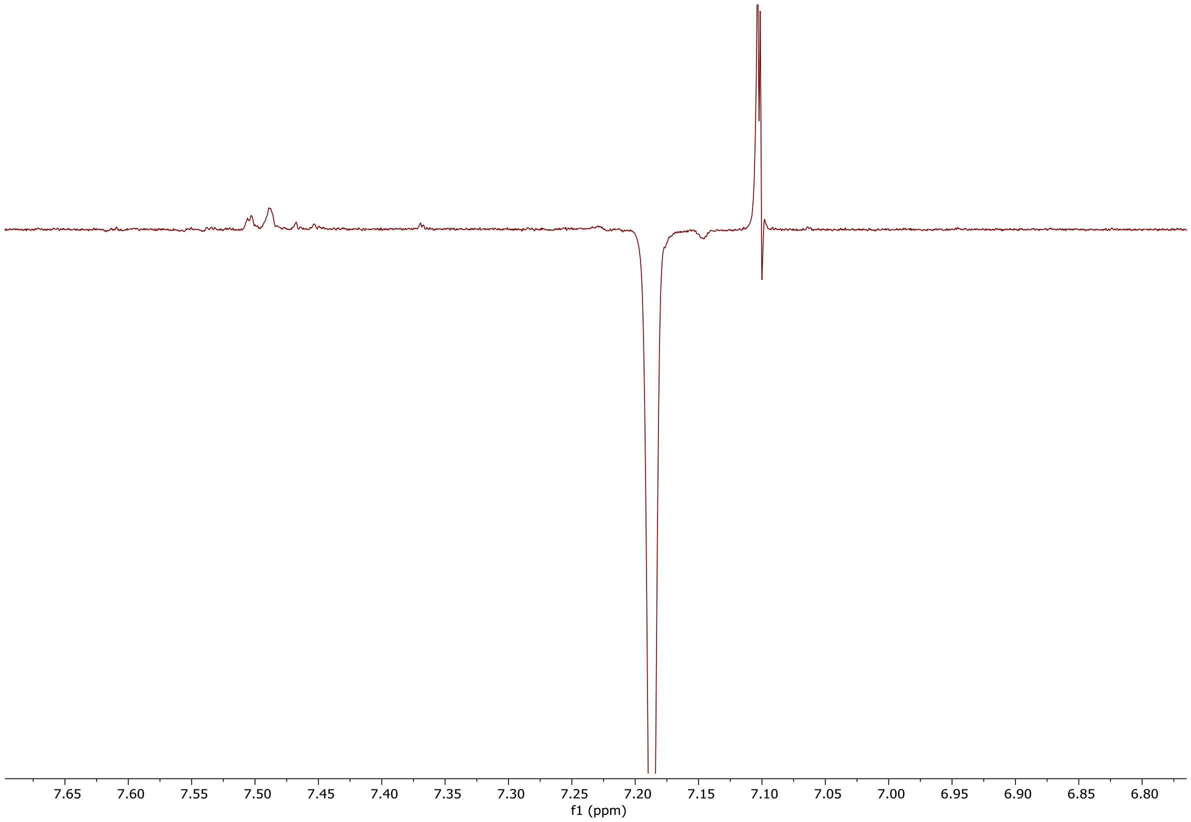

Figure S23. ^{1}H-selective 1D-NOESY ($CDCl_3$) of **1d.** Selective irradiation of H3' signal.

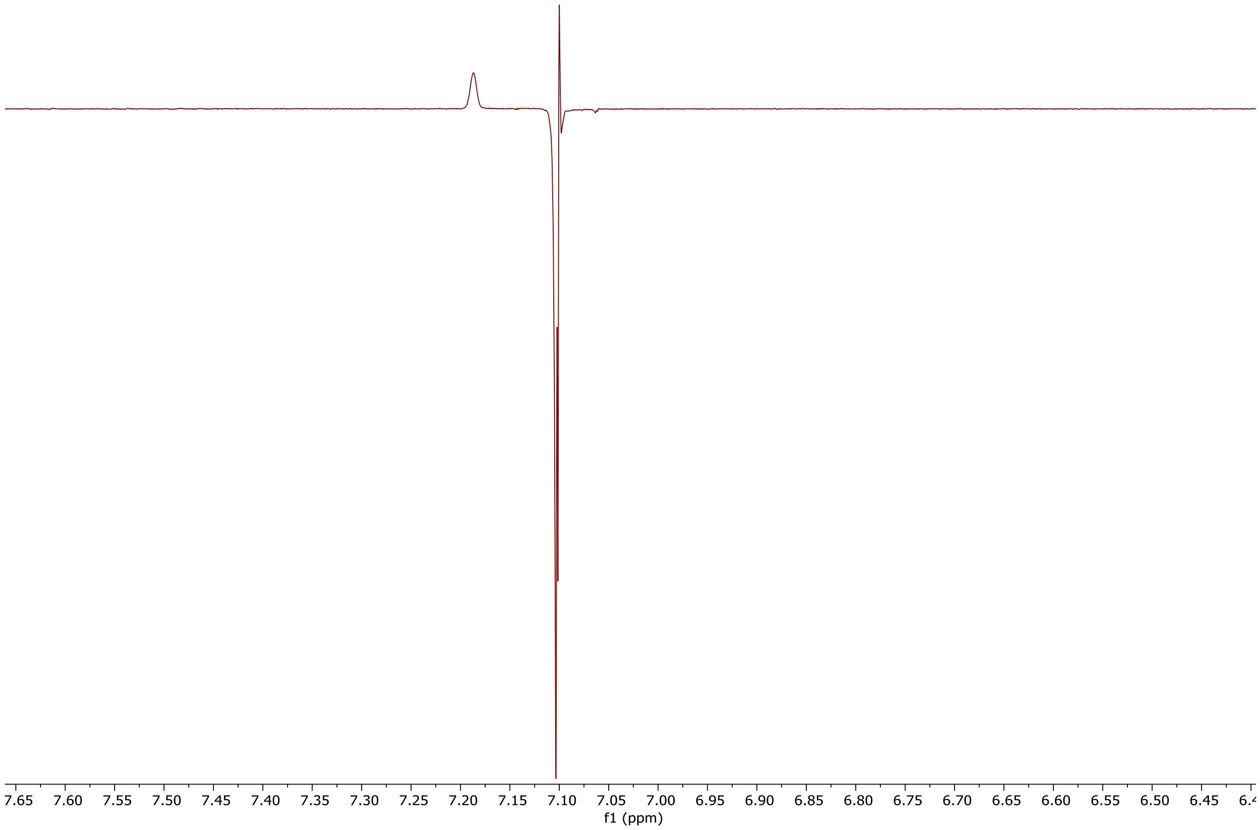

Figure S24. ^{1}H-selective 1D-NOESY ($CDCl_3$) of **1d.** Selective irradiation of H1' signal.

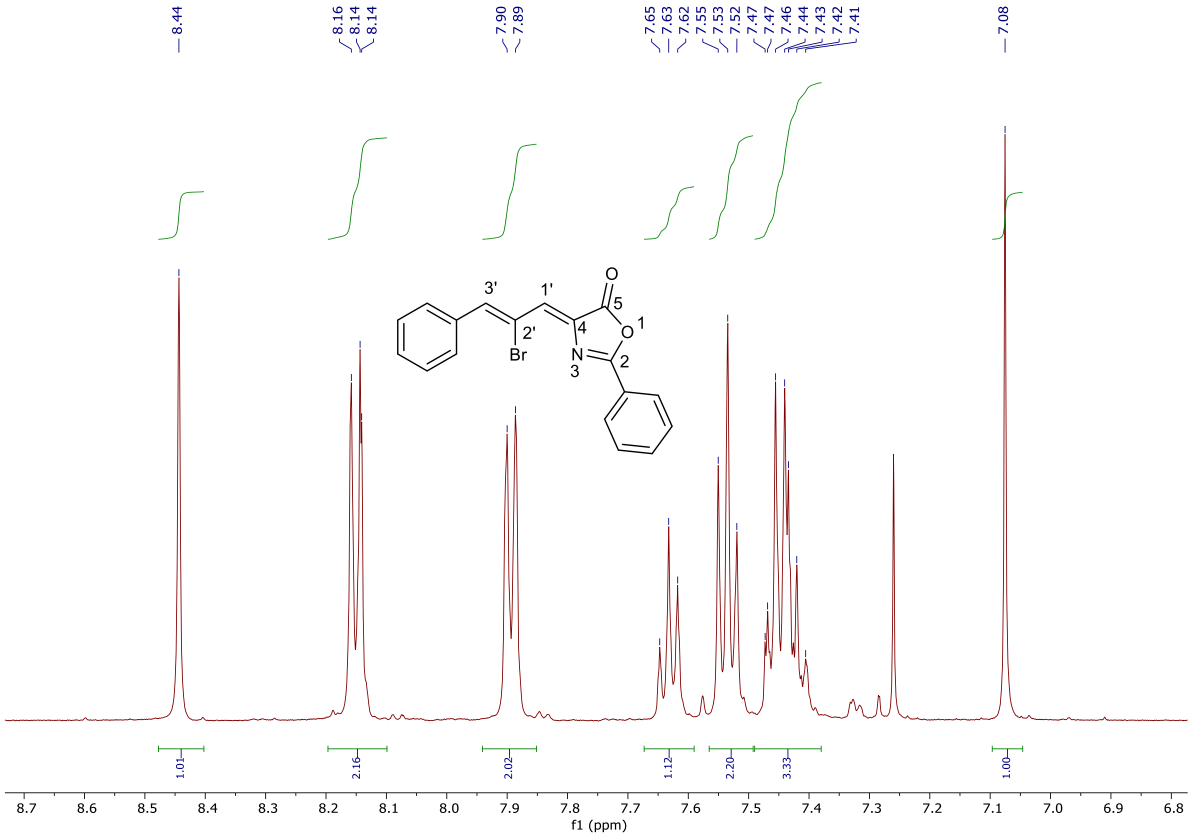

Figure S25. ^{1}H NMR ($CDCl_3$, 500.13 MHz) of **1e**

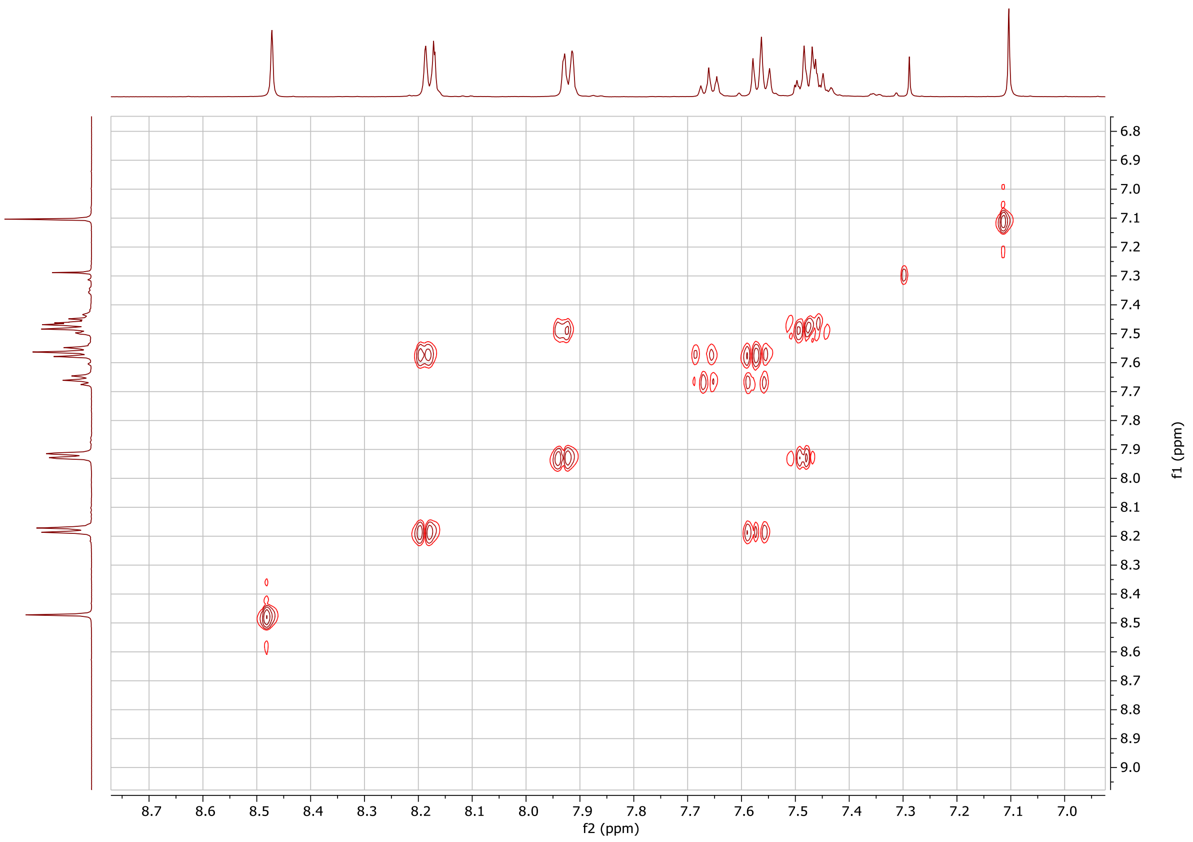

Figure S26. ^{1}H-COSY ($CDCl_3$) of **1e**

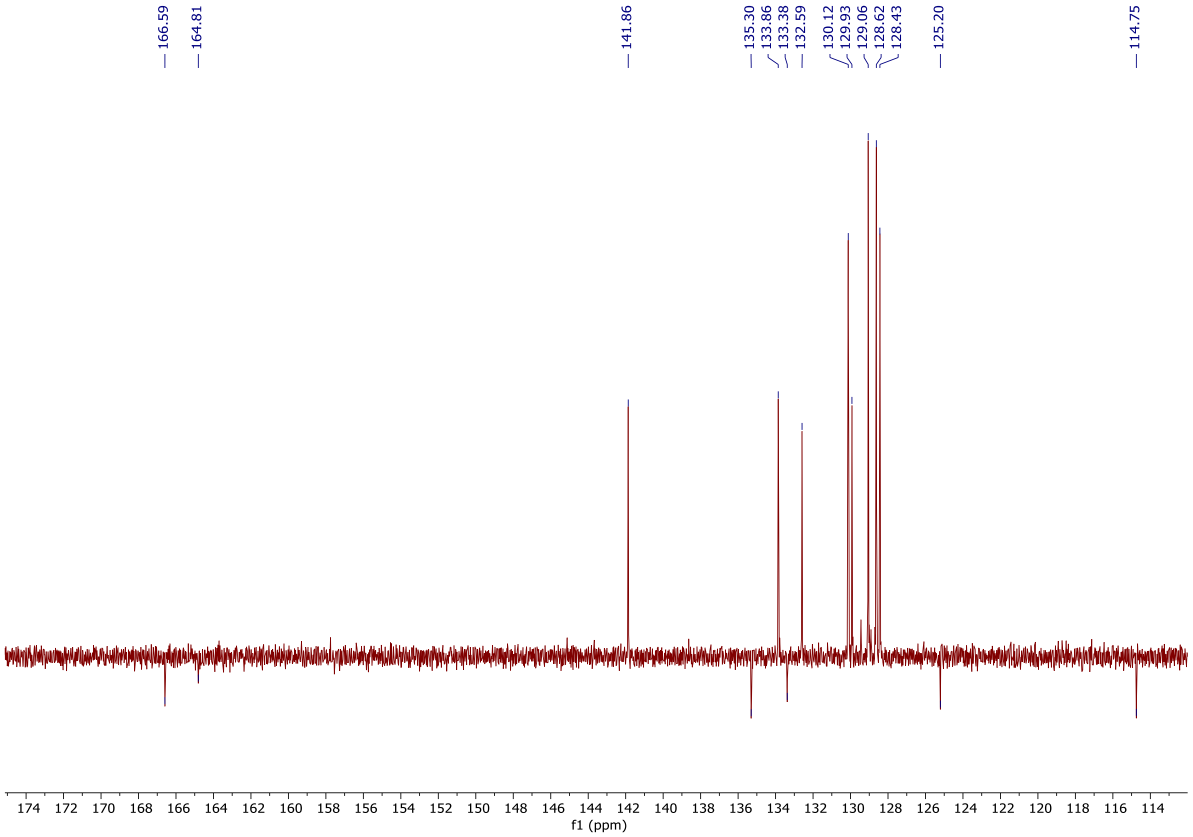

Figure S27. ^{13}C {^{1}H} (APT) NMR ($CDCl_3$, 125.7 MHz) of **1e**

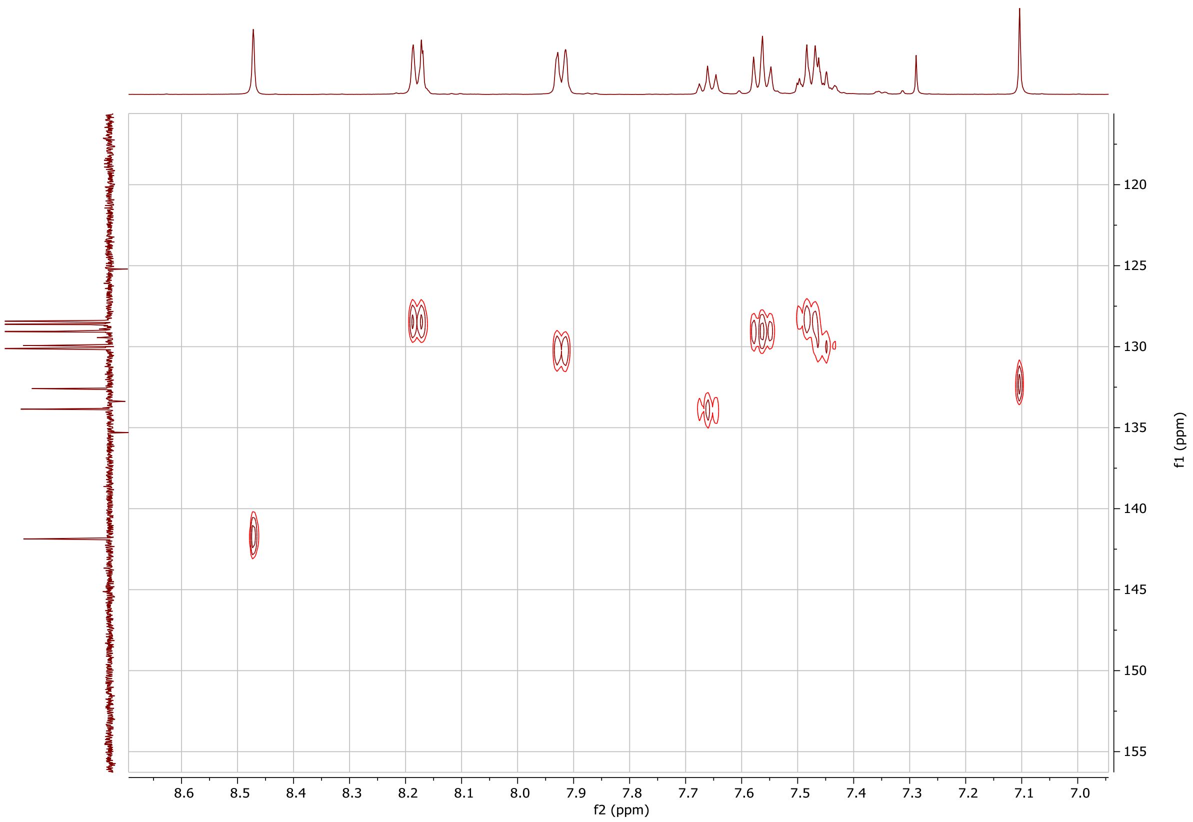

Figure S28. ^{1}H-^{13}C HSQC correlation ($CDCl_3$) of **1e**

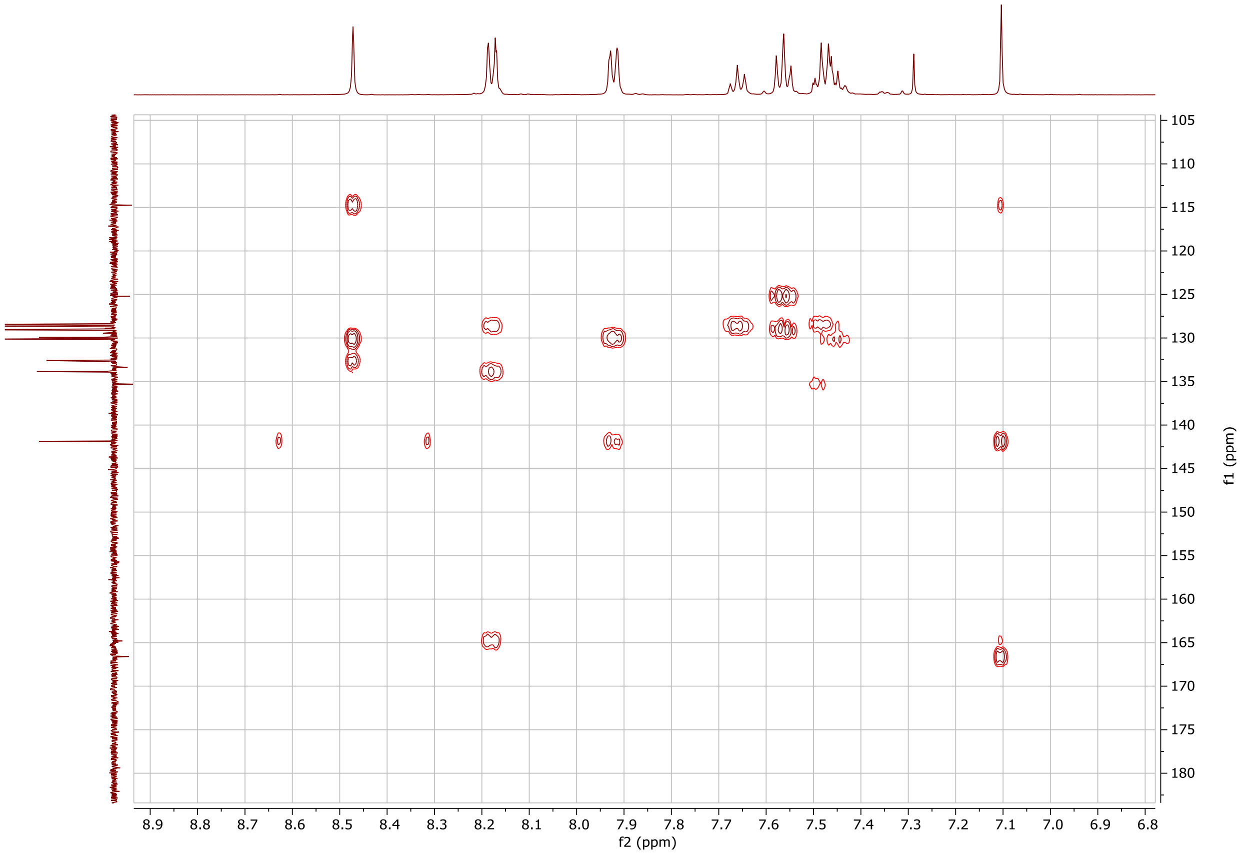

Figure S29. ^{1}H-^{13}C HMBC correlation ($CDCl_3$) of **1e**

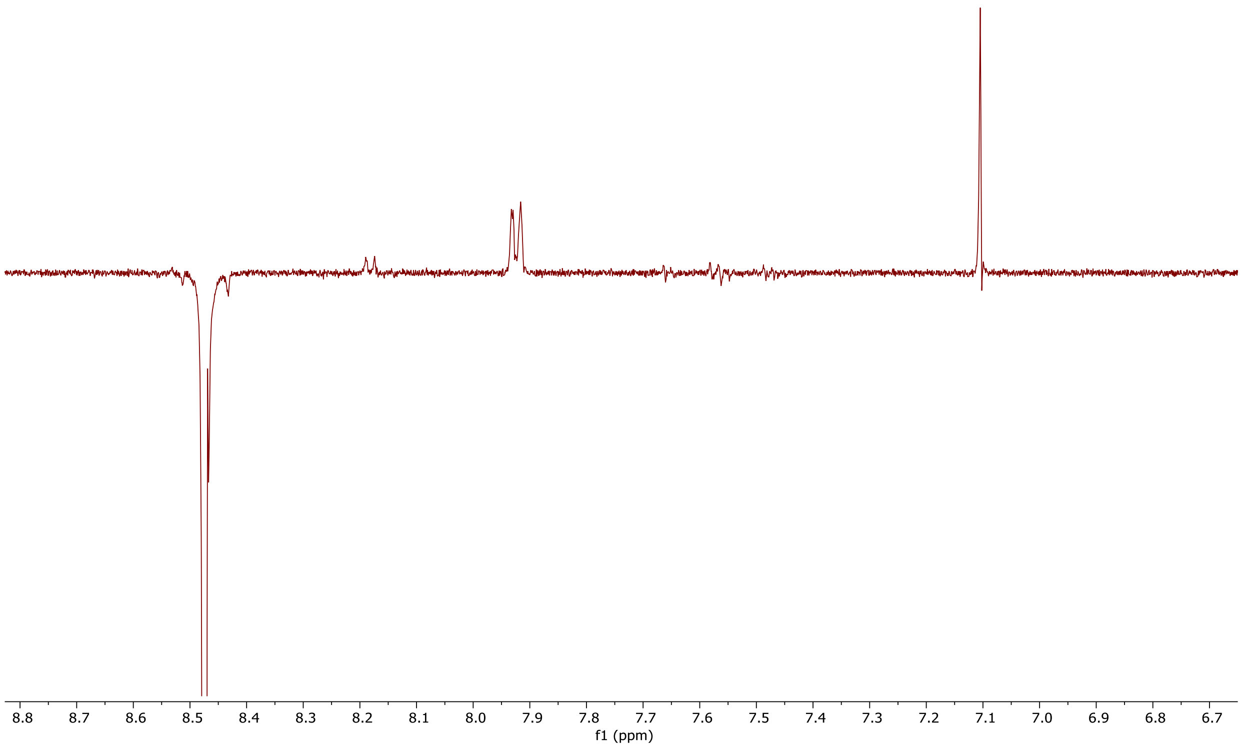

Figure S30. ^{1}H-selective 1D-NOESY ($CDCl_3$) of **1e.** Selective irradiation of the H3' signal

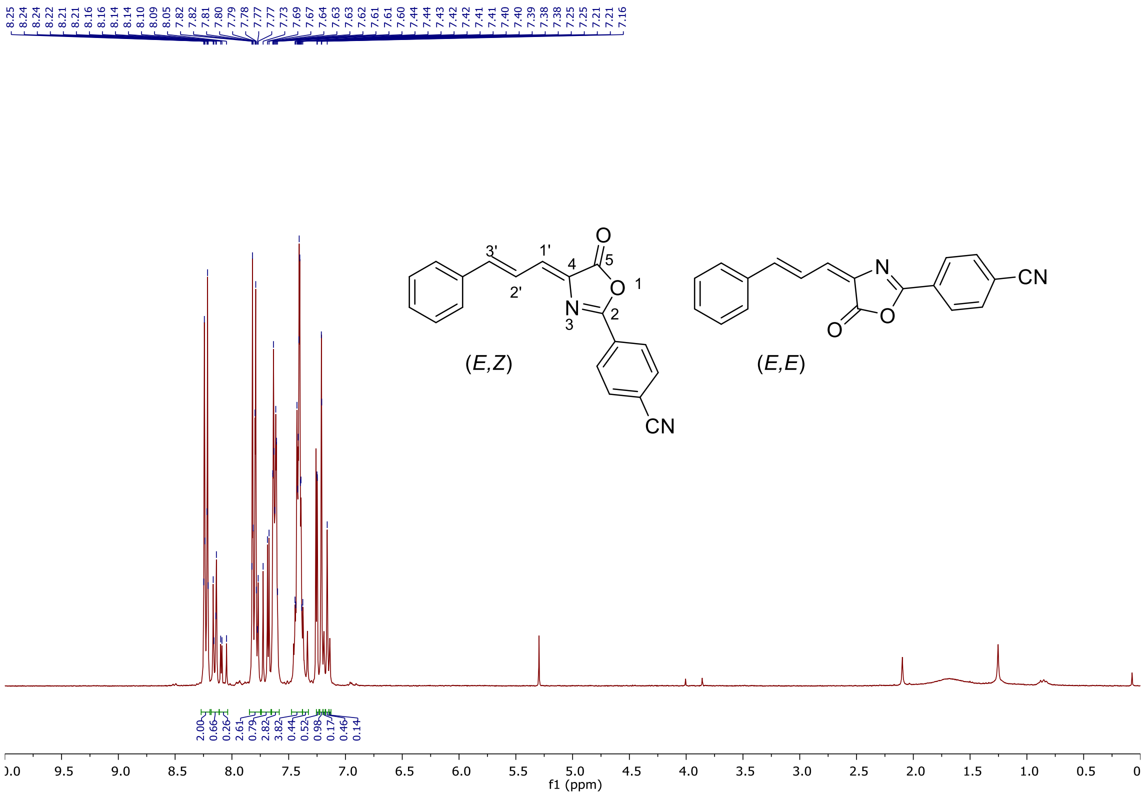

Figure S31. ^{1}H NMR ($CDCl_3$, 300.13 MHz) of **1f**

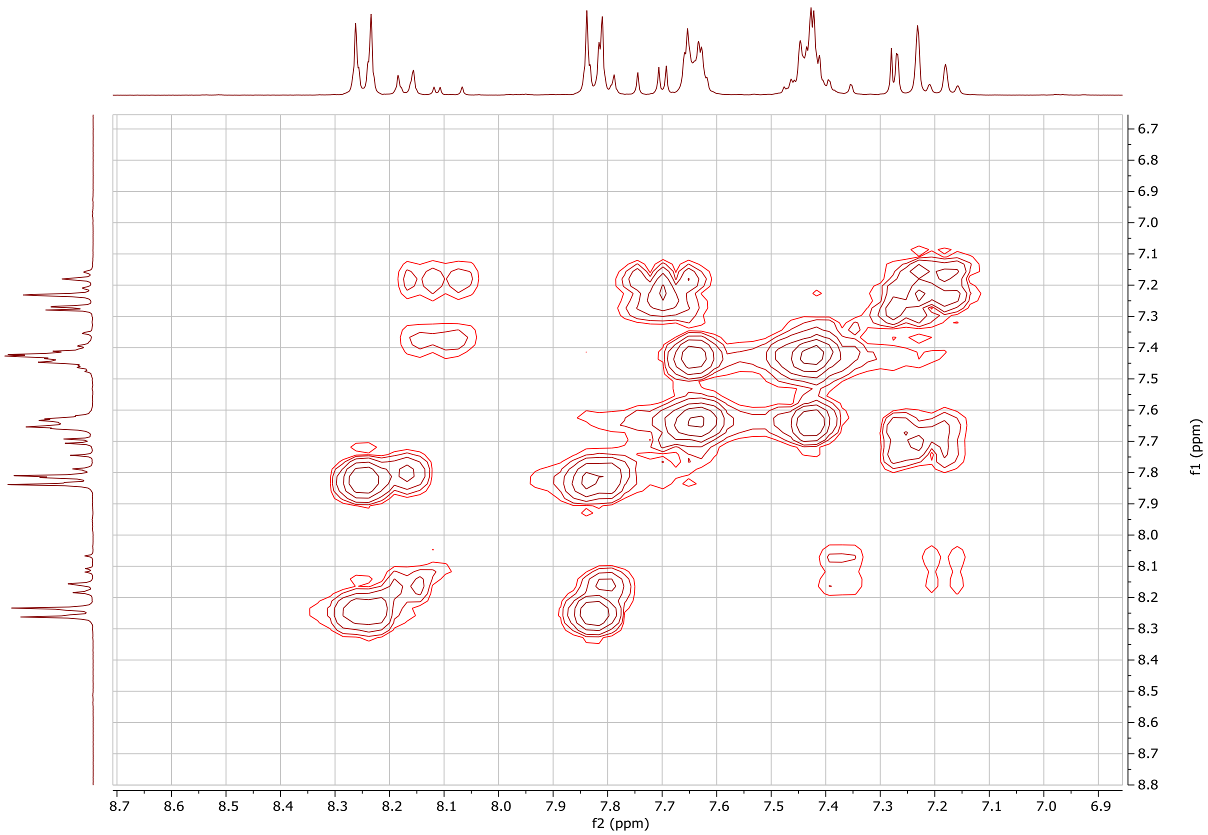

Figure S32. ^{1}H-COSY ($CDCl_3$) of **1f**

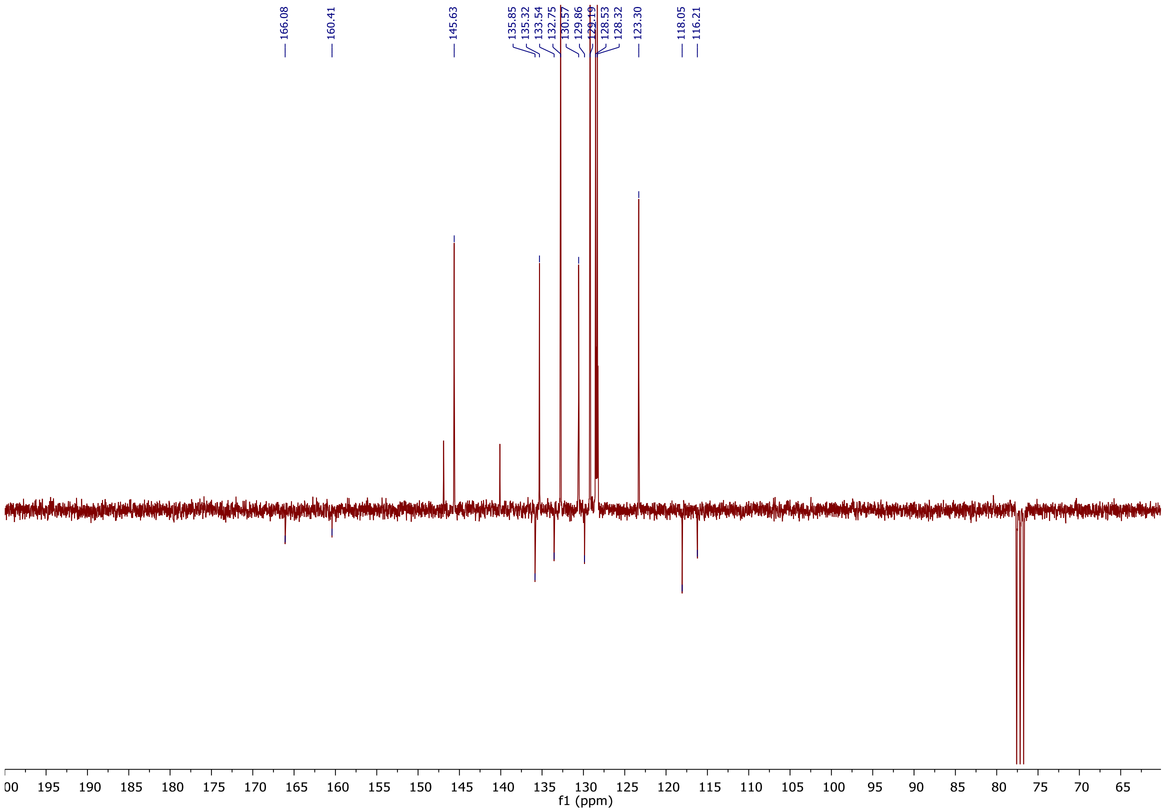

Figure S33. ^{13}C {^{1}H} (APT) NMR ($CDCl_3$, 75.5 MHz) of **1f**

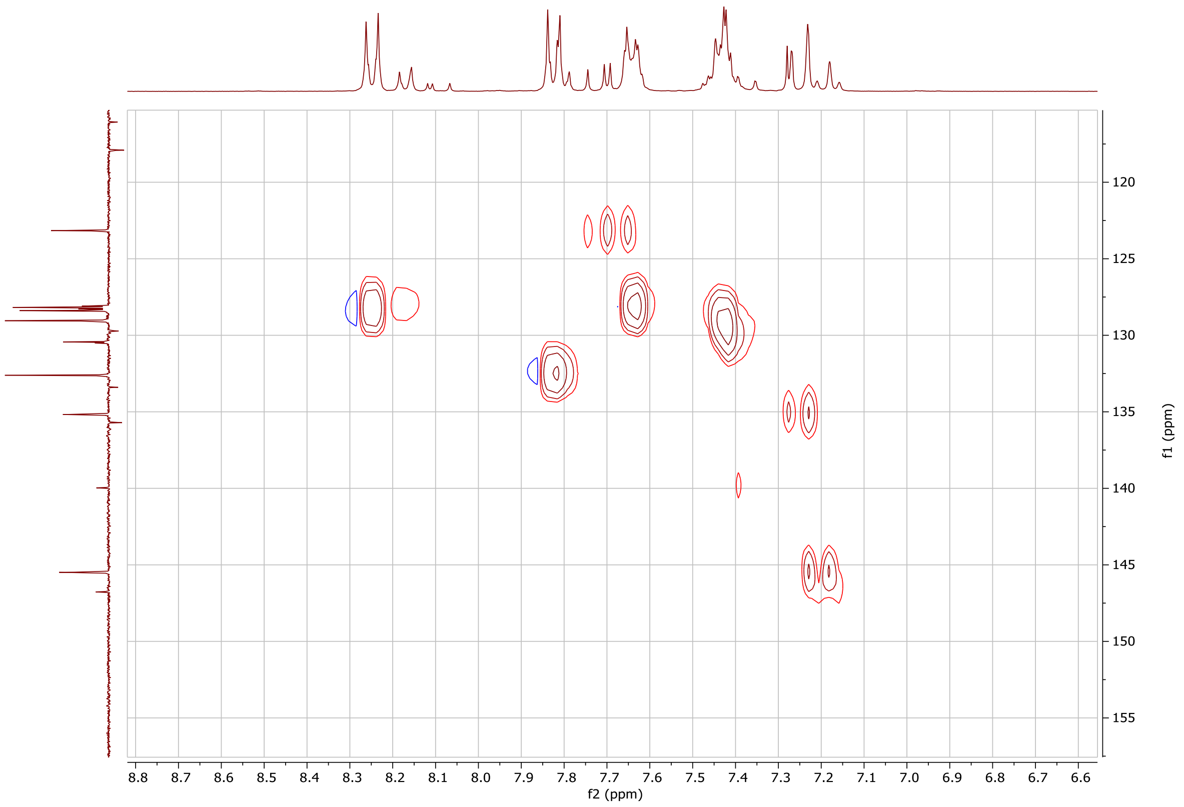

Figure S34. ^{1}H-^{13}C HSQC correlation ($CDCl_3$) of **1f**

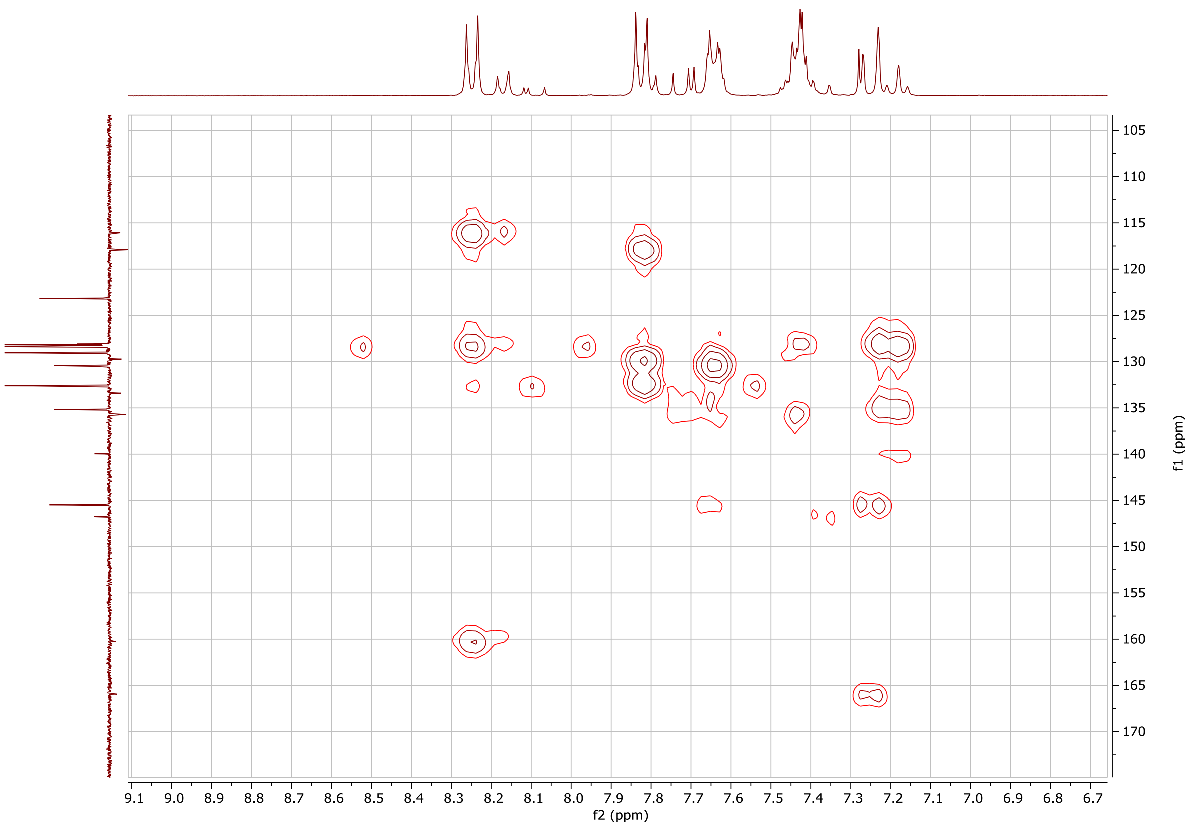

Figure S35. ^{1}H-^{13}C HMBC correlation ($CDCl_3$) of **1f**

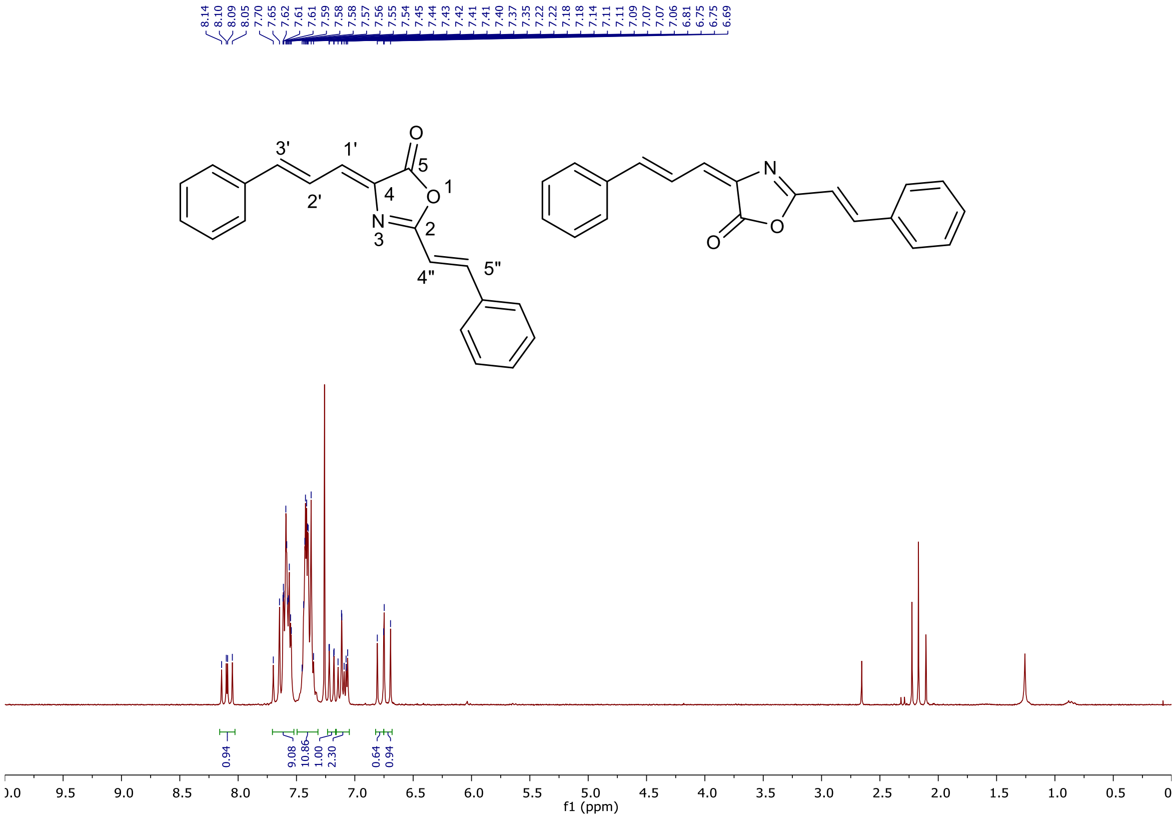

Figure S36. ^{1}H NMR ($CDCl_3$, 300.13 MHz) of **1g**

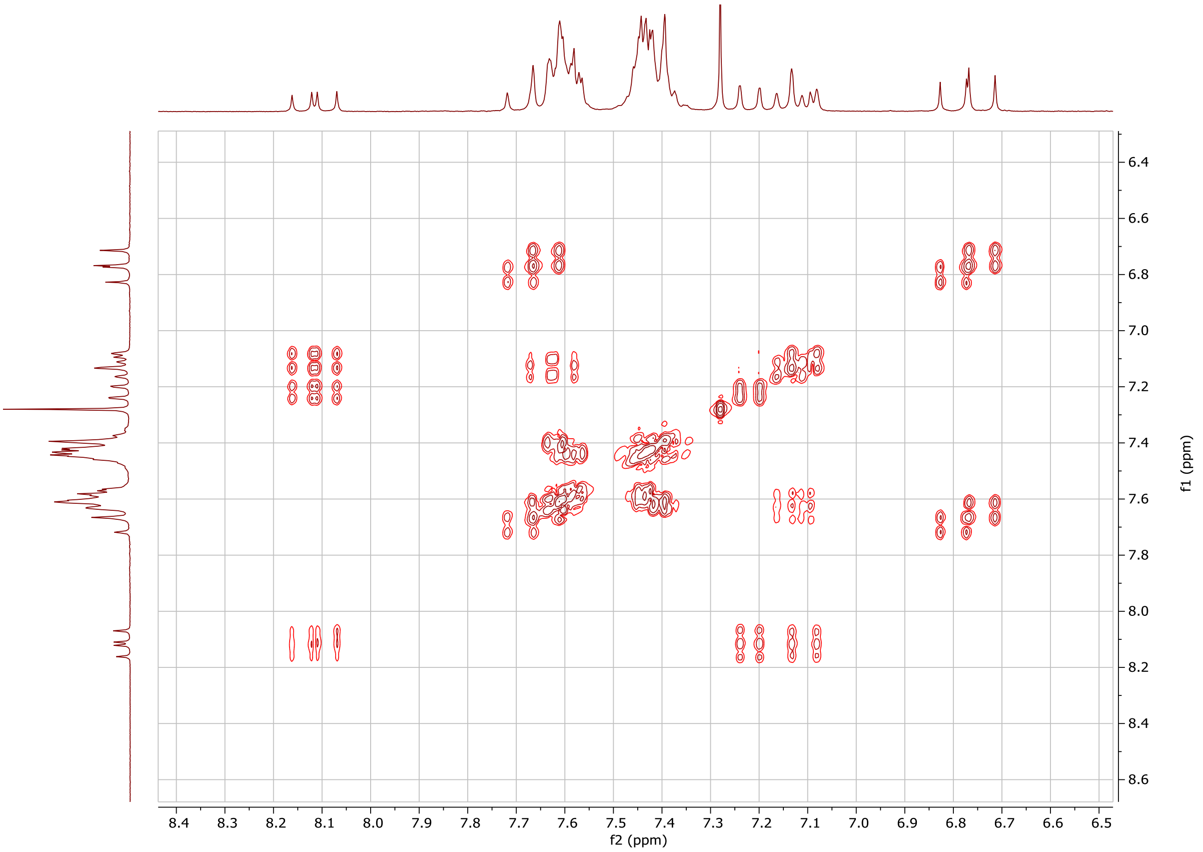

Figure S37. ^{1}H-COSY ($CDCl_3$) of **1g**

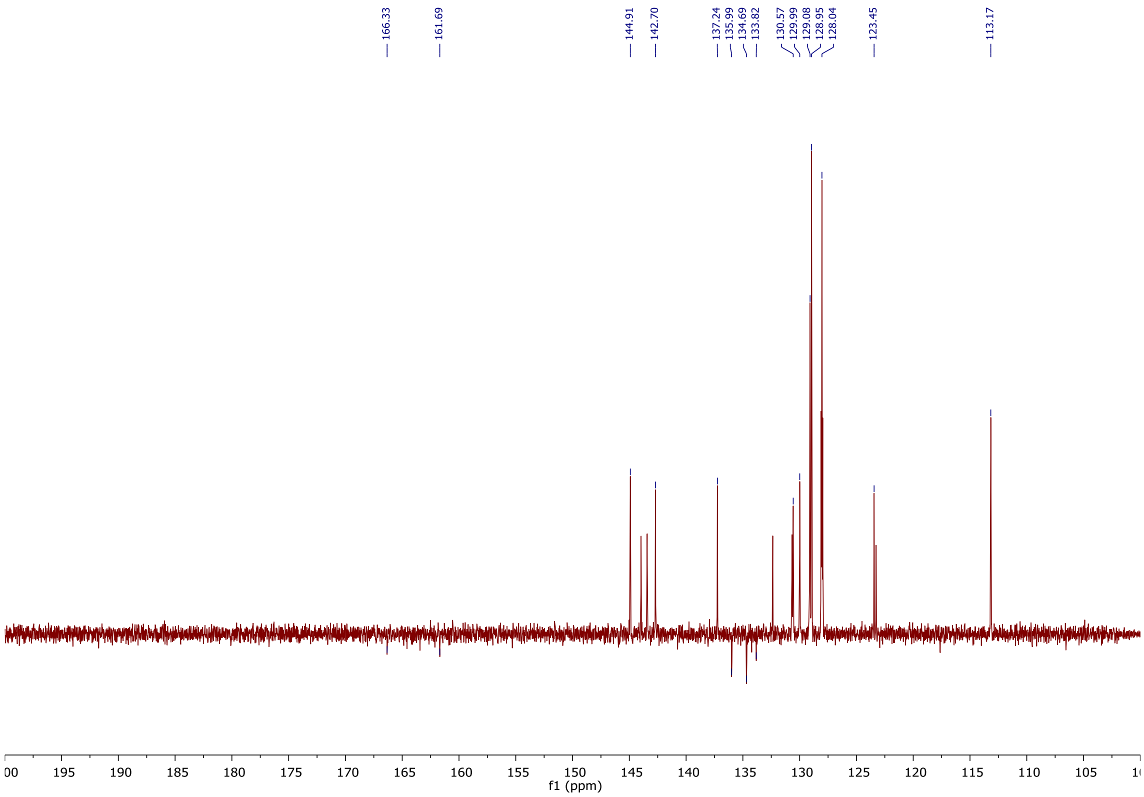

Figure S38. ^{13}C {^{1}H} (APT) NMR ($CDCl_3$, 75.5 MHz) of **1g**

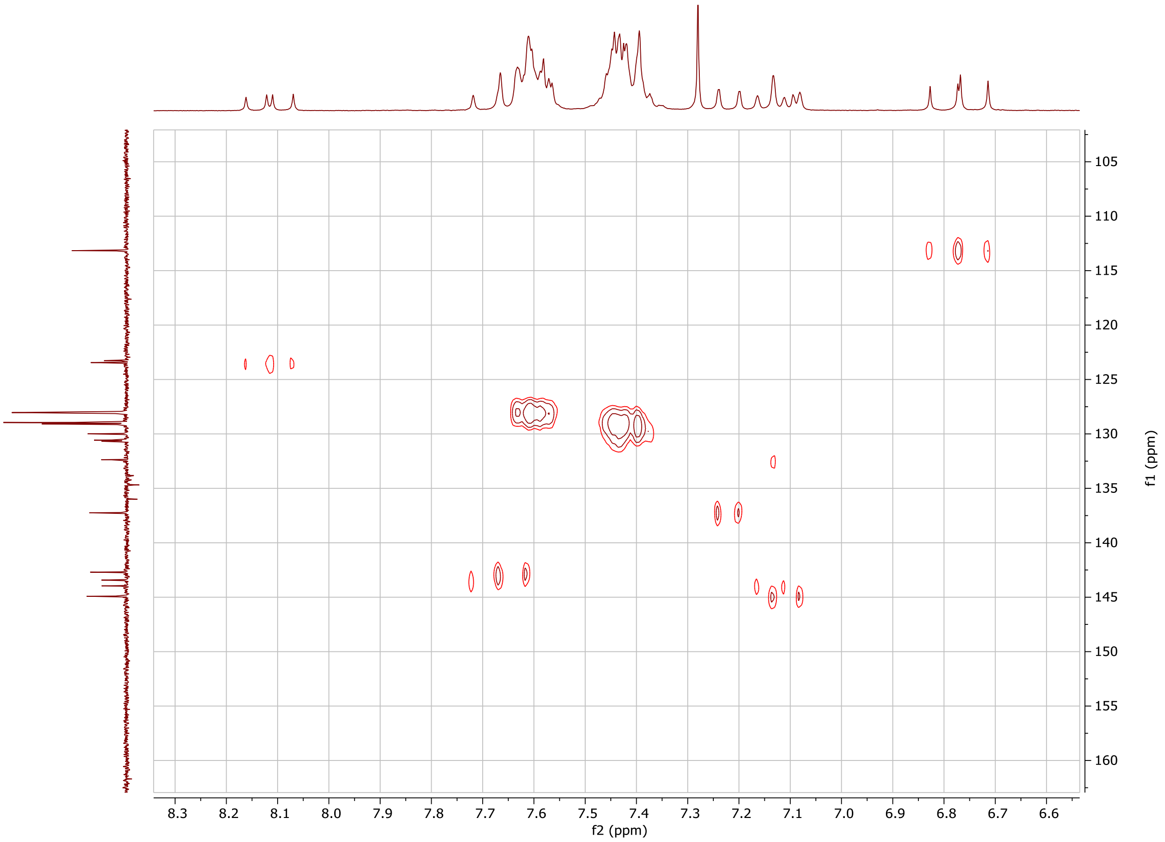

Figure S39. ^{1}H-^{13}C HSQC correlation ($CDCl_3$) of **1g**

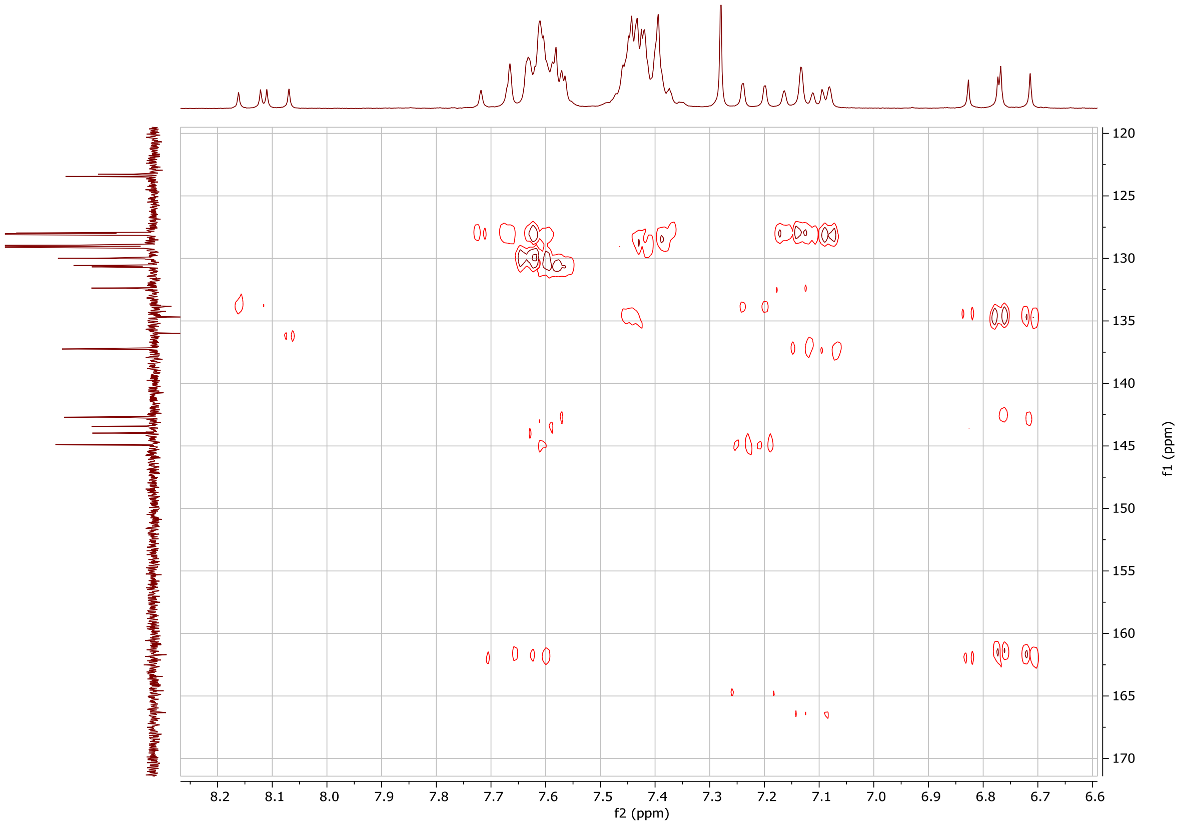

Figure S40. ^{1}H-^{13}C HMBC correlation ($CDCl_3$) of **1g**

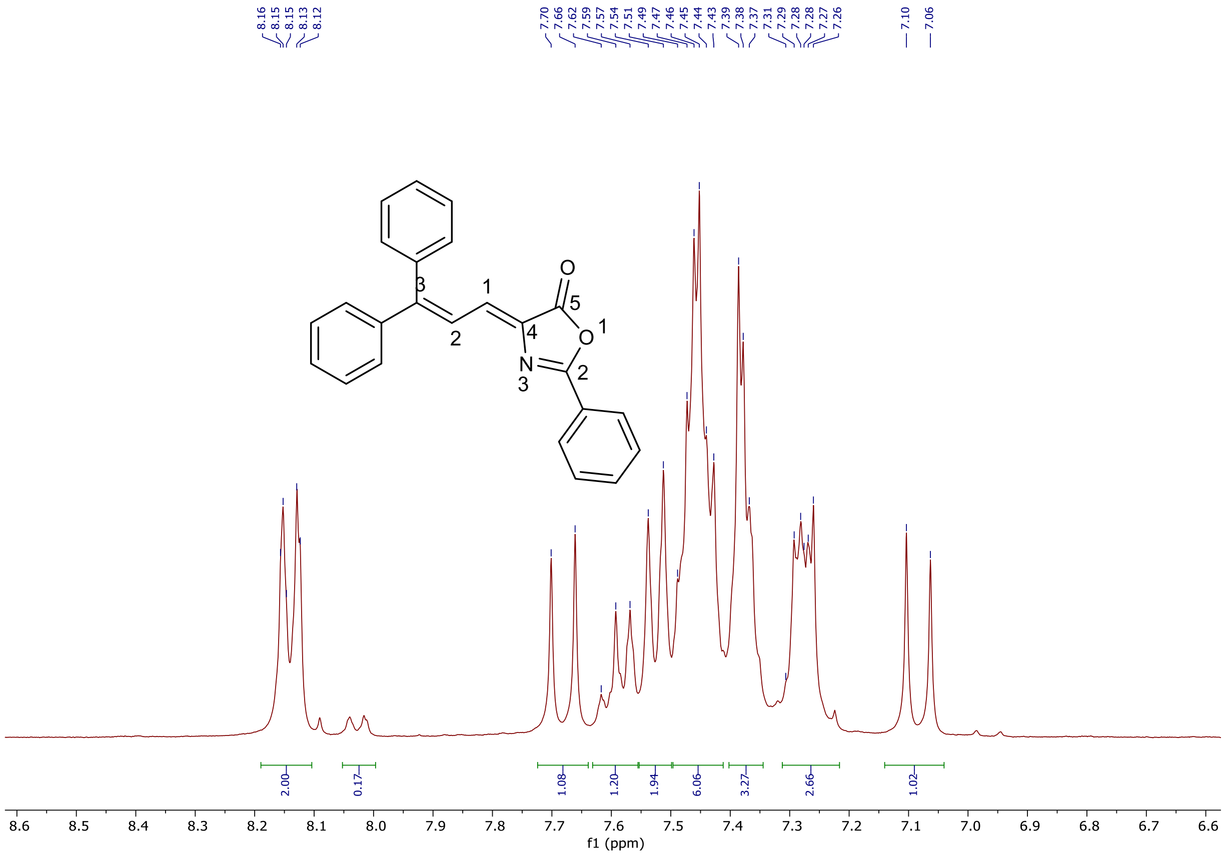

Figure S41. ^{1}H NMR ($CDCl_3$, 300.13 MHz) of **1h**

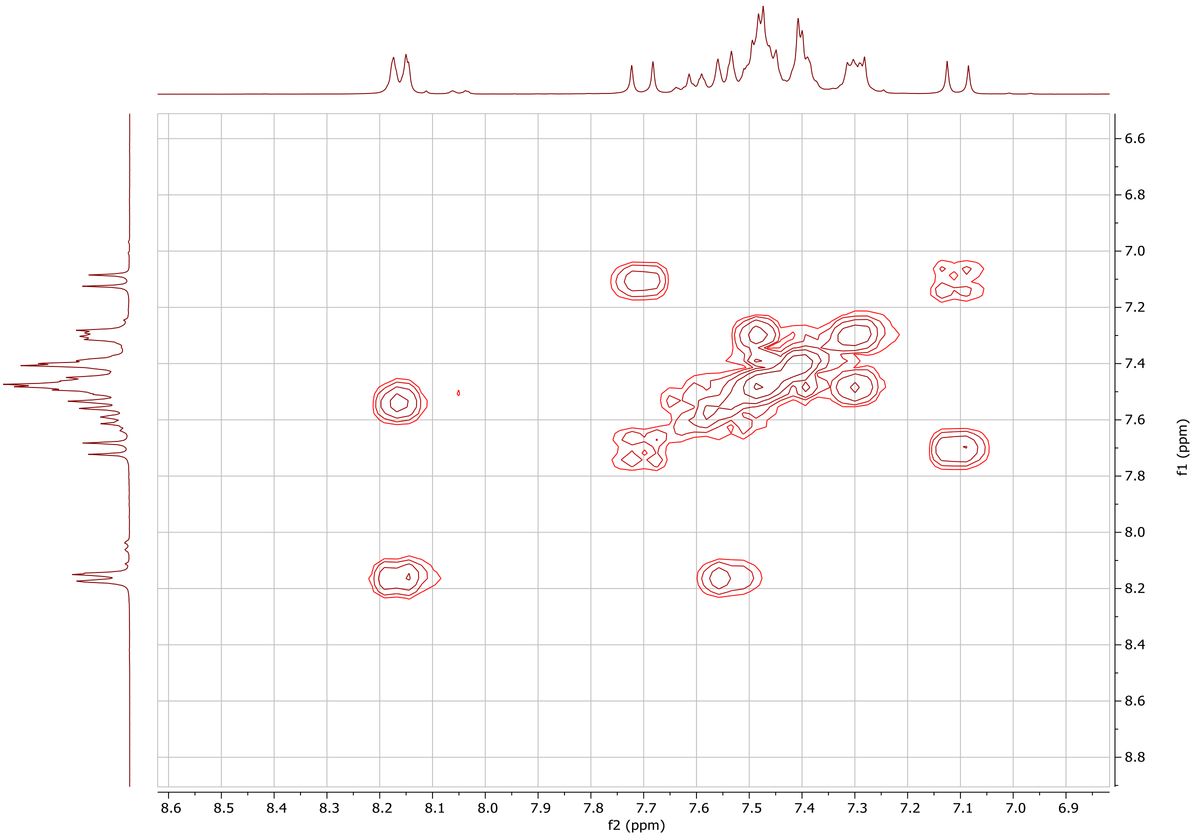

Figure S42. ^{1}H-COSY ($CDCl_3$) of **1h**

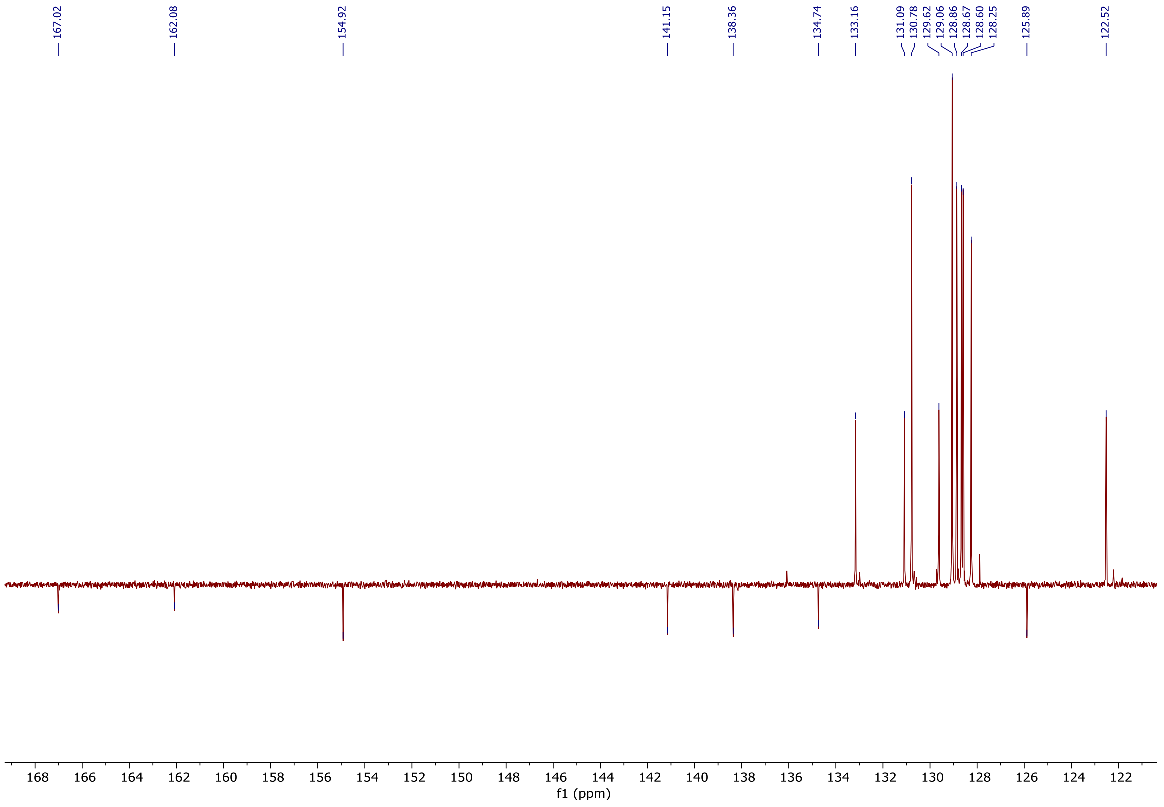

Figure S43. ^{13}C {^{1}H} (APT) NMR ($CDCl_3$, 75.5 MHz) of **1h**

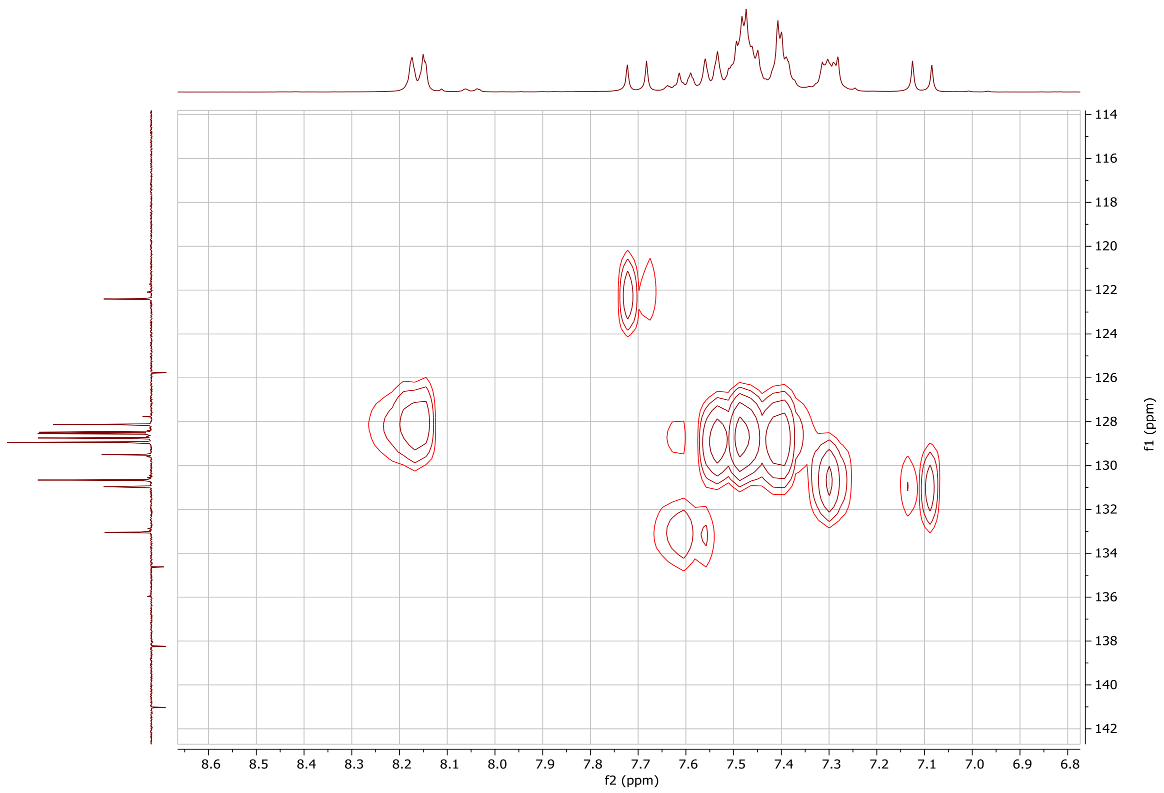

Figure S44. ^{1}H-^{13}C HSQC correlation ($CDCl_3$) of **1h**

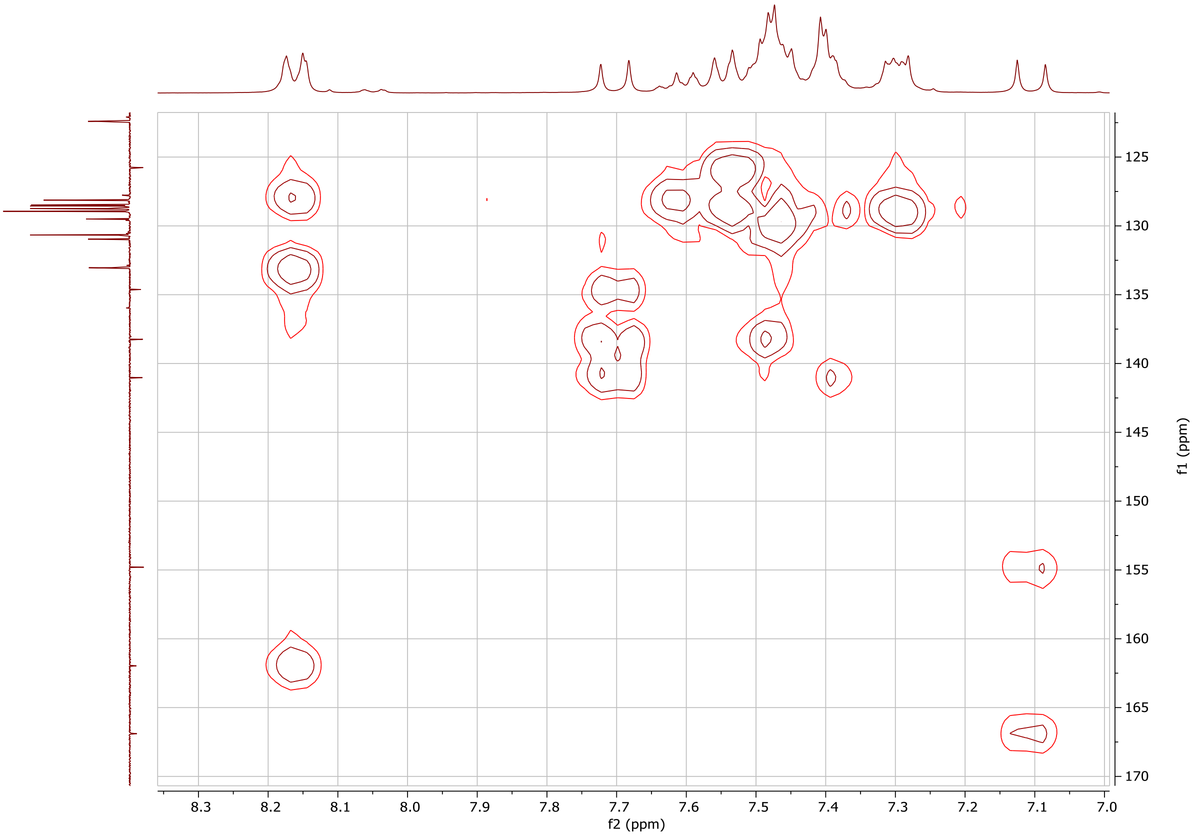

Figure S45. ^{1}H-^{13}C HMBC correlation ($CDCl_3$) of **1h**

1.2.- NMR spectra of the cyclobutane-bis(oxazolone) intermediates 2a-2e.

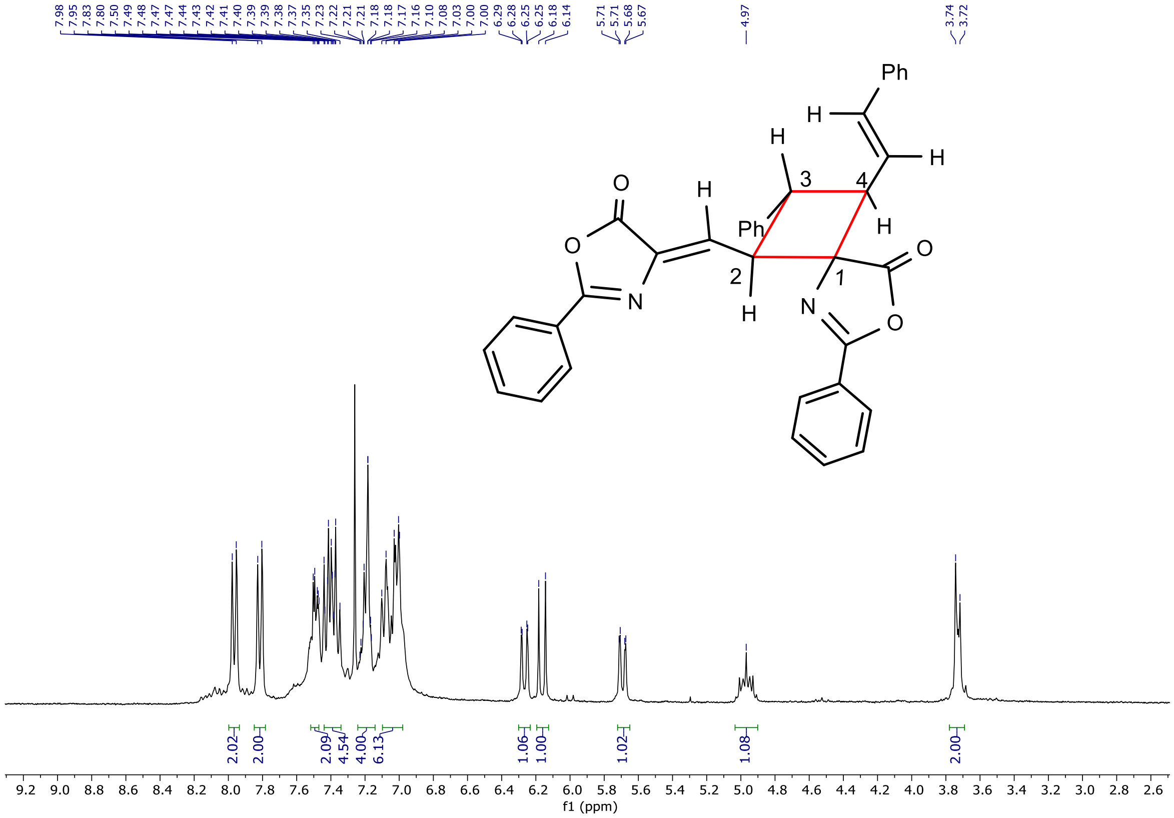

Figure S46. ^{1}H NMR ($CDCl_3$, 300.13 MHz) of **2a**

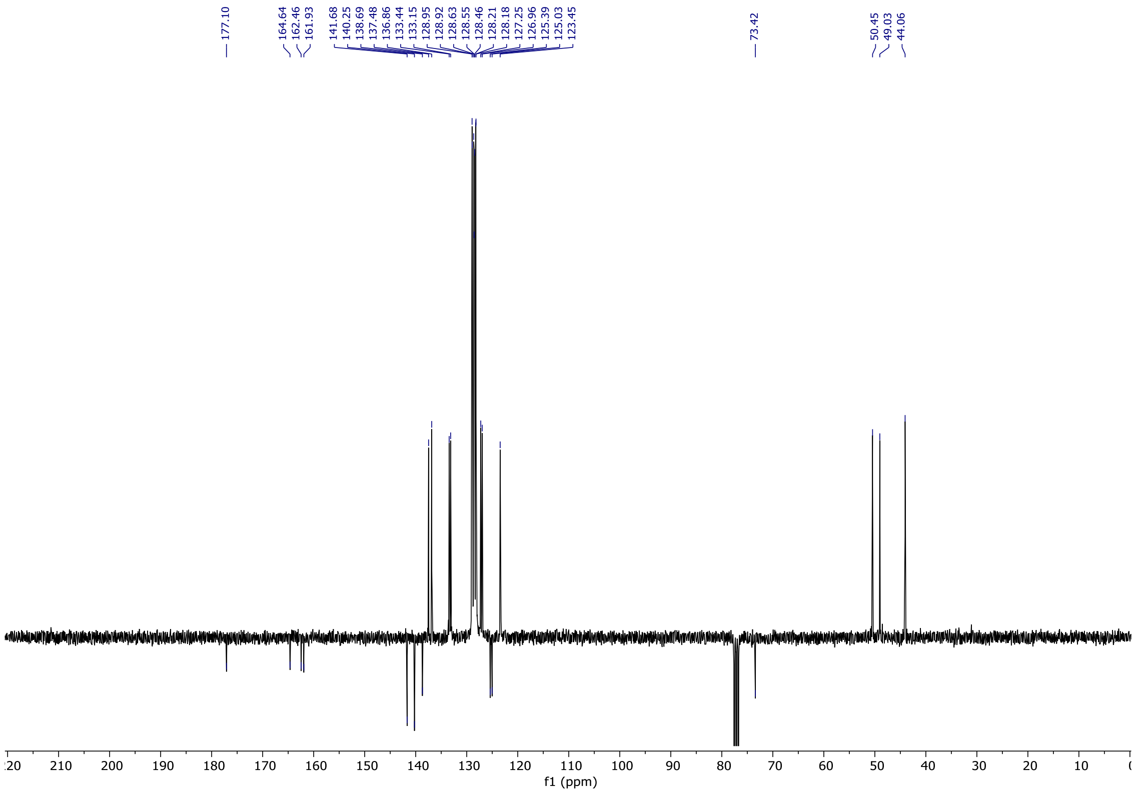

Figure S47. $^{13}C\{^{1}H\}$ (APT) NMR ($CDCl_3$, 75.5 MHz) of **2a**

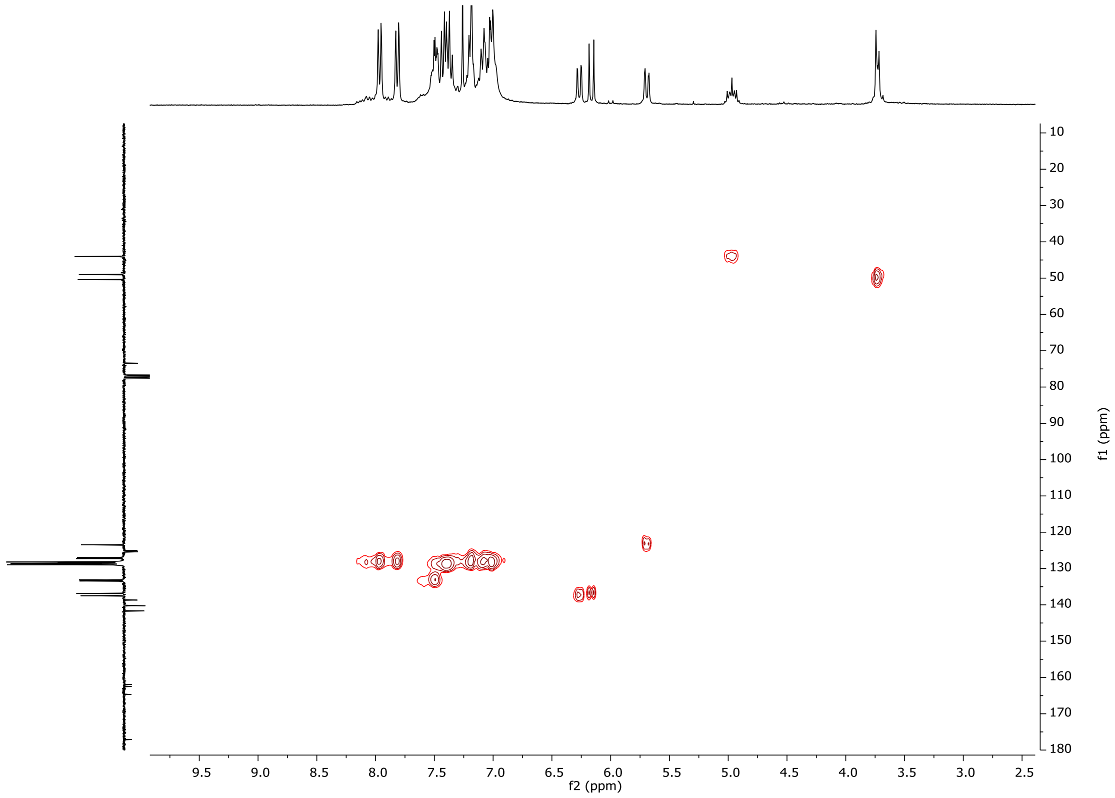

Figure S48. ^{1}H-^{13}C HSQC correlation ($CDCl_3$) of **2a**

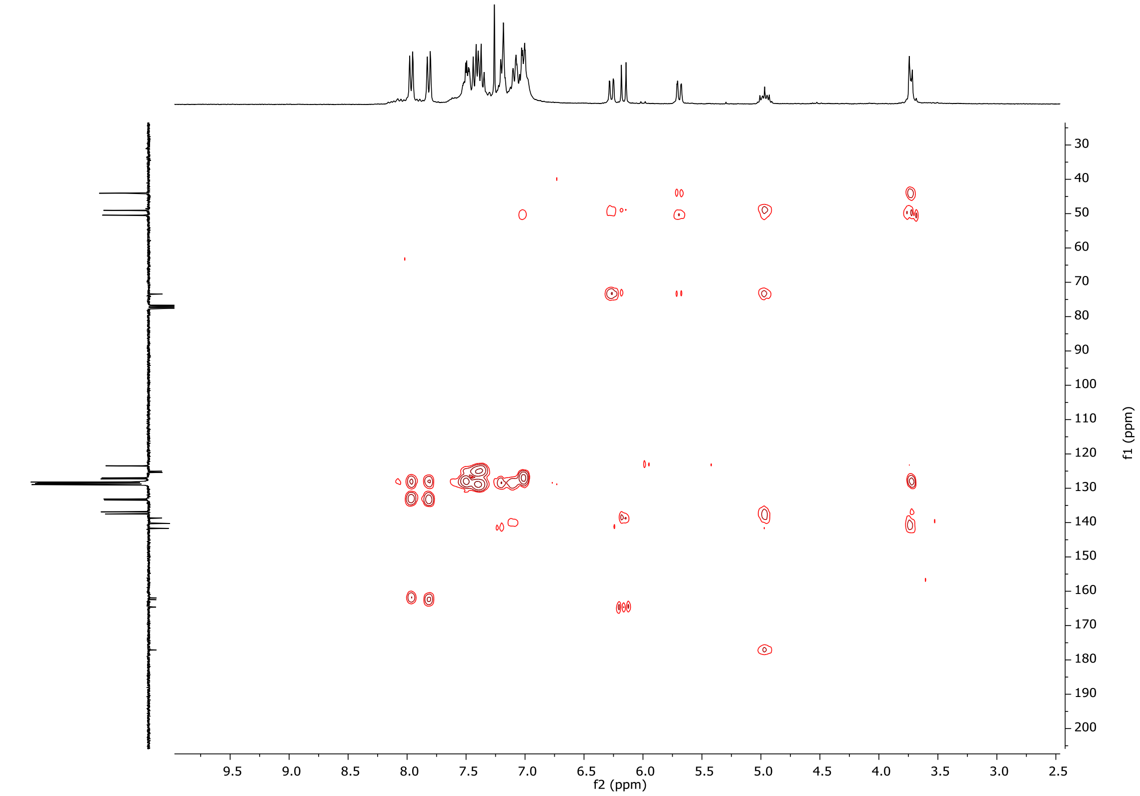

Figure S49. ^{1}H-^{13}C HMBC correlation ($CDCl_3$) of **2a**

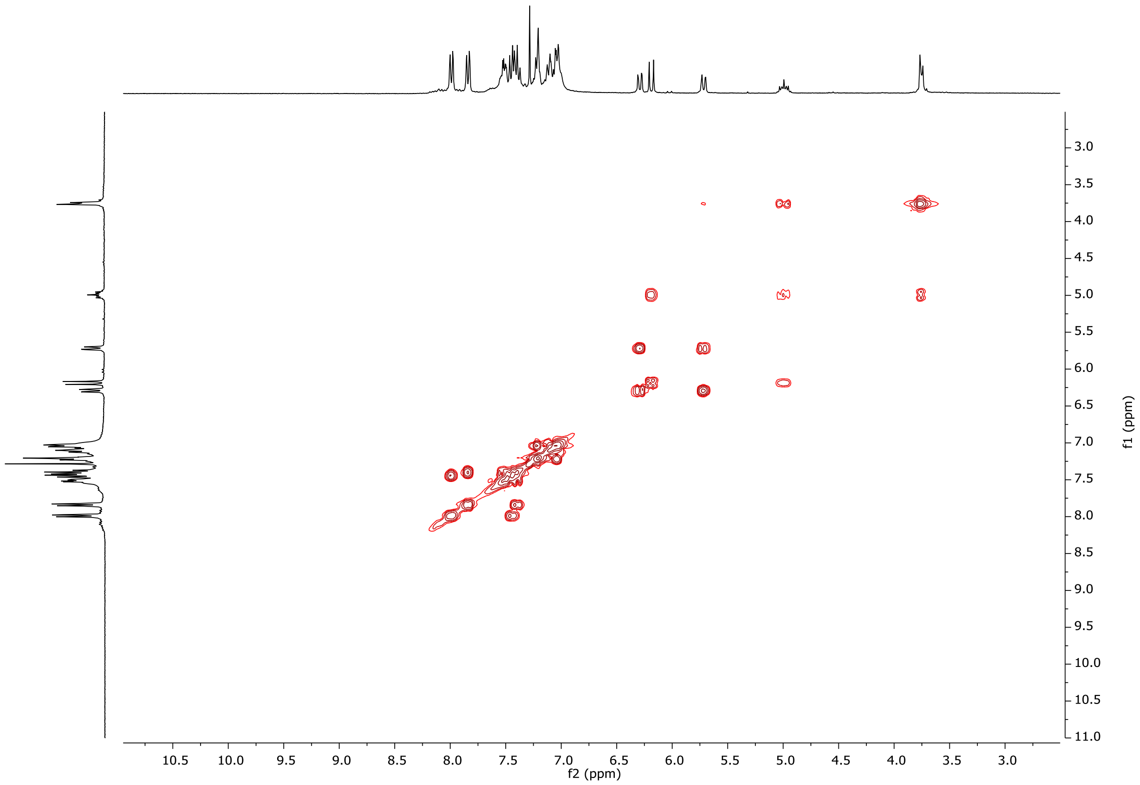

Figure S50. ^{1}H-COSY ($CDCl_3$) of **2a**

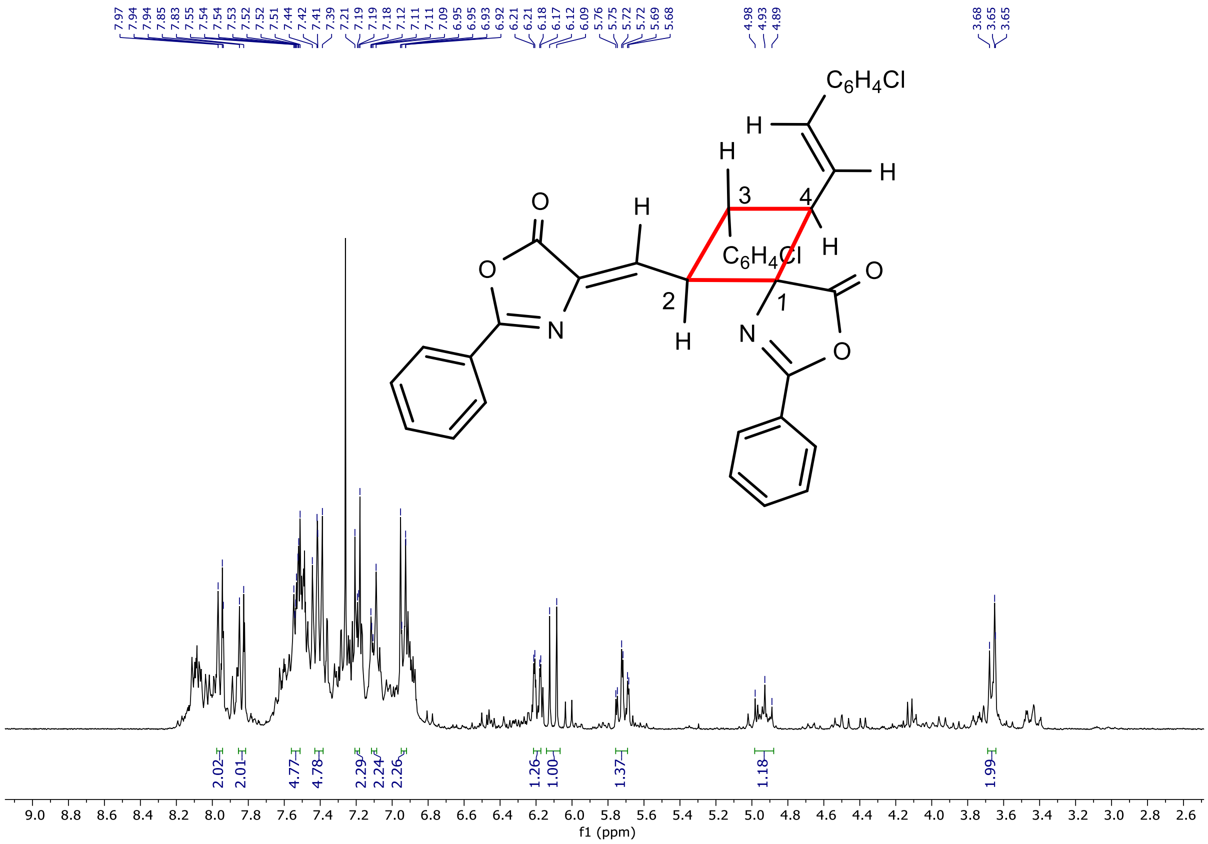

Figure S51. ^{1}H NMR ($CDCl_3$, 300.13 MHz) of **2b**

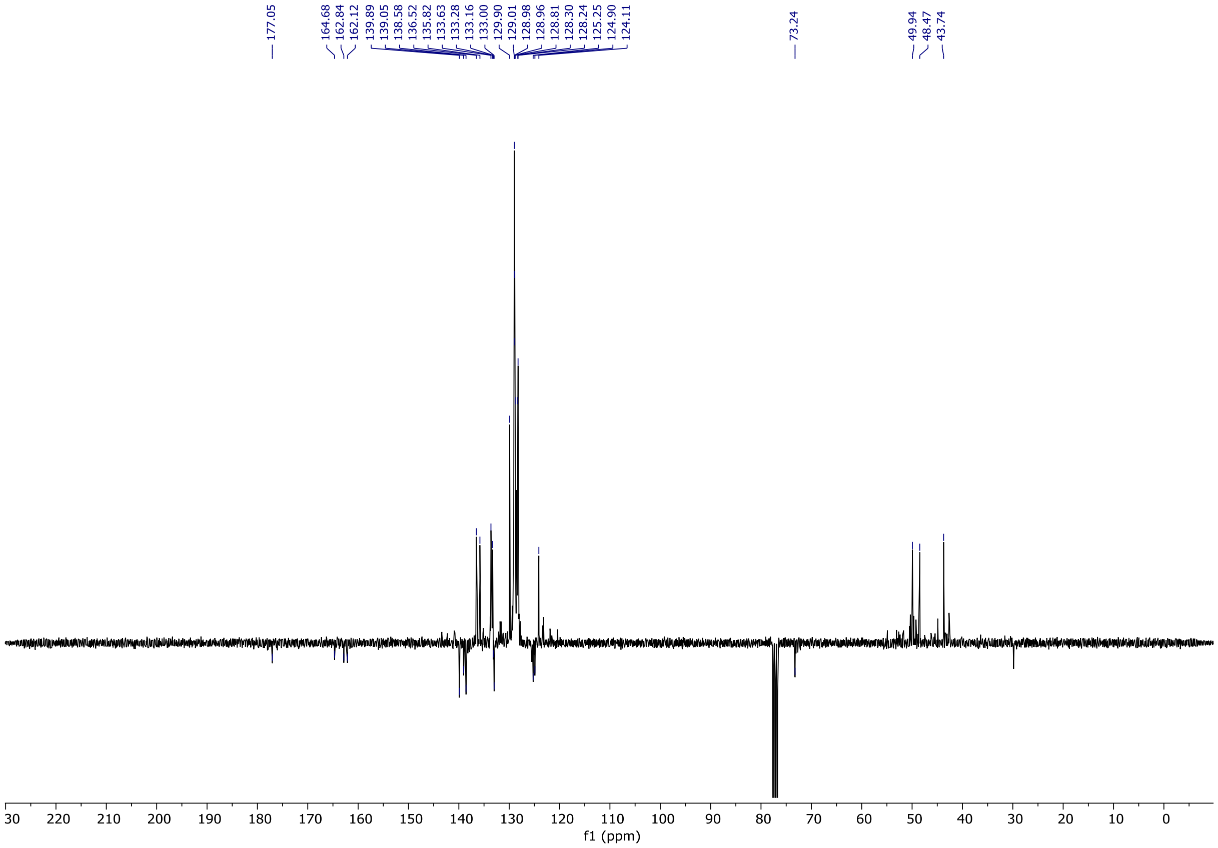

Figure S52. $^{13}C\{^{1}H\}$ (APT) NMR ($CDCl_3$, 75.5 MHz) of **2b**

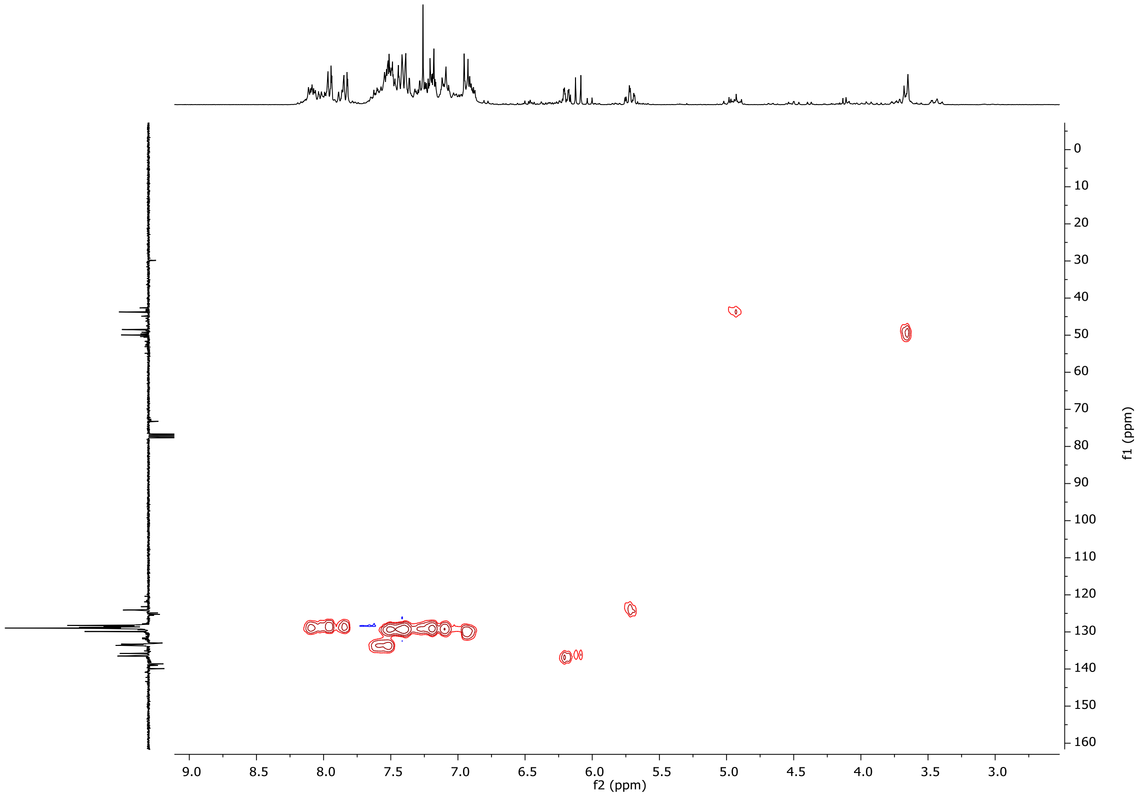

Figure S53. ^{1}H-^{13}C HSQC correlation ($CDCl_3$) of **2b**

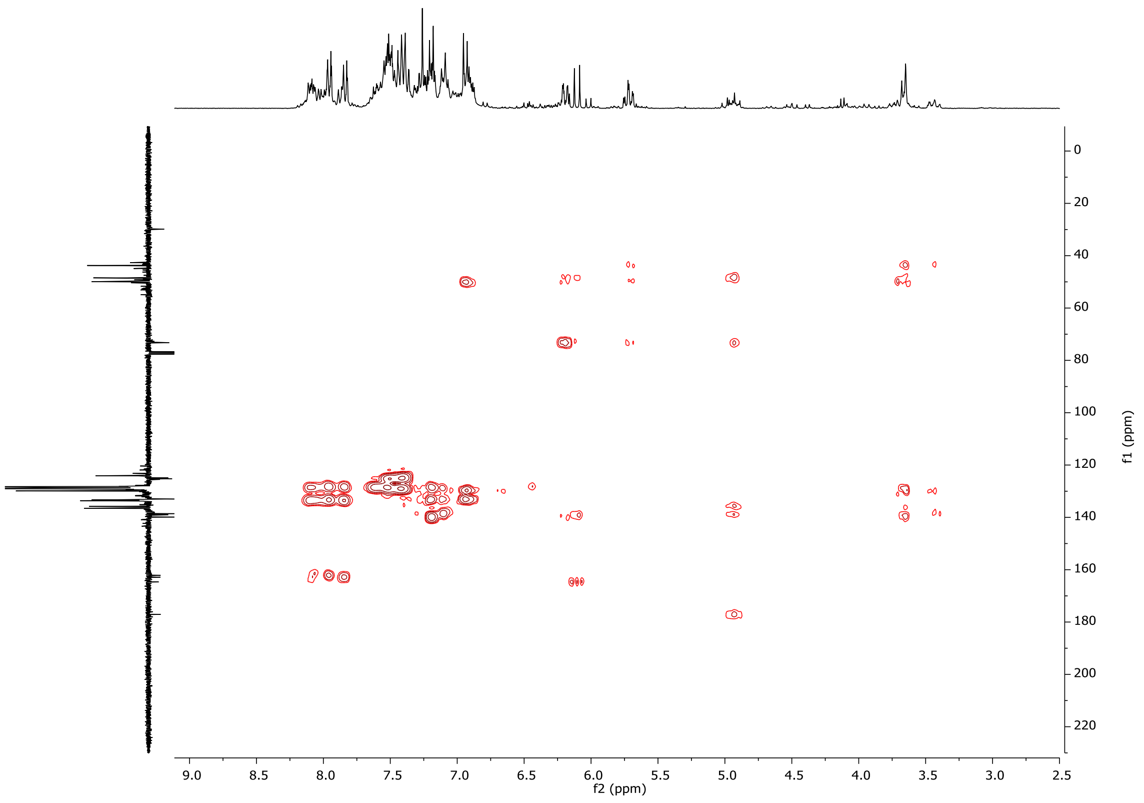

Figure S54. ^{1}H-^{13}C HMBC correlation ($CDCl_3$) of **2b**

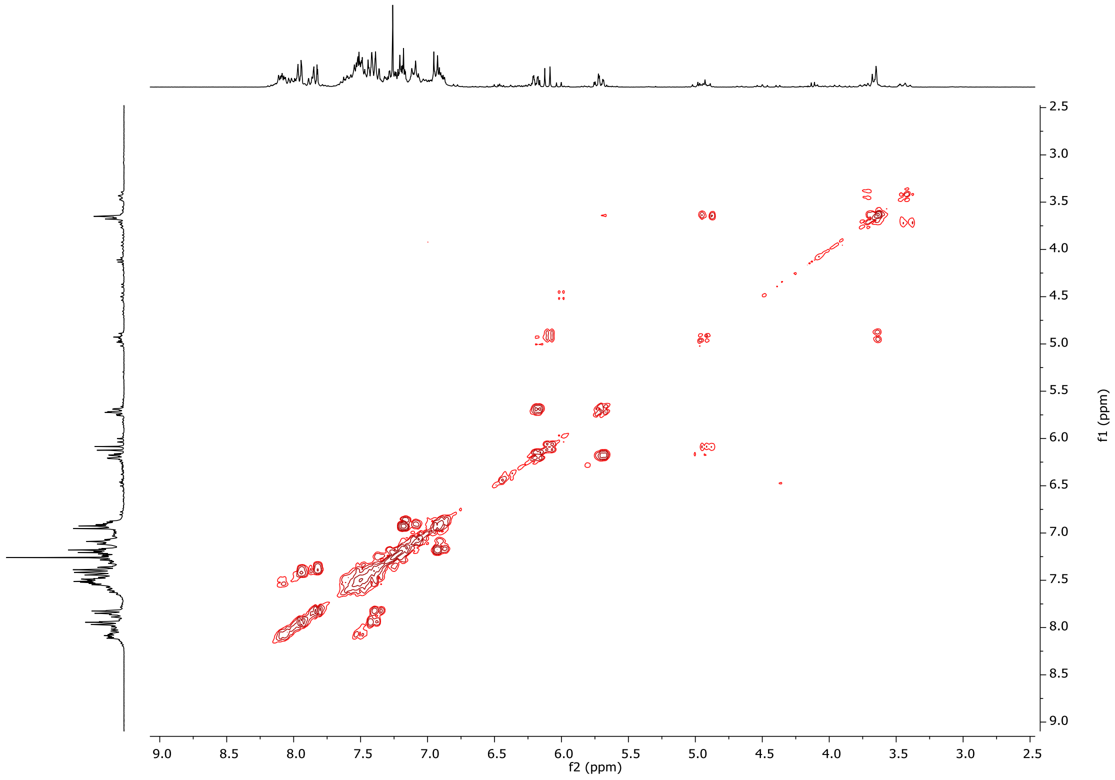

Figure S55. ^1H-COSY ($CDCl_3$) of **2b**

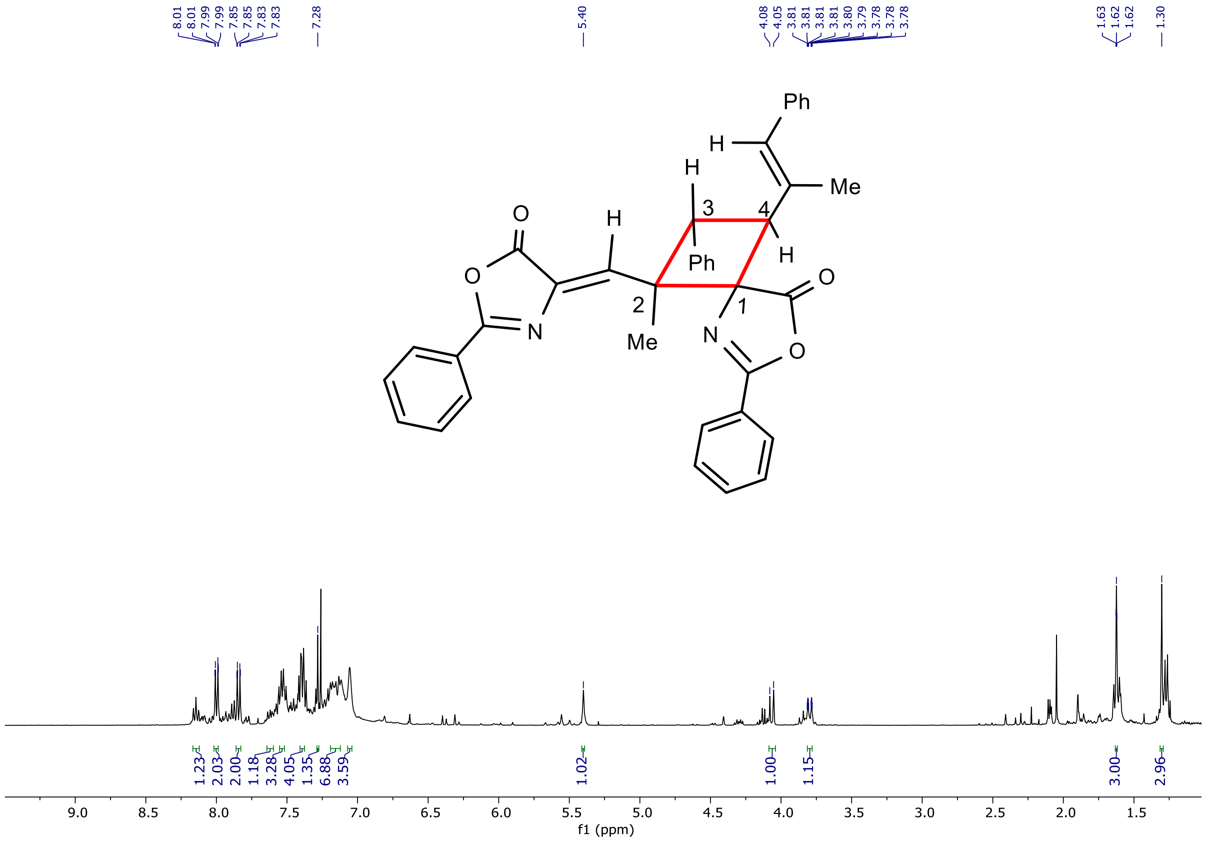

Figure S56. ^1H NMR ($CDCl_3$, 300.13 MHz) of **2d**

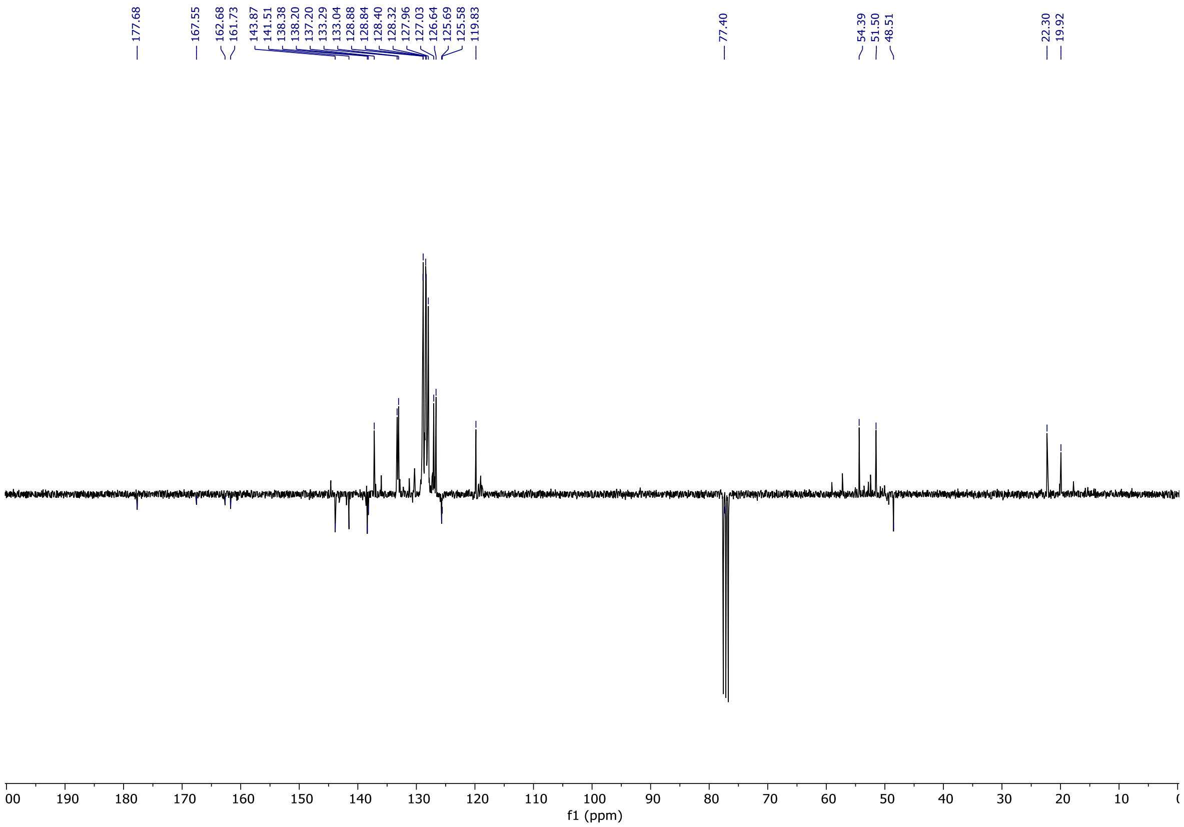

Figure S57. $^{13}C\{^{1}H\}$ (APT) NMR ($CDCl_3$, 75.5 MHz) of **2d**

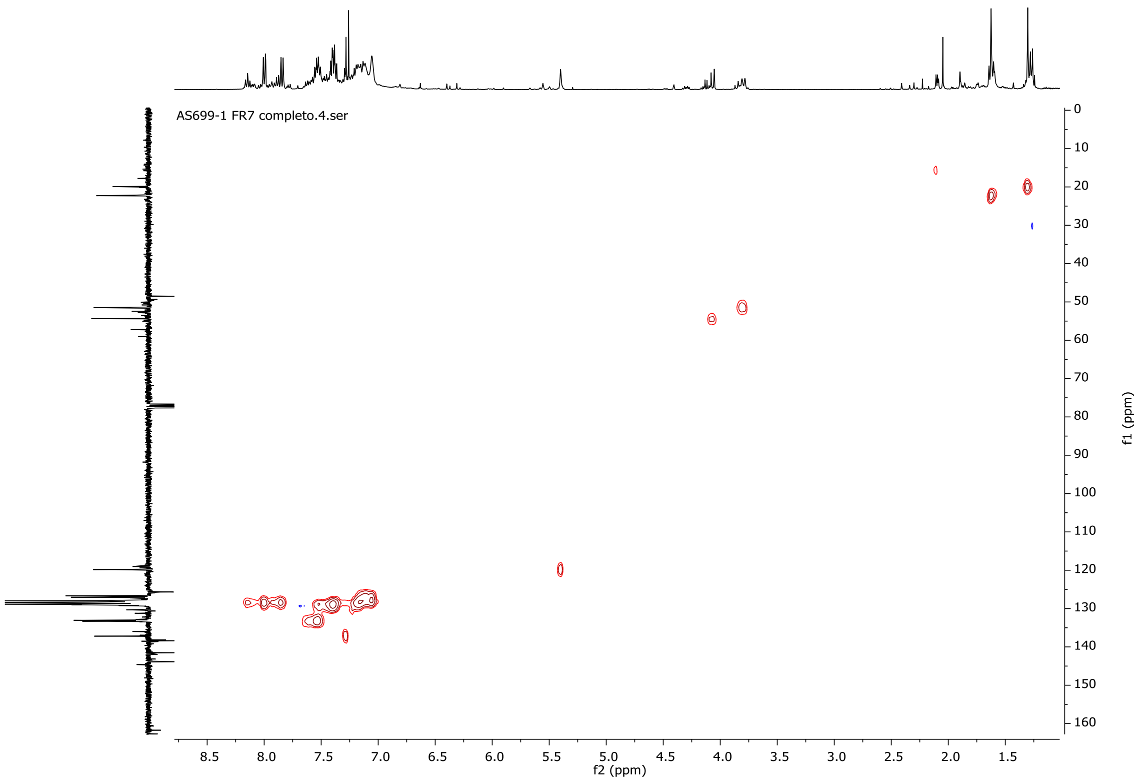

Figure S58. ^{1}H-^{13}C HSQC correlation ($CDCl_3$) of **2d**

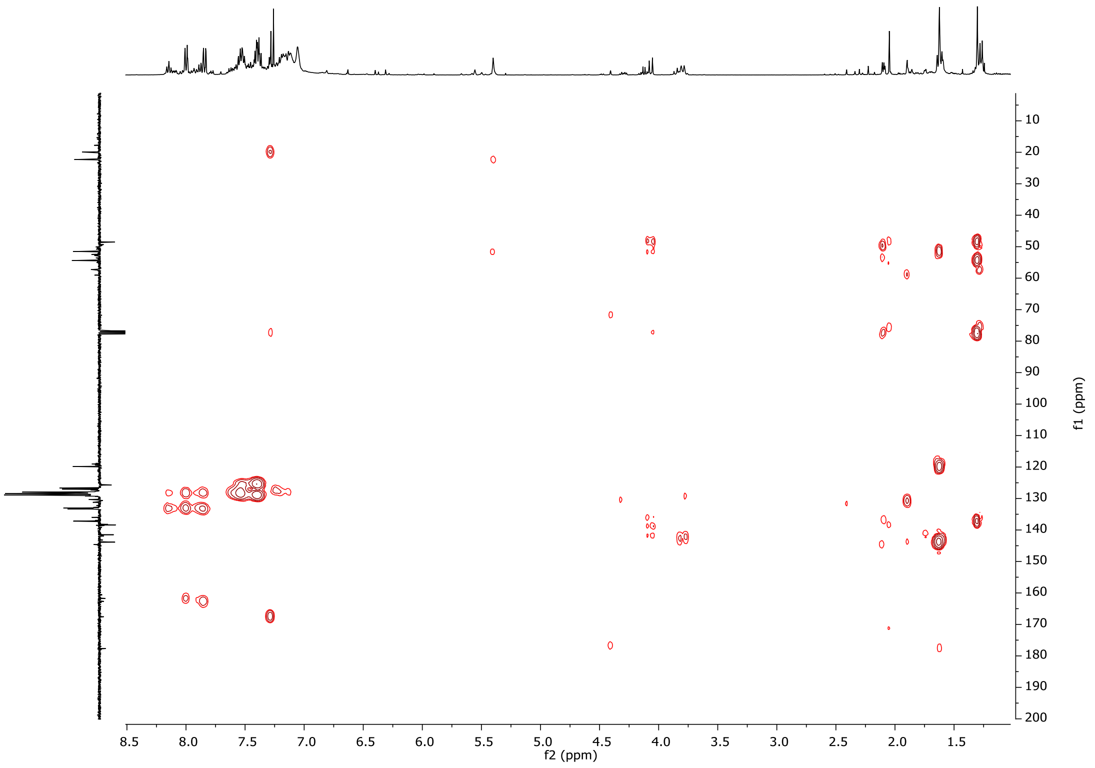

Figure S59. ^{1}H-^{13}C HMBC correlation ($CDCl_3$) of **2d**

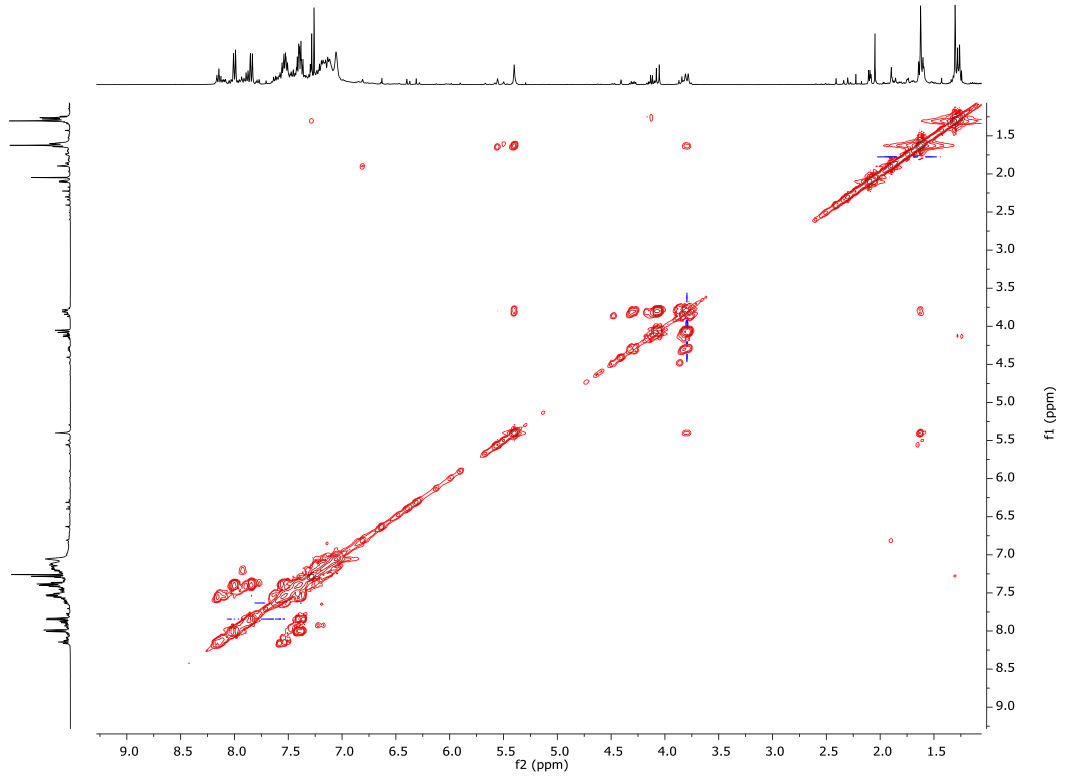

Figure S60. ^{1}H-COSY ($CDCl_3$) of **2d**

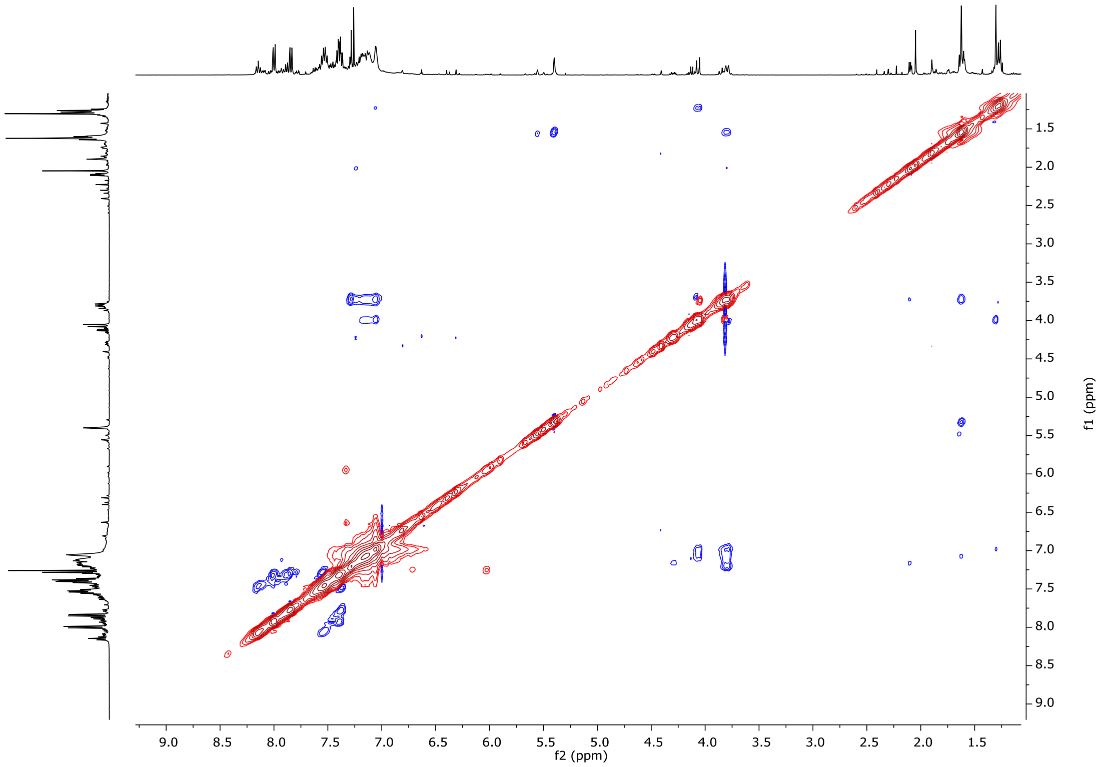

Figure S61. ^{1}H-NOESY ($CDCl_3$) of **2d**

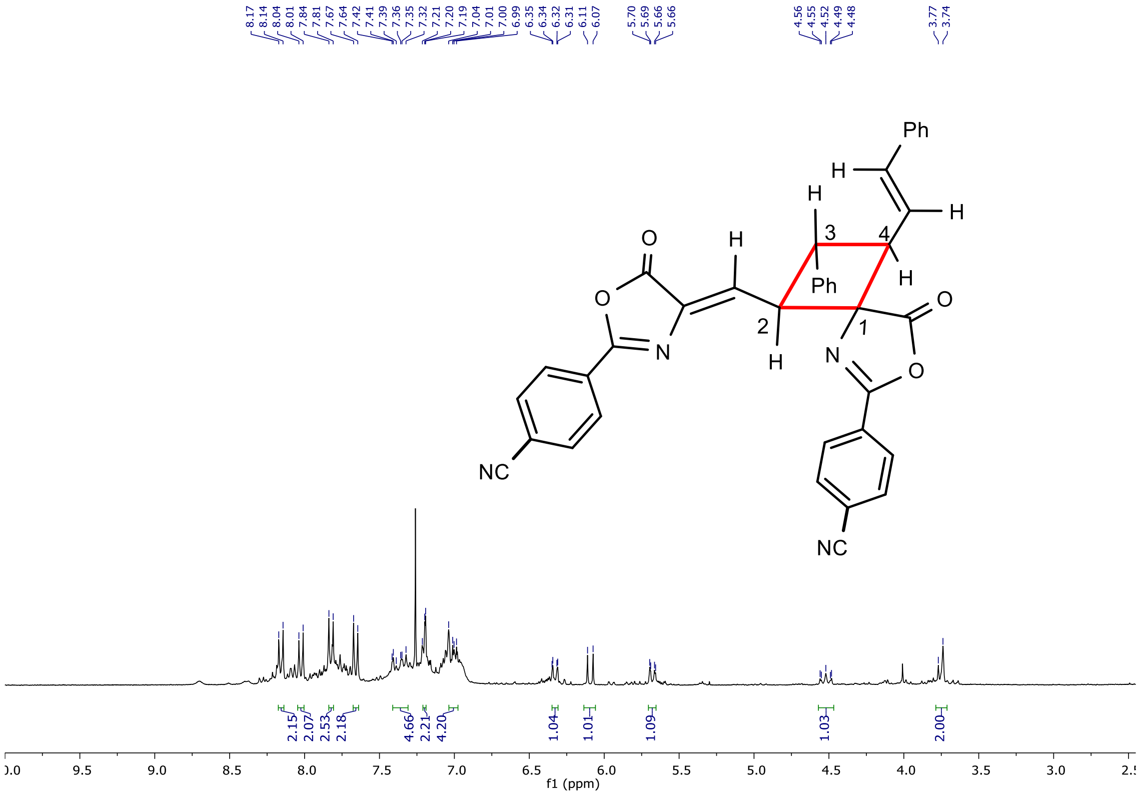

Figure S62. ^{1}H NMR ($CDCl_3$, 300.13 MHz) of **2f**

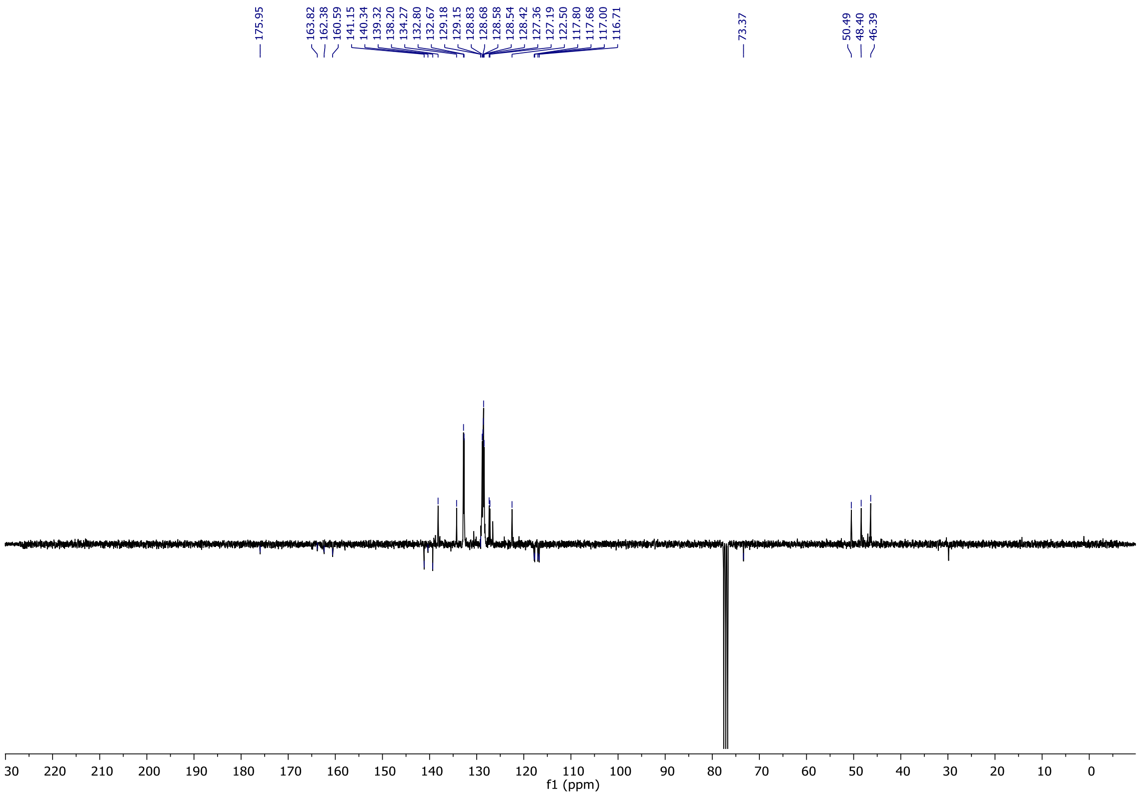

Figure S63. $^{13}C\{^{1}H\}$ (APT) NMR ($CDCl_3$, 75.5 MHz) of **2f**

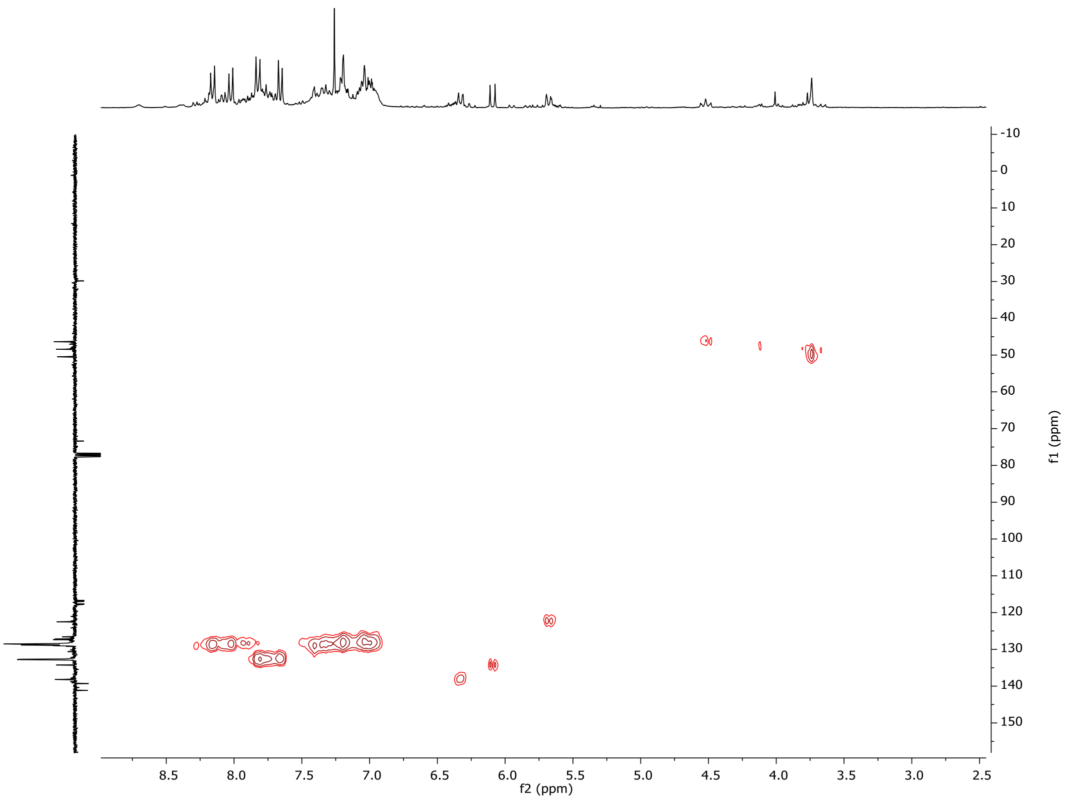

Figure S64. ^{1}H-^{13}C HSQC correlation ($CDCl_3$) of **2f**

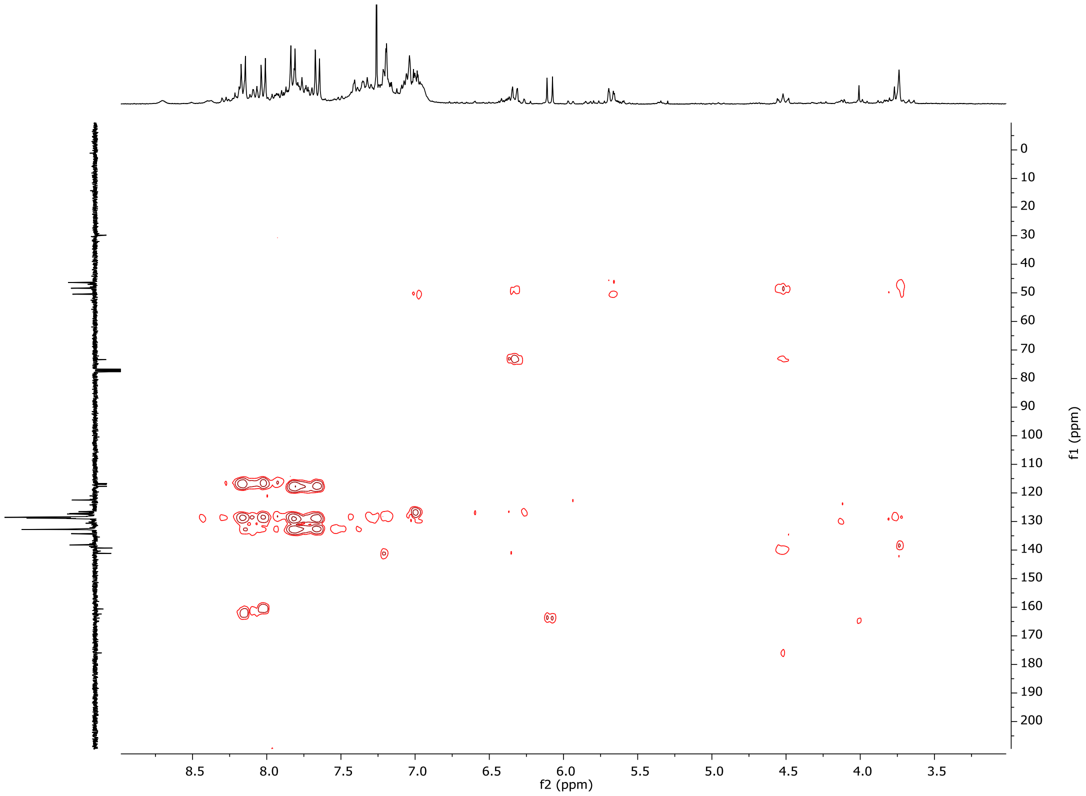

Figure S65. ^{1}H-^{13}C HMBC correlation ($CDCl_3$) of **2f**

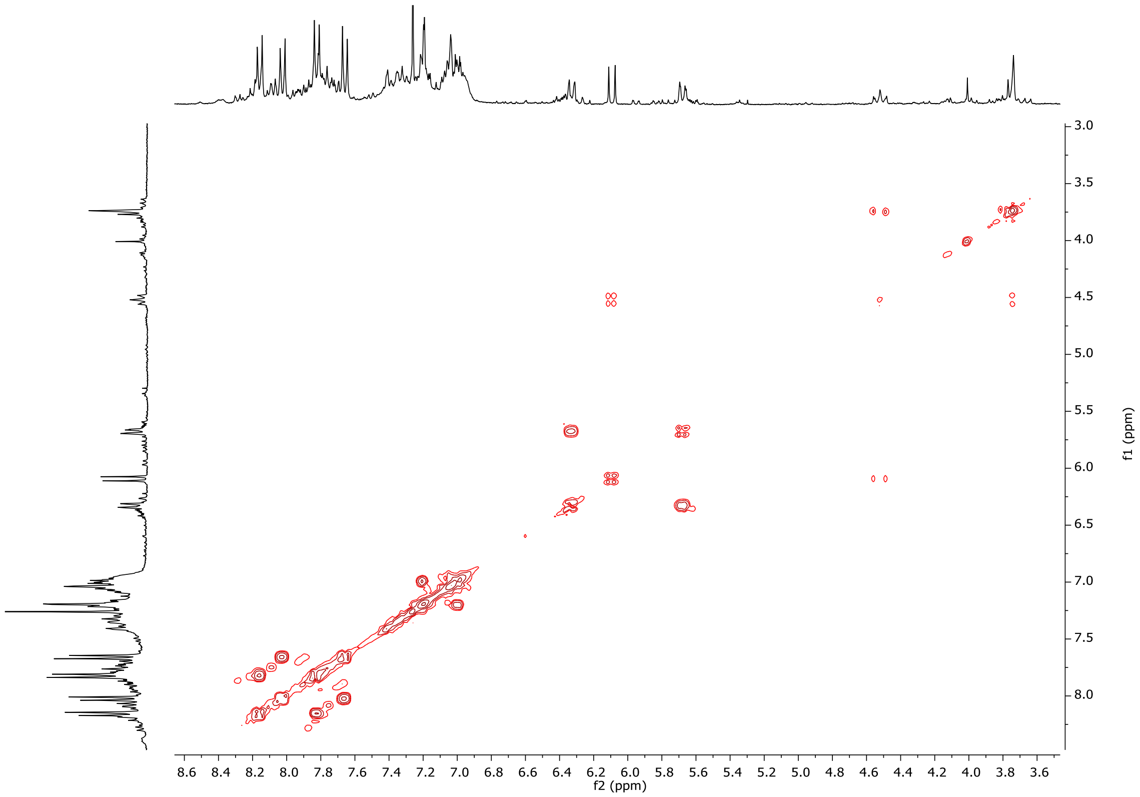

Figure S66. ^{1}H-COSY ($CDCl_3$) of **2f**

1.3.- NMR spectra of the 1,2-diaminotruxinic bis(amino acids) 3a-3b.

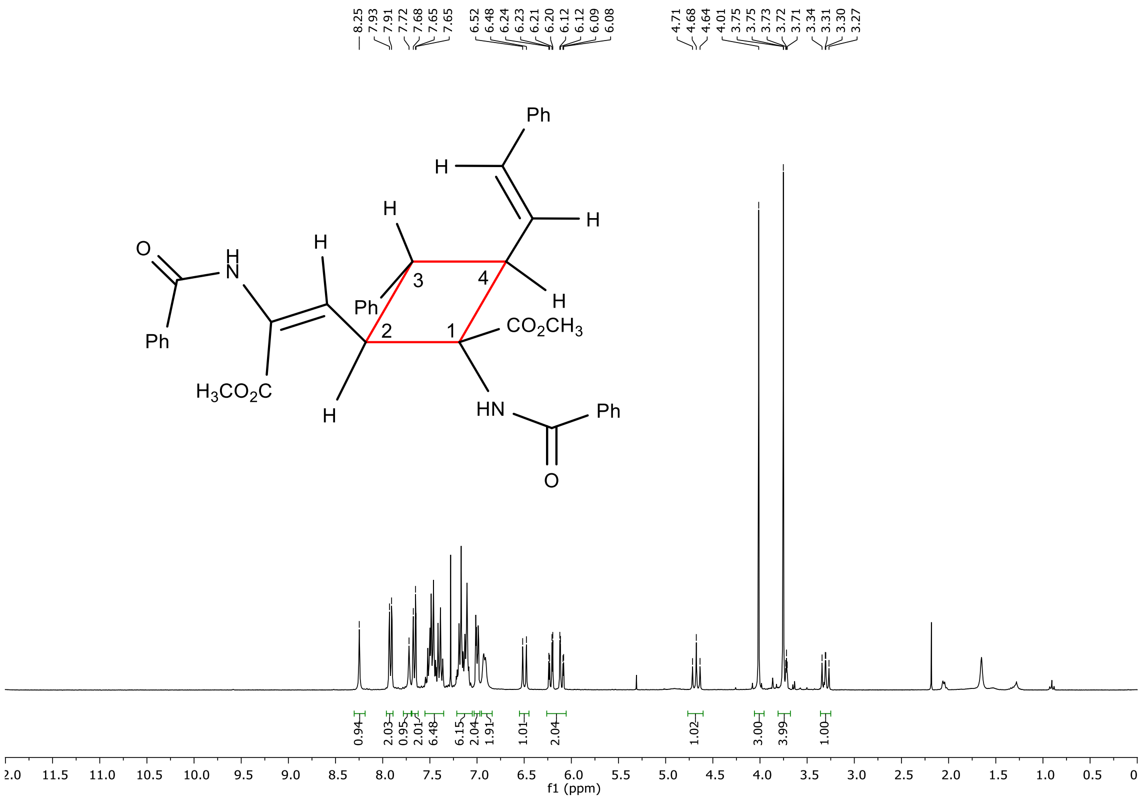

Figure S67. ^{1}H NMR ($CDCl_3$, 500.13 MHz) of **3a**

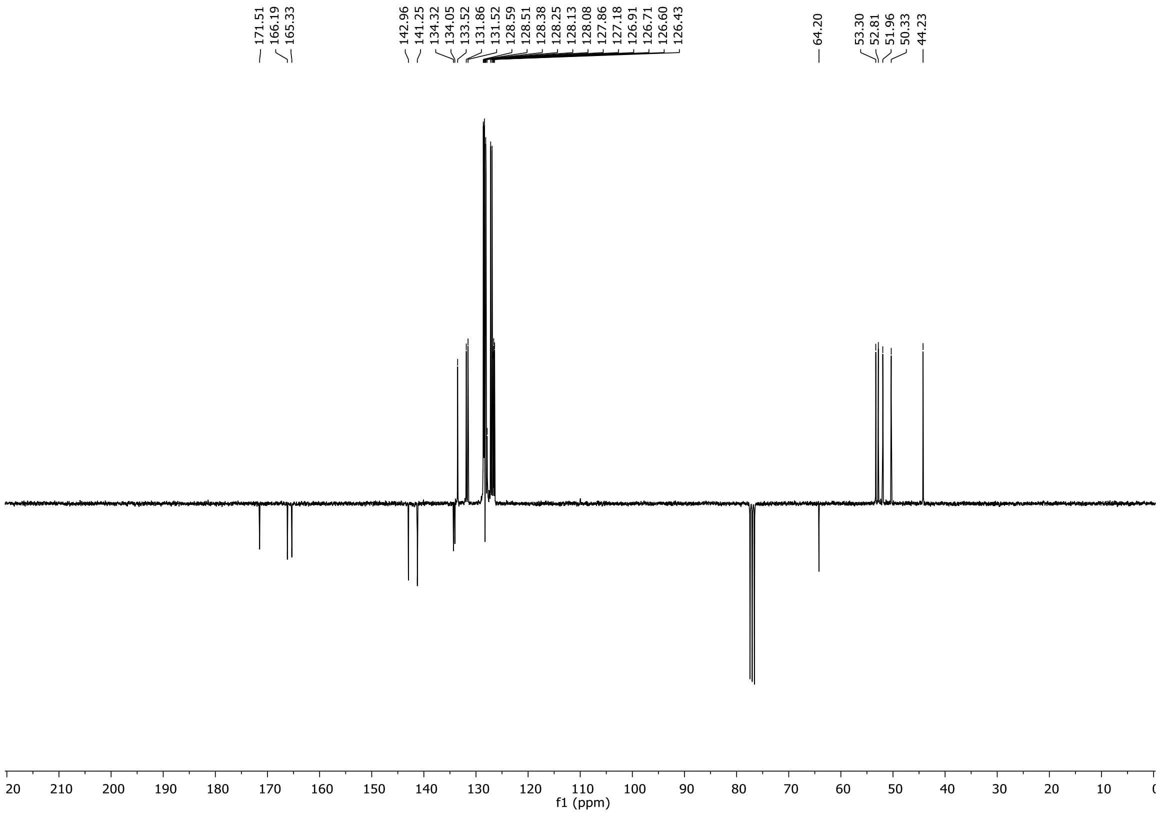

Figure S68. ^{13}C {^{1}H} (APT) NMR ($CDCl_3$, 125.7 MHz) of **3a**

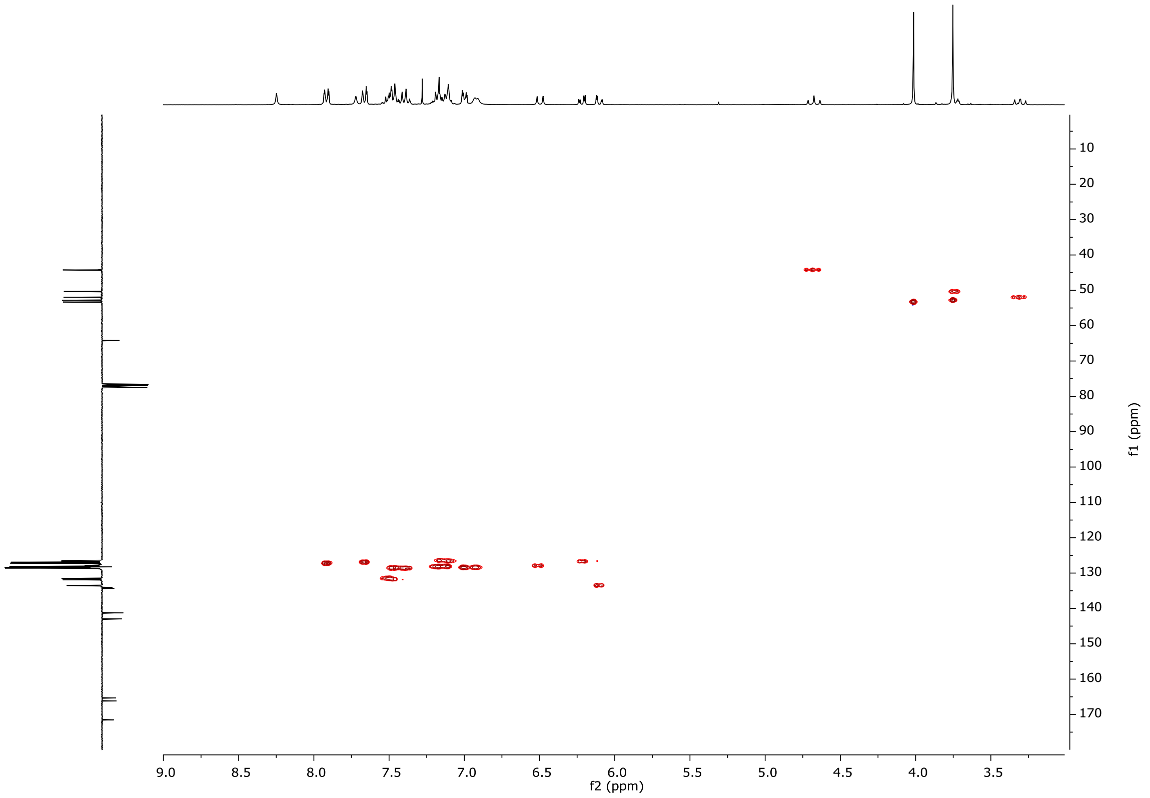

Figure S69. ^{1}H-^{13}C HSQC correlation ($CDCl_3$) of **3a**

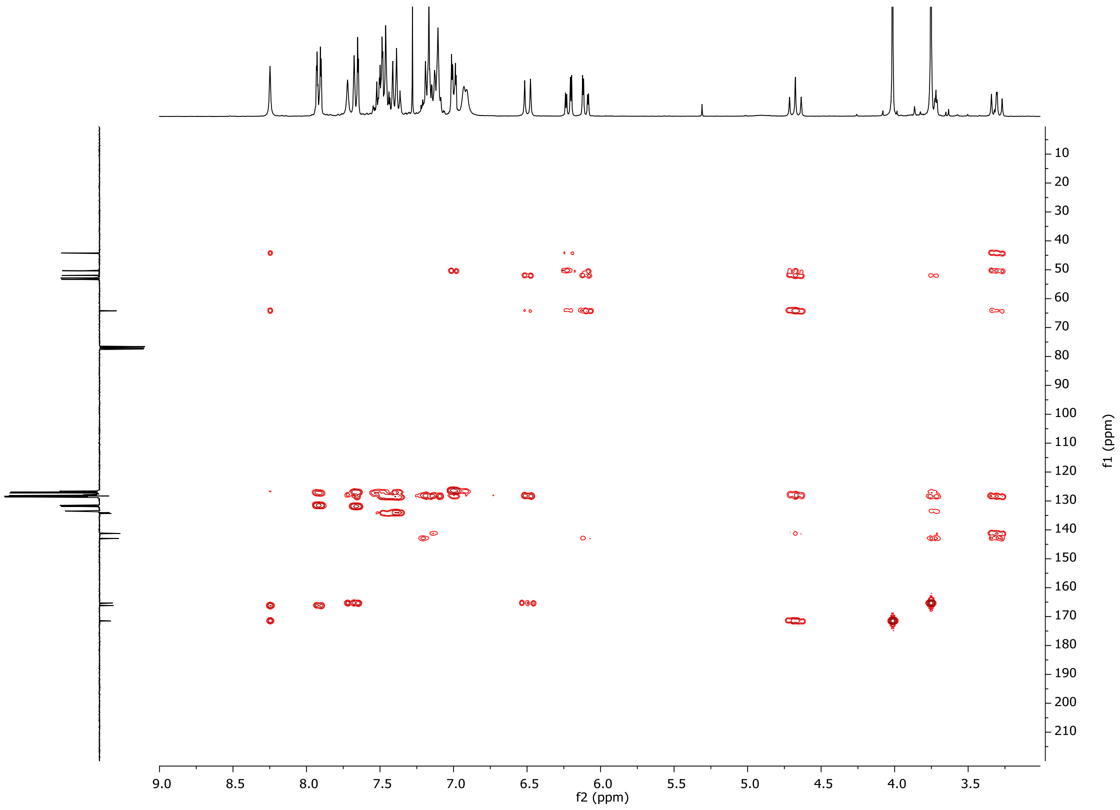

Figure S70. ^{1}H-^{13}C HMBC correlation ($CDCl_3$) of **3a**

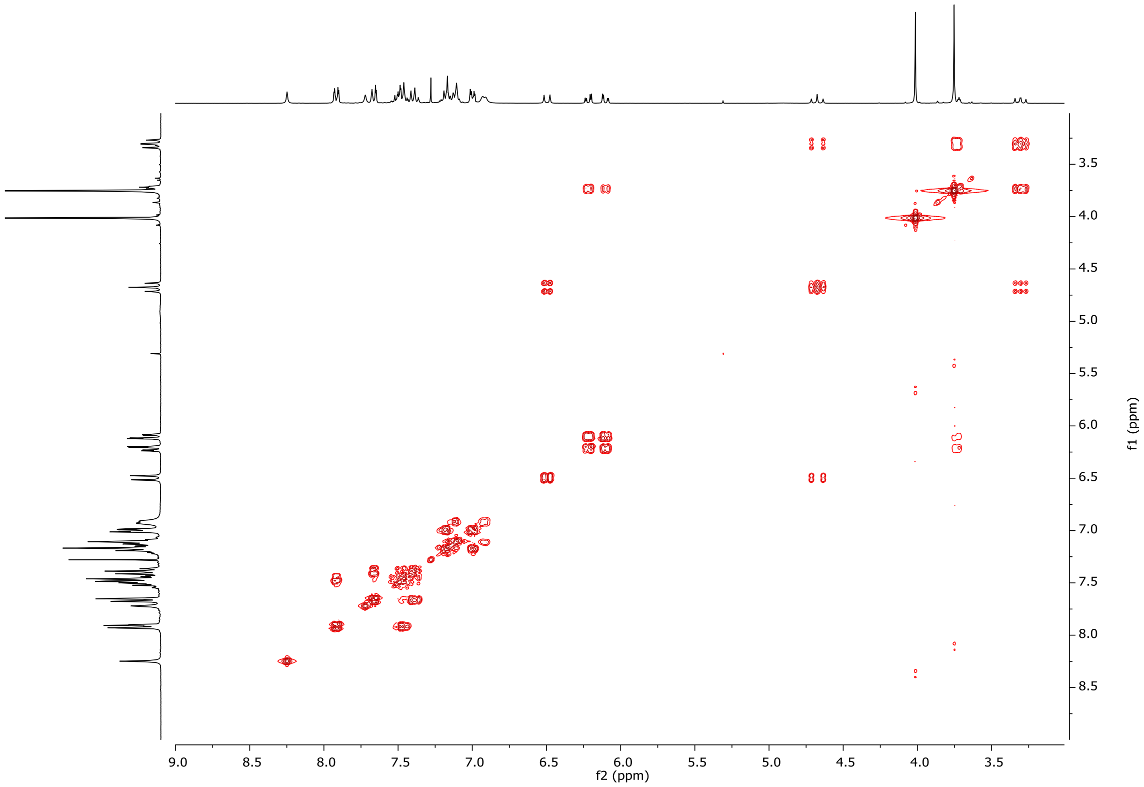

Figure S71. ^{1}H-^{1}H COSY ($CDCl_3$) of **3a**

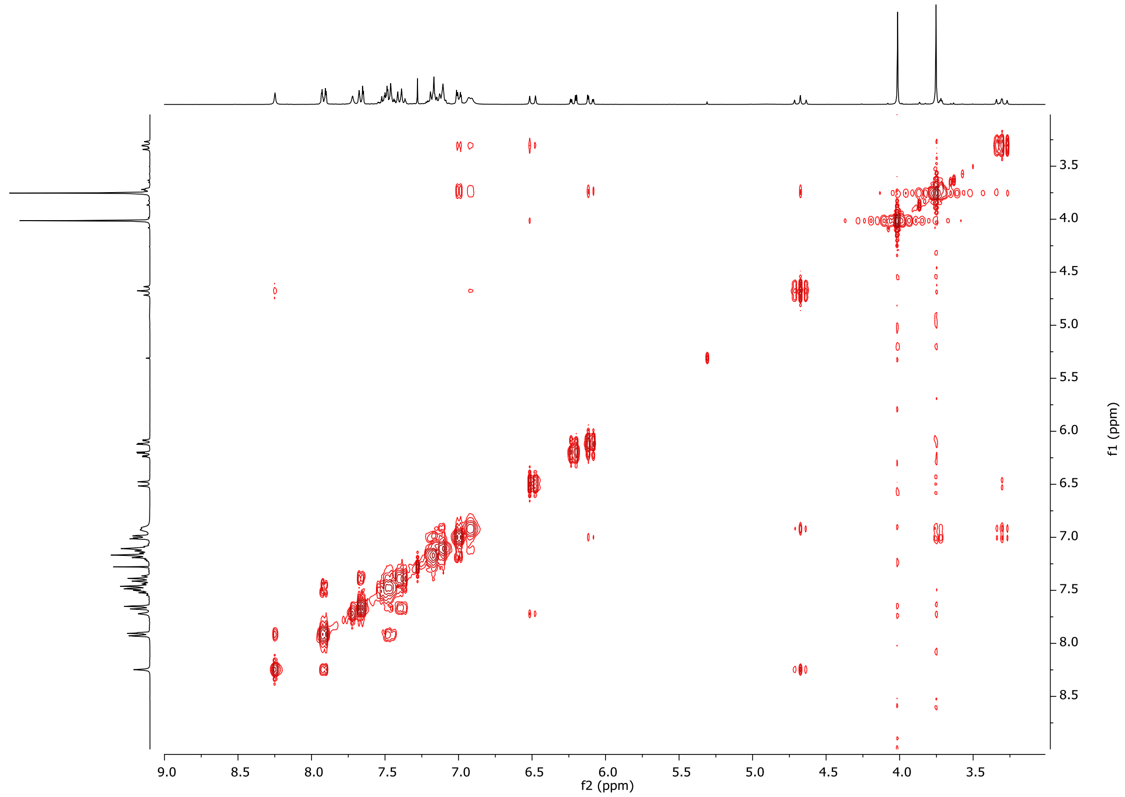

Figure S72. ^{1}H-^{1}H NOESY ($CDCl_3$) of **3a**

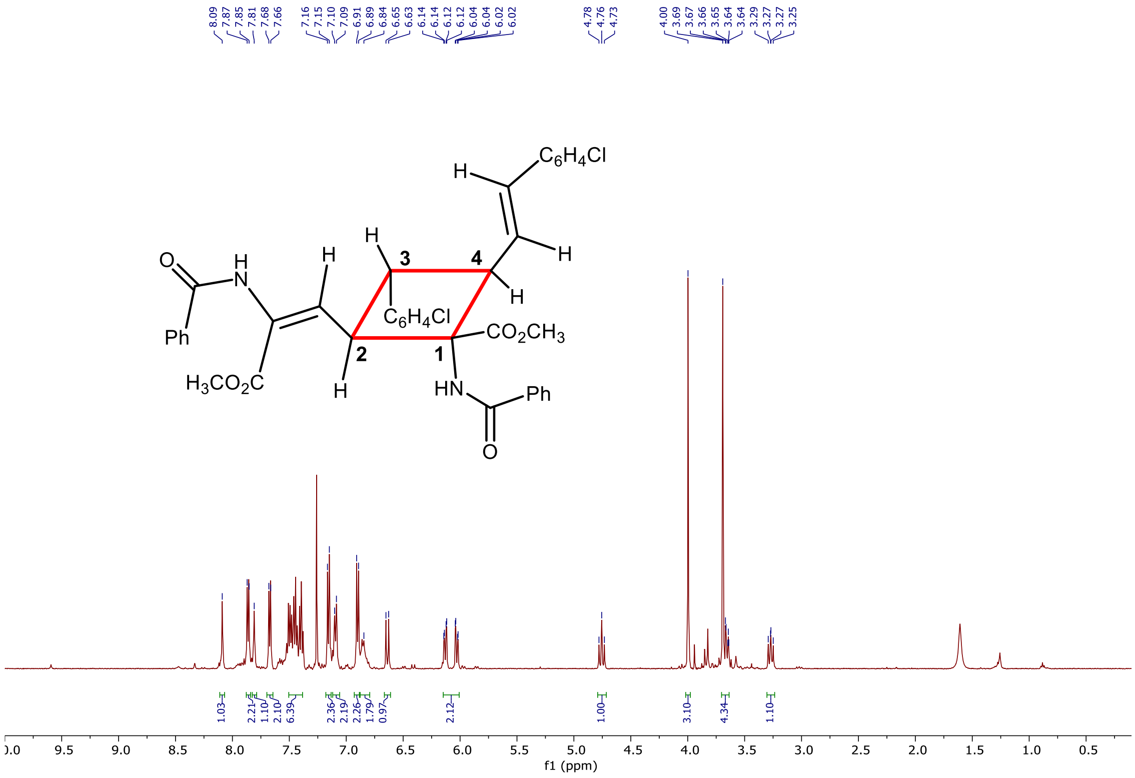

Figure S73. ^{1}H NMR ($CDCl_3$, 500.13 MHz) of **3b**

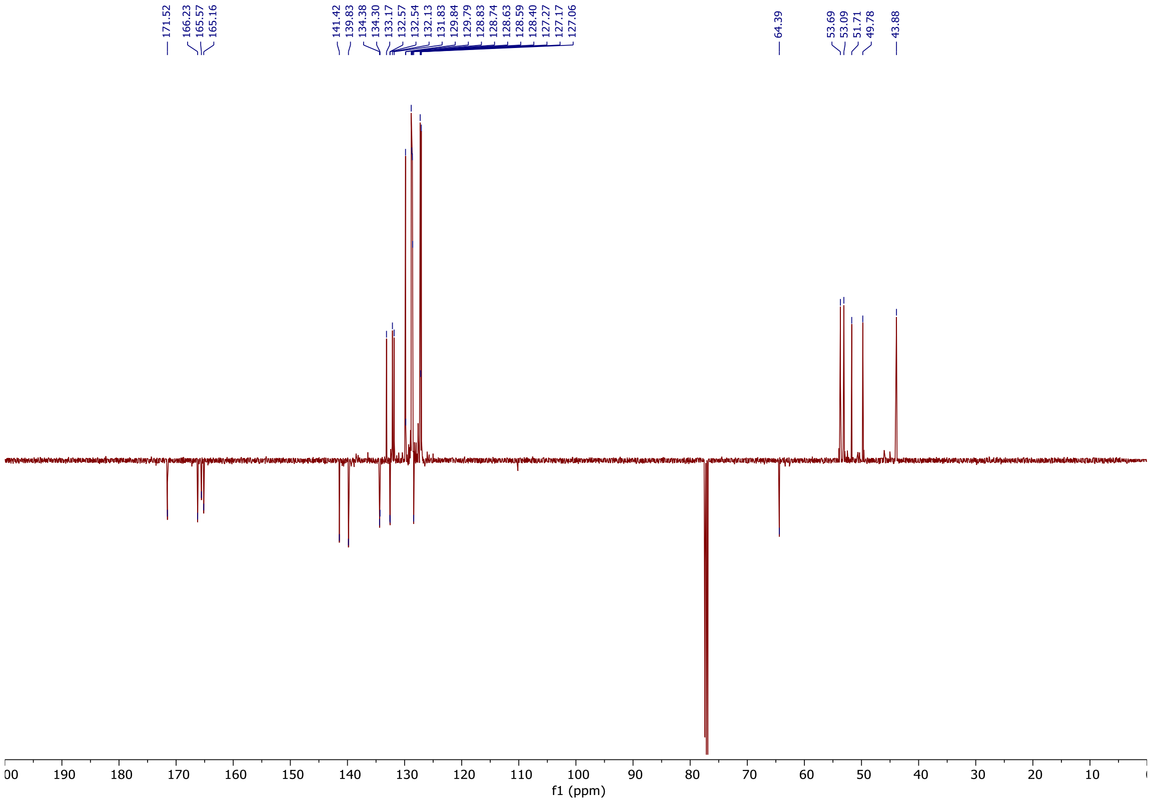

Figure S74. ^{13}C {^{1}H} (APT) NMR ($CDCl_3$, 125.7 MHz) of **3b**

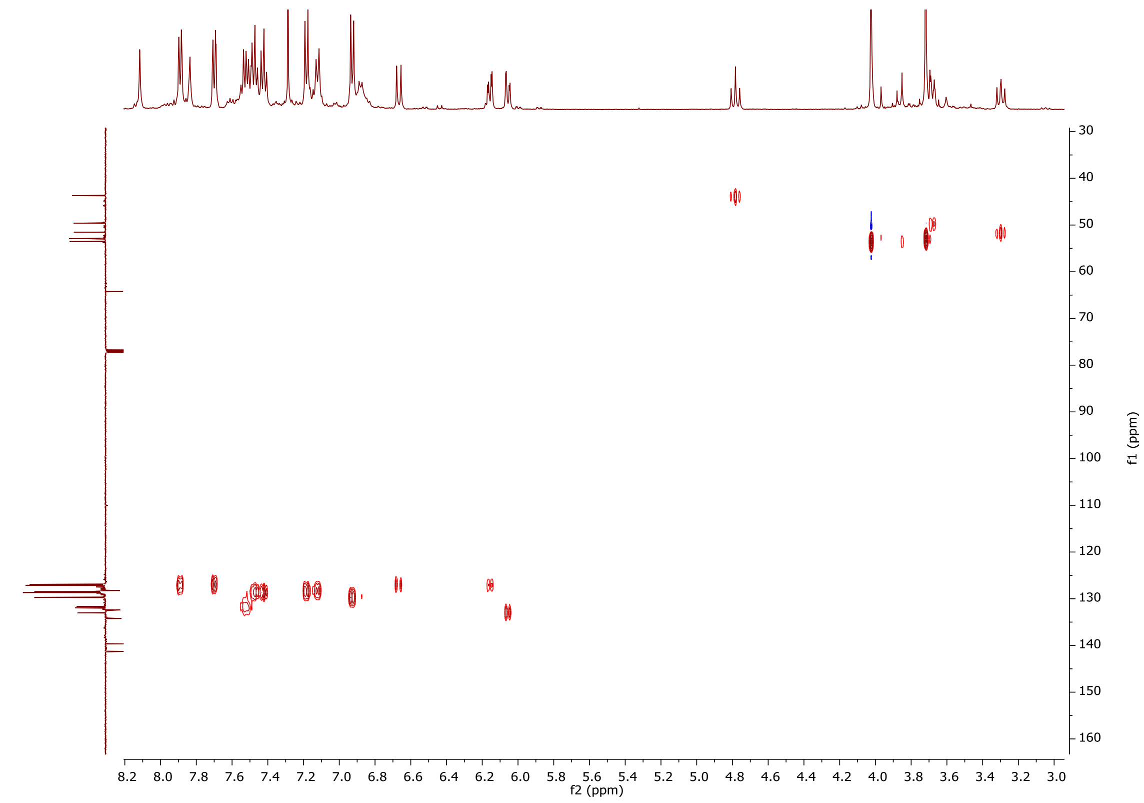

Figure S75. ^{1}H-^{13}C HSQC correlation ($CDCl_3$) of **3b**

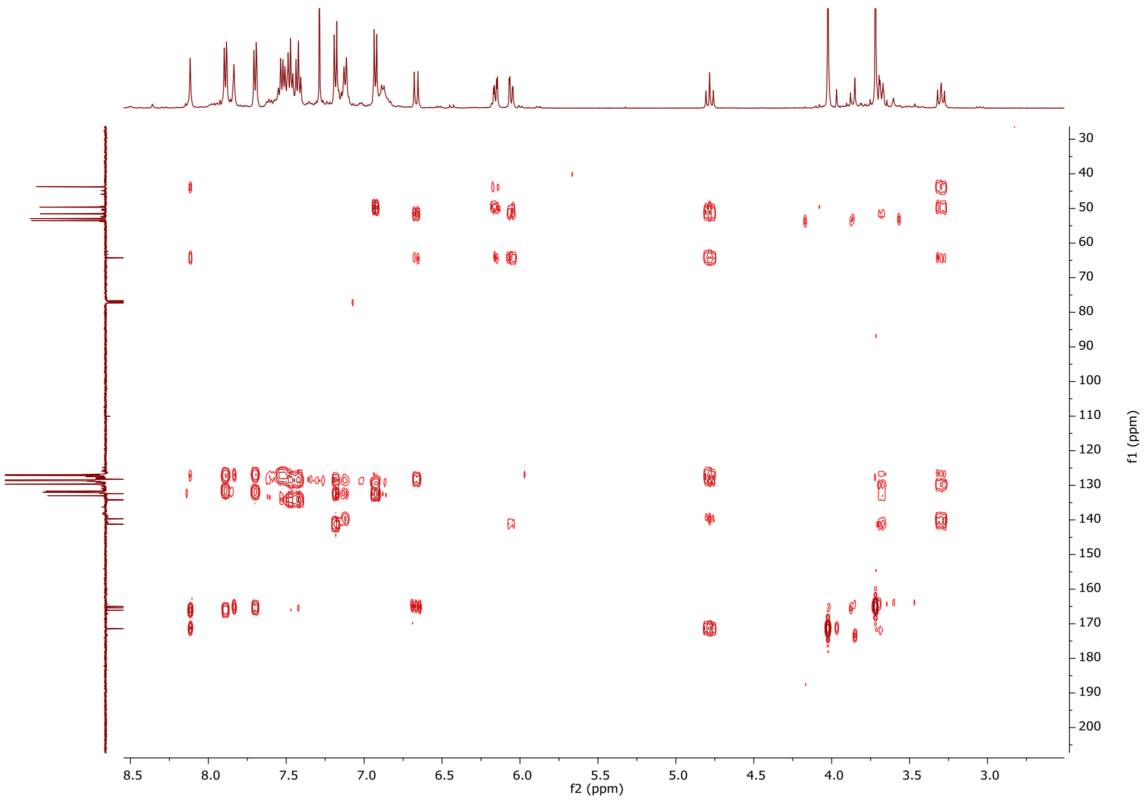

Figure S76. ^{1}H-^{13}C HMBC correlation ($CDCl_3$) of **3b**

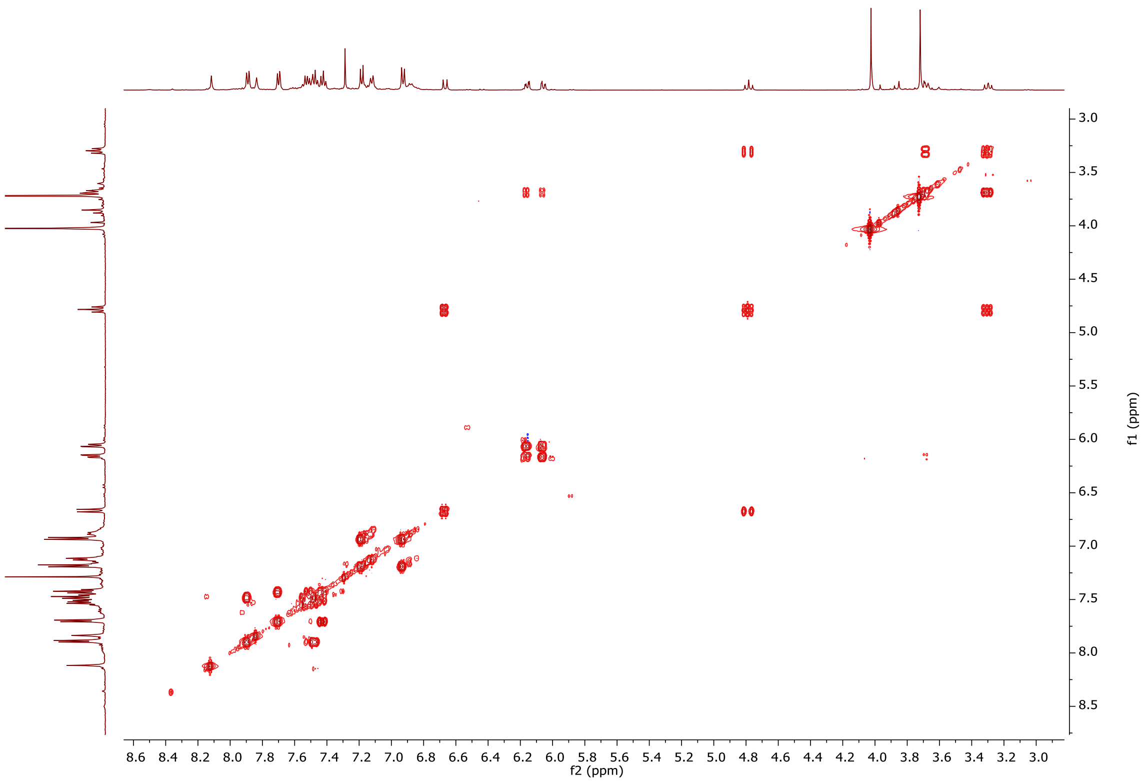

Figure S77. ^{1}H-COSY ($CDCl_3$) of **3b**

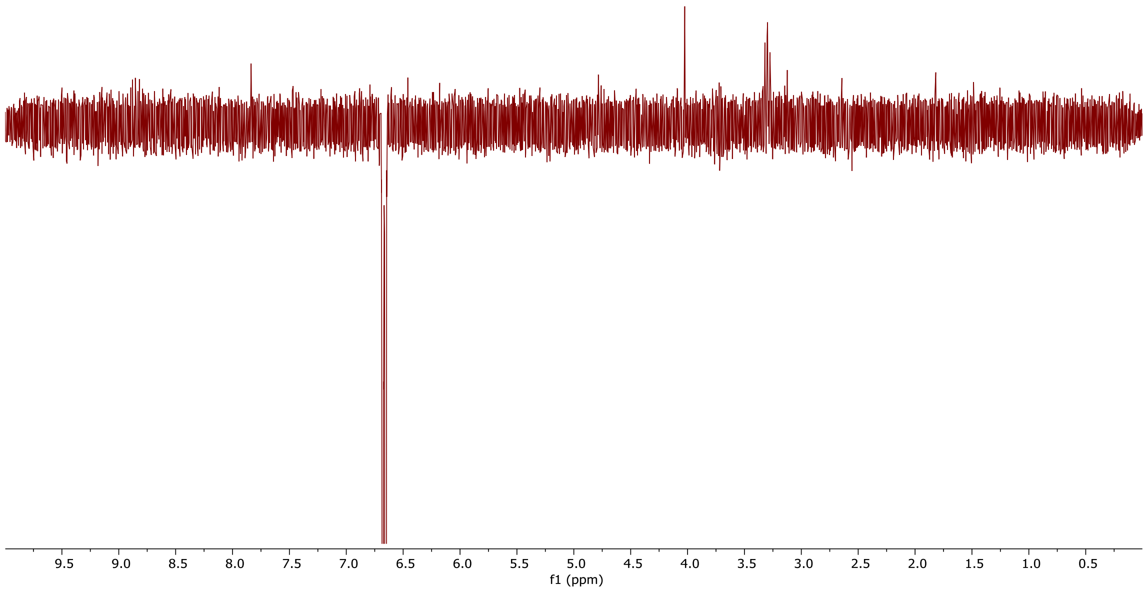

Figure S78. ^{1}H selective 1D-NOESY ($CDCl_3$) of **3b**

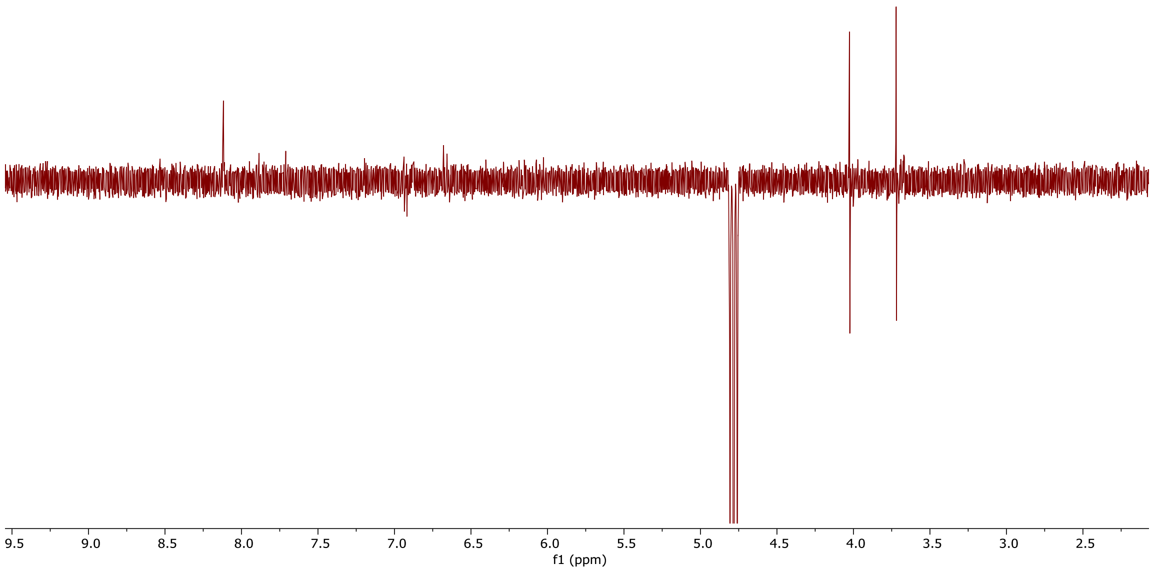

Figure S79. ^{1}H selective 1D-NOESY ($CDCl_3$) of **3b**

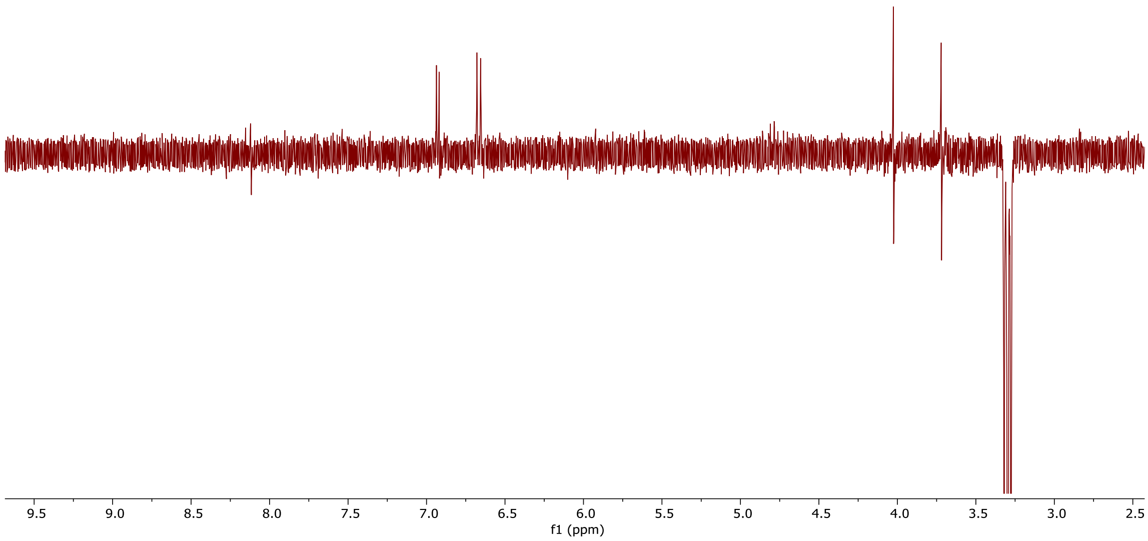

Figure S80. ^{1}H selective 1D-NOESY ($CDCl_3$) of **3b**

2.- Absorption spectra of oxazolones 1a, 1b, 1c and **1f**

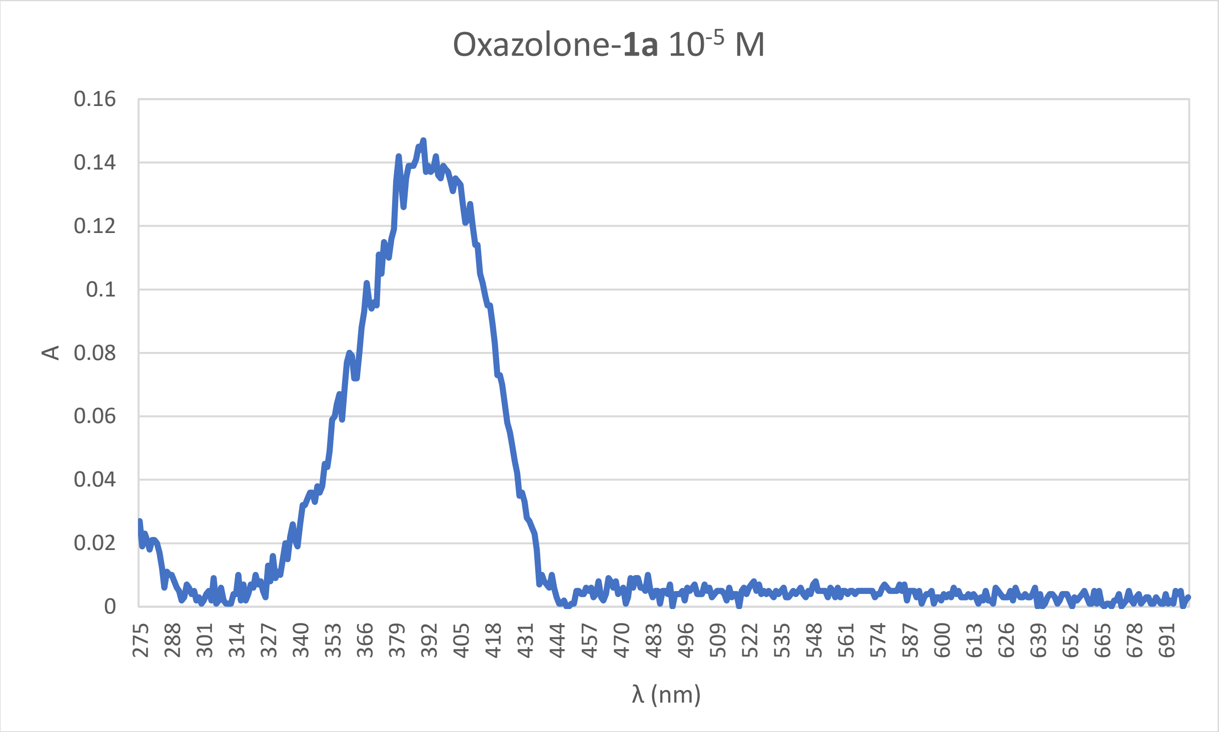

Figure S81. Absorption spectrum of oxazolone **1a**

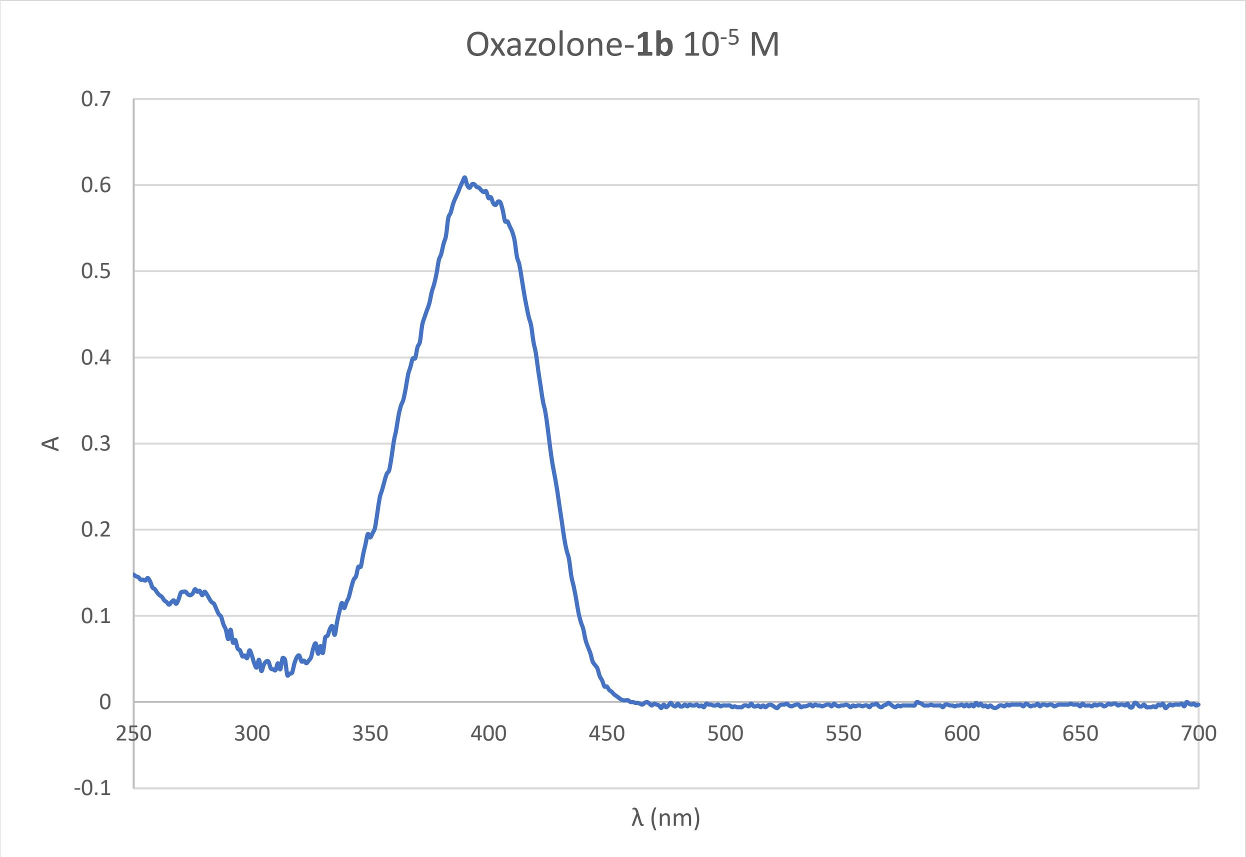

Figure S82. Absorption spectrum of oxazolone **1b**

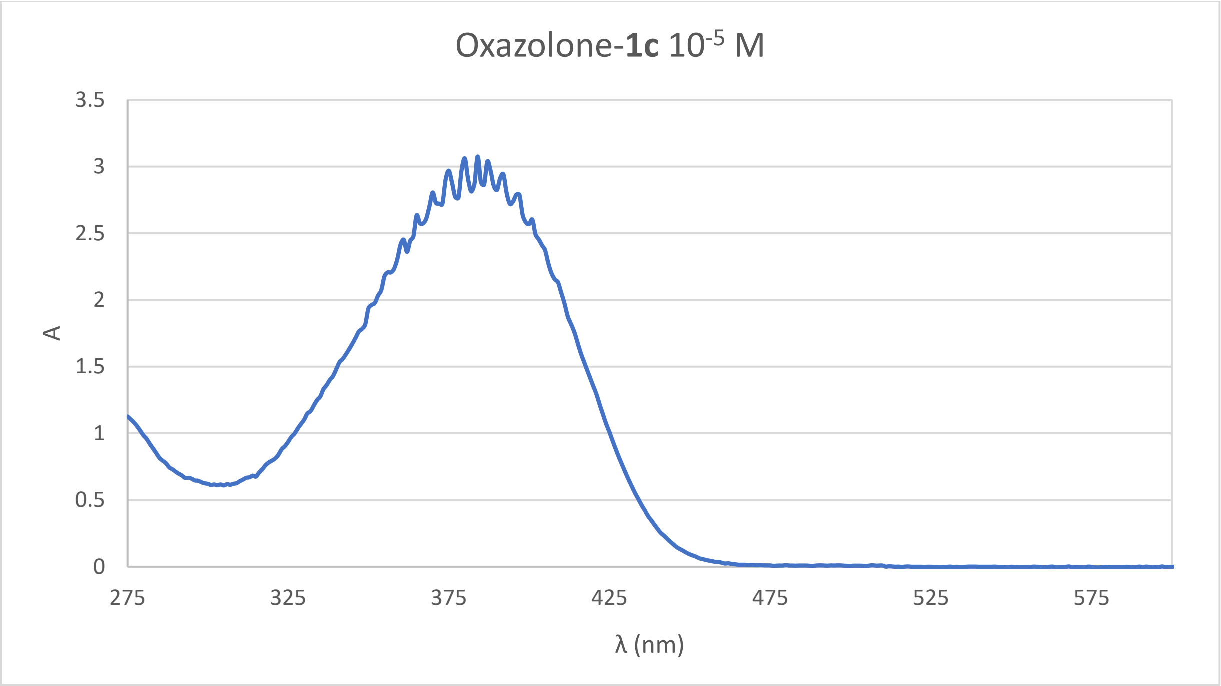

Figure S83. Absorption spectrum of oxazolone **1c**

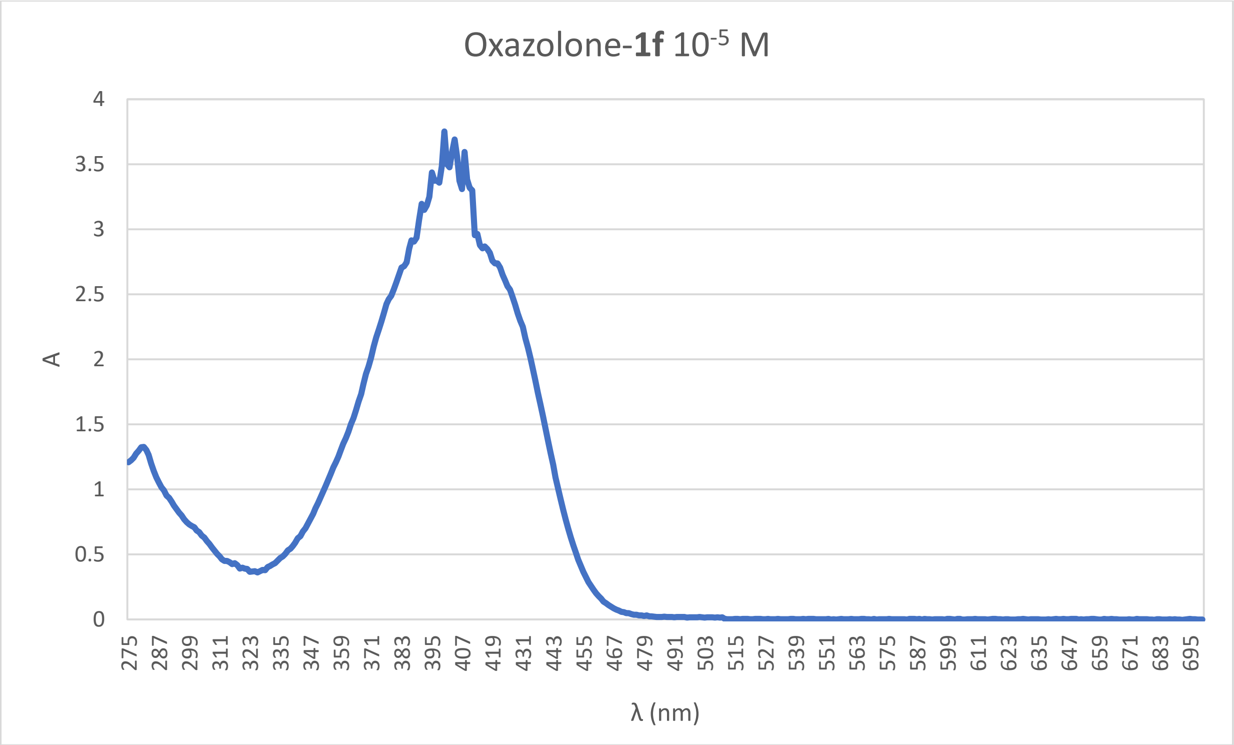

Figure S84. Absorption spectrum of oxazolone **1f**

3.- Transient absorption spectra

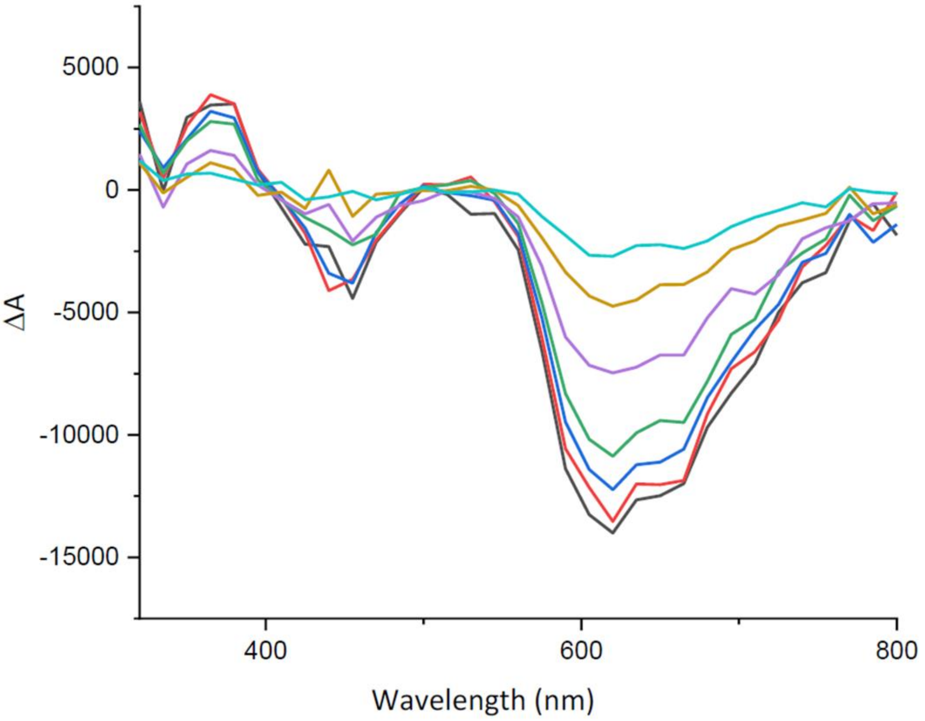

Figure S85. Transient absorption spectra of a deoxygenated CH_2Cl_2 solution of $Ru(bpy)_3^{2+}$ recorded at different times after the laser pulse: 8 ns (black); 44 ns (red); 124 ns (blue); 224 ns (green); 552 ns (purple); 924 ns (ochre) and 1376 ns (light blue) (λexc = 532 nm).

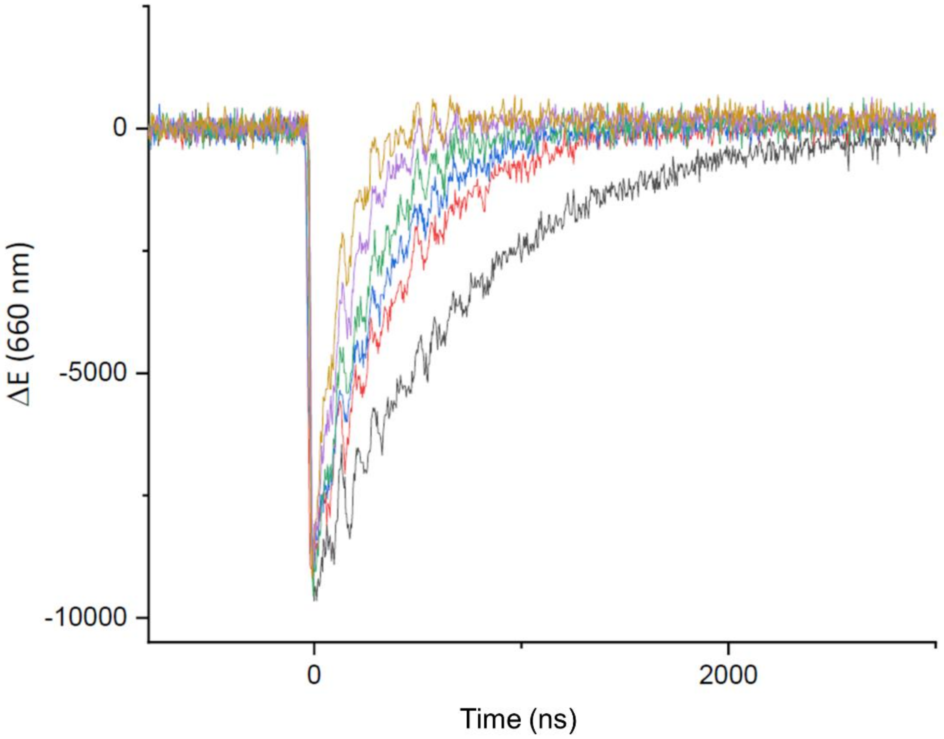

Figure S86. Decay traces recorded at 660 nm for $Ru(bpy)_3^{2+}$ (in deoxygenated CH_2Cl_2) upon addition of different amounts of **1b**: 0 (black); $3.3 \cdot 10^{-4}$ M (red); $6.6 \cdot 10^{-4}$ M (blue); $9.9 \cdot 10^{-4}$ M (green), $1.6 \cdot 10^{-3}$ M (purple) y $2.4 \cdot 10^{-3}$ M (ochre), obtained after LFP excitation (532 nm).

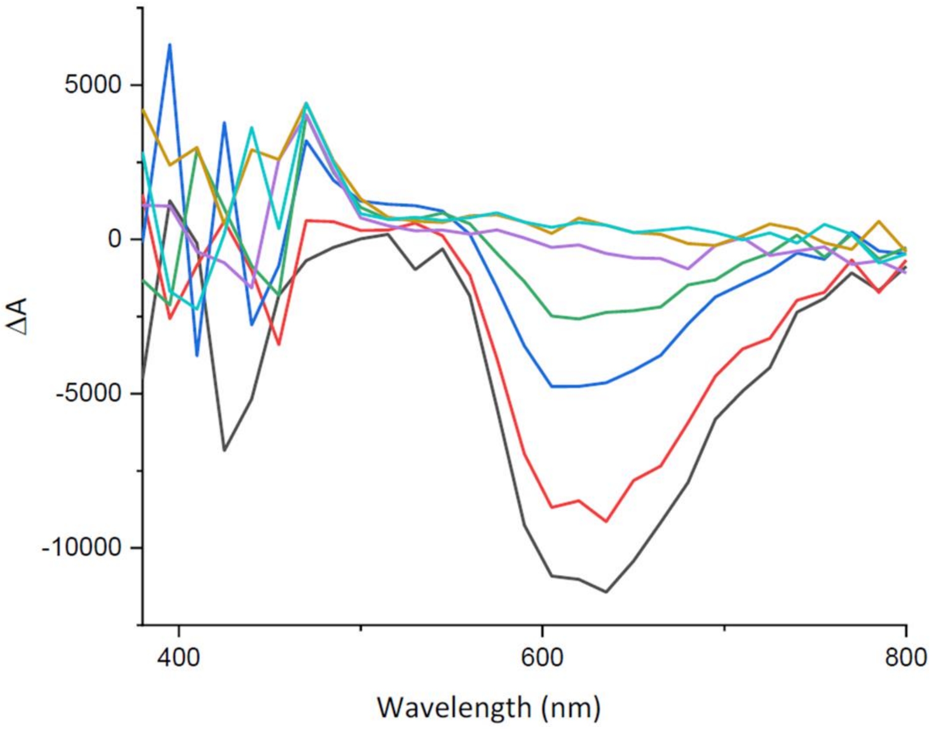

Figure S87. Transient absorption spectra for deoxygenated CH_2Cl_2 solutions of $Ru(bpy)_3^{2+}$ in presence of **1b** ($2.4{\cdot}10^{-3}$M) recorded at different times after the laser pulse (λ_{exc} = 532 nm): 12 ns (black); 44 ns (red); 124 ns (dark blue); 224 ns (green); 552 ns (purple); 924 ns (ochre) and 1376 ns (light blue).

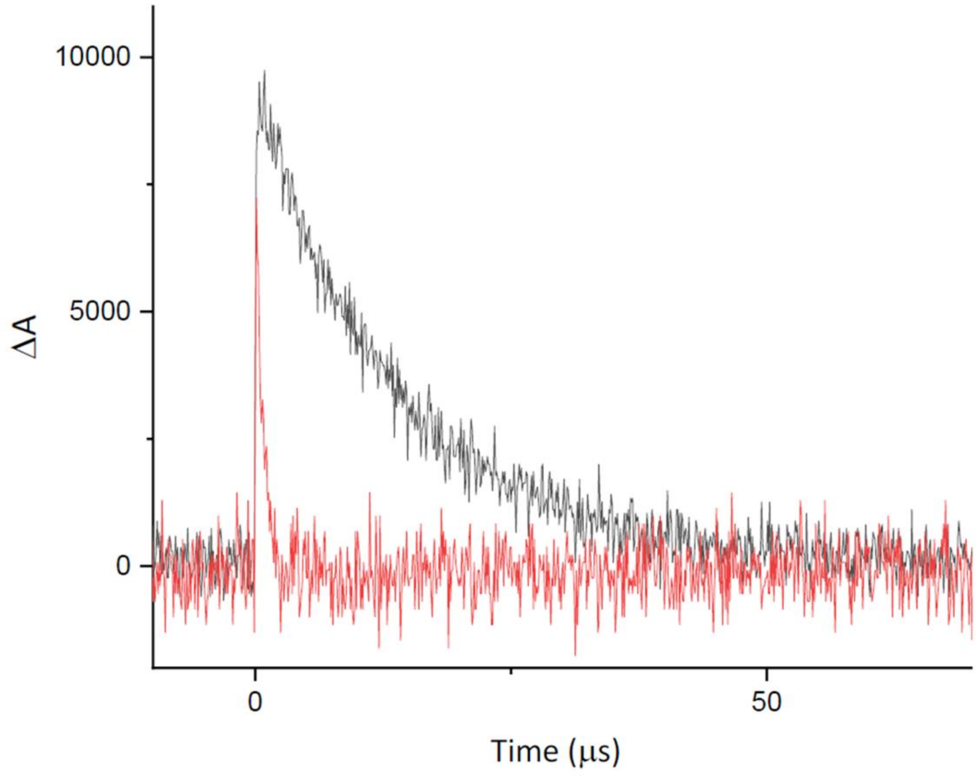

Figure S88. Transient absorption traces recorded at 470 nm upon LFP excitation (532 nm) of $Ru(bpy)_3^{2+}$ in the presence of **1b** ($4.7{\cdot}10^{-3}$ M) in deoxygenated (black) and oxygenated CH_2Cl_2 (red).

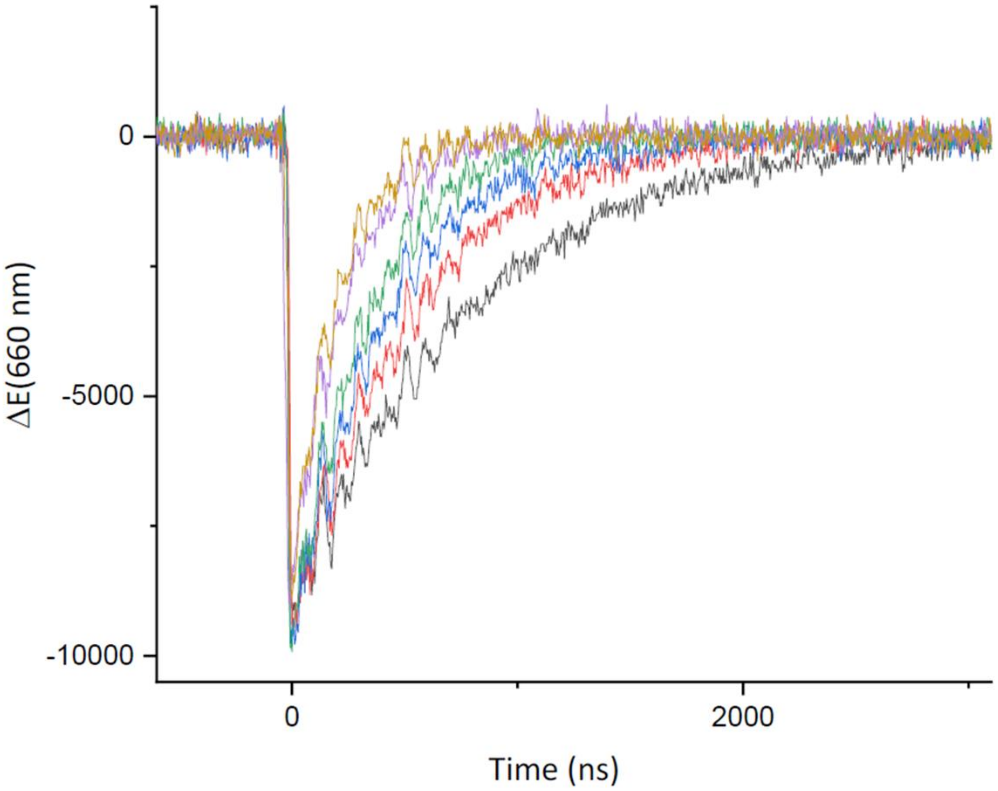

Figure S89. Decay traces recorded at 660 nm for $Ru(bpy)_3^{2+}$ (in deoxygenated CH_2Cl_2) upon addition of different amounts of **1d**: 0 (black); $3.3 \cdot 10^{-4}$ M (red); $6.6 \cdot 10^{-4}$ M (blue); $9.9 \cdot 10^{-4}$ M (green), $1.6 \cdot 10^{-3}$ M (purple) y $2.4 \cdot 10^{-3}$ M (ochre), obtained after LFP excitation (532 nm).

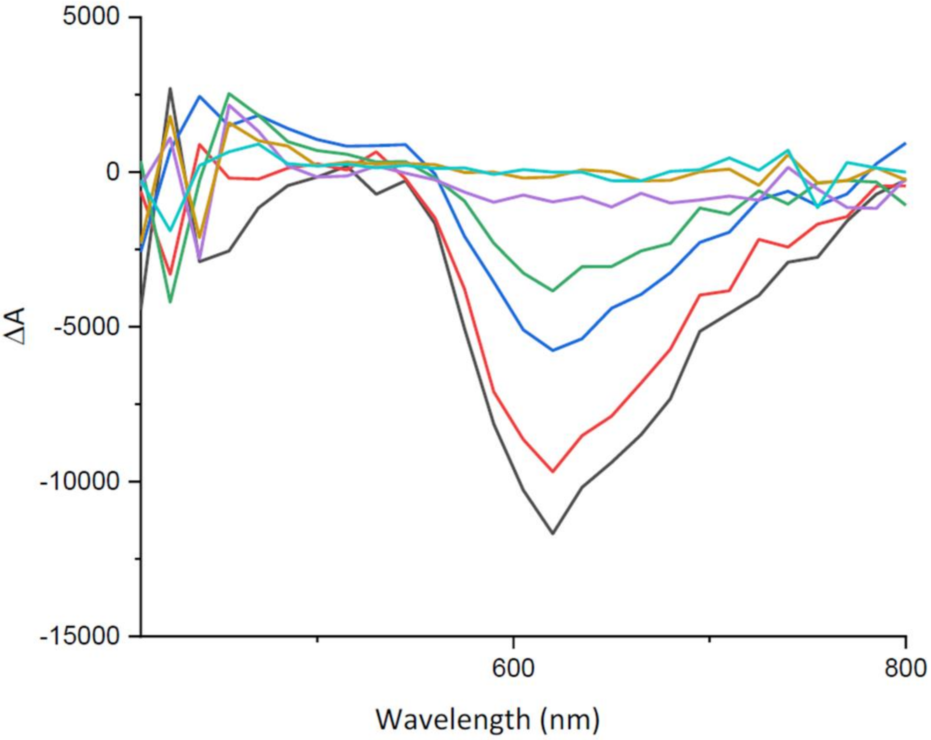

Figure S90. Transient absorption spectra for deoxygenated CH_2Cl_2 solutions of $Ru(bpy)_3^{2+}$ in presence of **1d** ($2.4 \cdot 10^{-3}$M) recorded at different times after the laser pulse (λ_{exc} = 532 nm): 12 ns (black); 44 ns (red); 124 ns (dark blue); 224 ns (green); 552 ns (purple); 924 ns (ochre) y 1376 ns (blue).

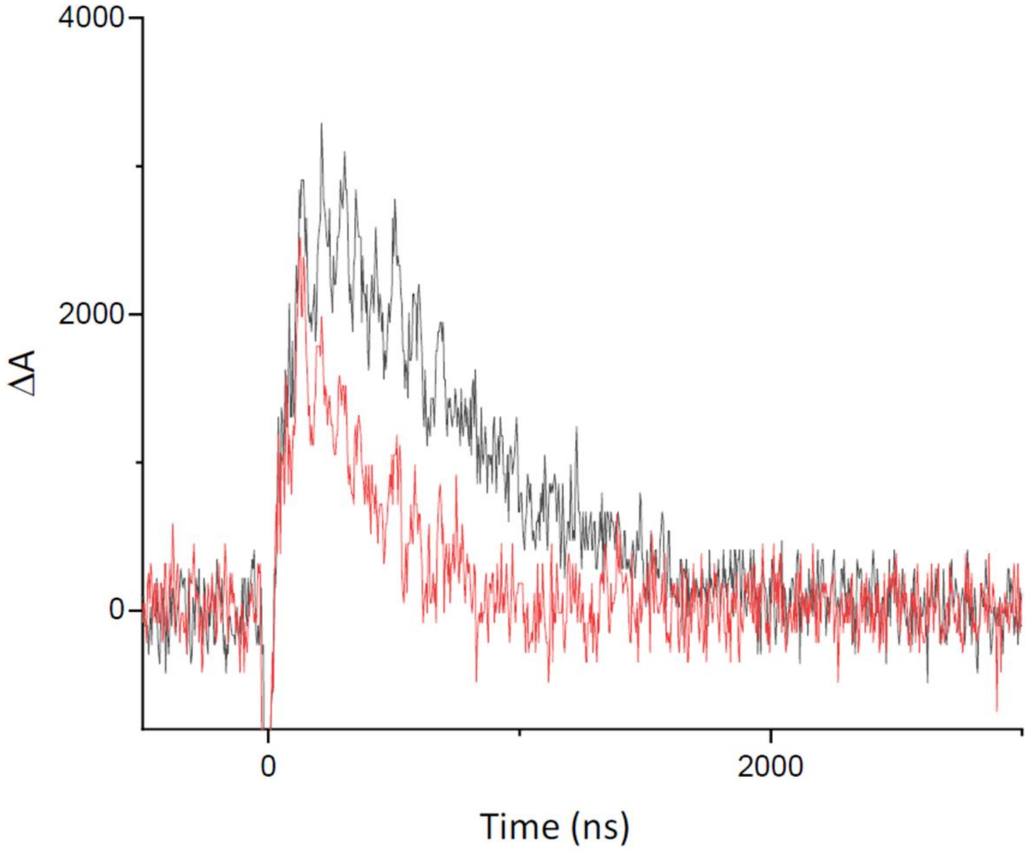

Figure S91. Transient absorption traces recorded at 470 nm upon LFP excitation (532 nm) of $Ru(bpy)_3^{2+}$ in the presence of **1d** ($4.7 \cdot 10^{-3}$ M) in deoxygenated (black) and oxygenated CH_2Cl_2 (red).

Table S1. Half-life values at 470 nm and ruthenium deactivation rate constant at 660 nm.

	**1a**	**1b**	**1d**
**Half-life time (T1)**	12.15 μs	10.72 μs	726.1 ns
**Deactivation rate (k_q)**	$4.25 \cdot 10^{10}$ $M^{-1}s^{-1}$	$3.94 \cdot 10^{10}$ $M^{-1}s^{-1}$	$5.61 \cdot 10^{10}$ $M^{-1}s^{-1}$

4.- Cyclic Voltammetry of oxazolone 1a

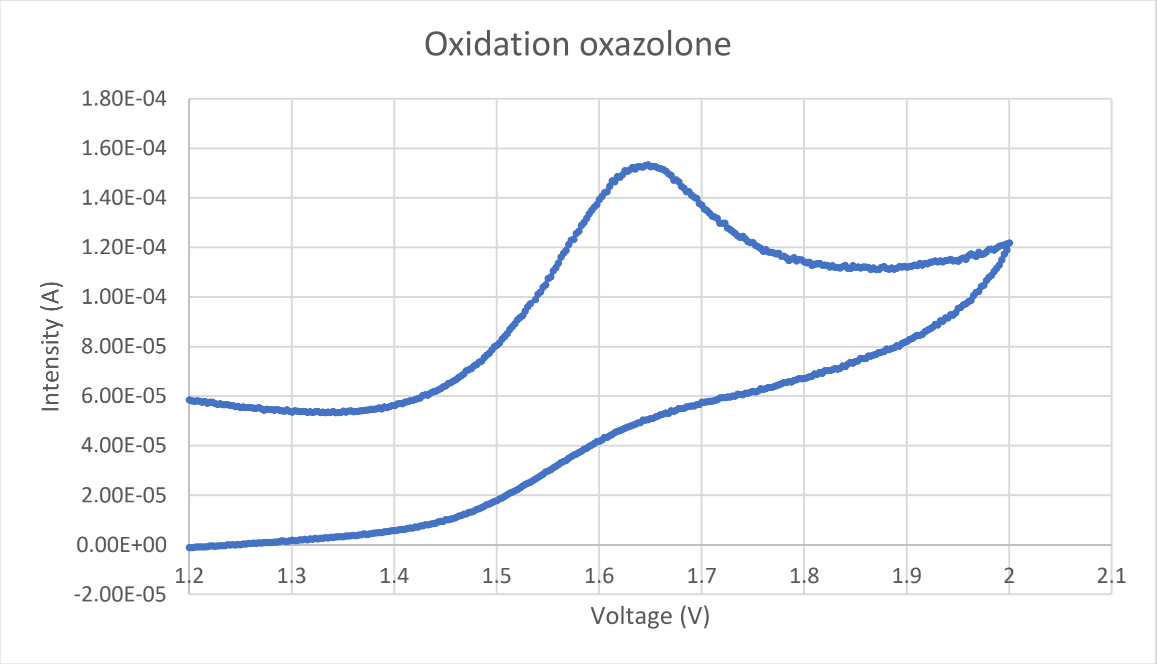

Figure S92. Oxidation wave of the oxazolone **1a**

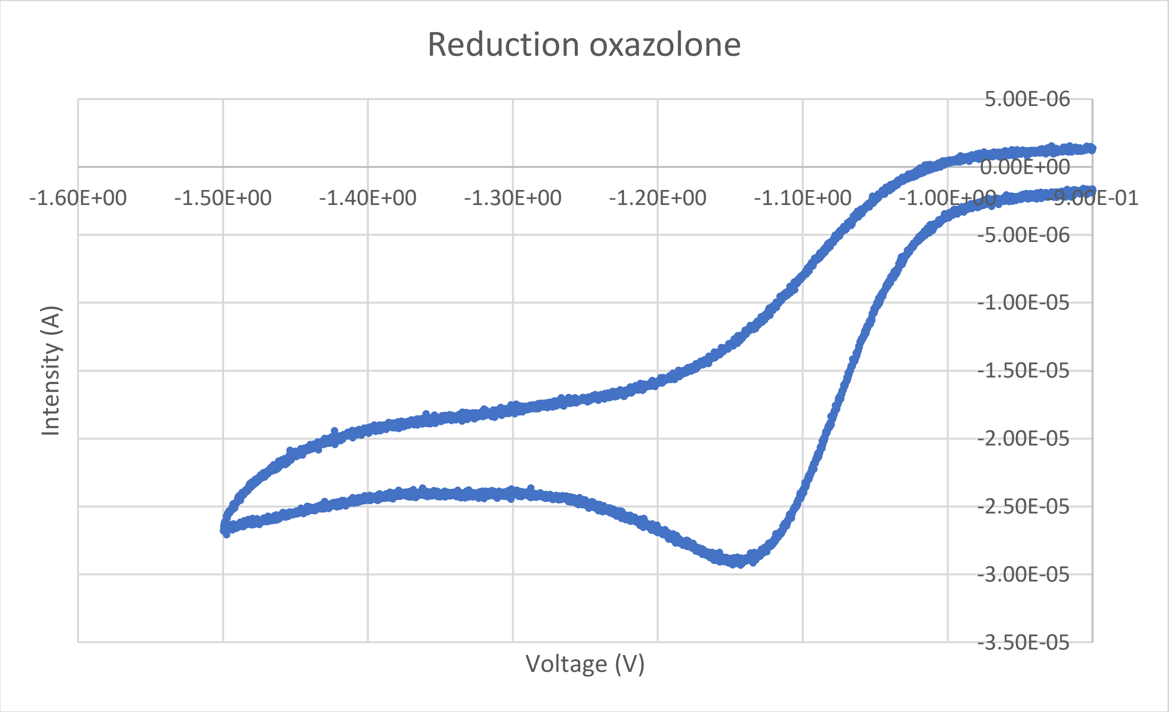

Figure S93. Reduction wave of the oxazolone **1a**

5. X-ray crystallographic data

Table S2. Crystal data and structure refinement for 1a.

Empirical formula	$C_{18}H_{13}NO_2$	
Formula weight	275.29	
Temperature	100(2) K	
Wavelength	0.71073 Å	
Crystal system	Monoclinic	
Space group	P 21/c	
Unit cell dimensions	a = 11.8733(8) Å	α= 90°.
	b = 3.8210(3) Å	β= 100.315(2)°.
	c = 29.729(2) Å	γ = 90°.
Volume	1326.94(16) $Å^3$	
Z	4	
Density (calculated)	1.378 Mg/m^3	
Absorption coefficient	0.090 mm^{-1}	
F(000)	576	
Crystal size	0.154 x 0.142 x 0.055 mm^3	
Theta range for data collection	2.42 to 28.32°.	
Index ranges	-15<=h<=15, -5<=k<=5, -39<=l<=39	
Reflections collected	28142	
Independent reflections	3284 [R(int) = 0.0497]	
Completeness to theta = 28.32°	99.8 %	
Absorption correction	Semi-empirical from equivalents	
Max. and min. transmission	0.99 and 0.91	
Refinement method	Full-matrix least-squares on F^2	
Data / restraints / parameters	3284 / 0 / 193	
Goodness-of-fit on F^2	1.179	
Final R indices [I>2sigma(I)]	R1 = 0.0664, wR2 = 0.1823	
R indices (all data)	R1 = 0.0803, wR2 = 0.1895	
Largest diff. peak and hole	0.375 and -0.324 e.$Å^{-3}$	

Table S3. Atomic coordinates (x 10^4) and equivalent isotropic displacement parameters (Å^{2}x 10^3) for 1a. U(eq) is defined as one third of the trace of the orthogonalized U^{ij} tensor.

	x	y	z	U(eq)
O(1)	5297(2)	2098(5)	5830(1)	19(1)
N(1)	4310(2)	4972(6)	6303(1)	18(1)
C(1)	4320(2)	3893(7)	5893(1)	17(1)
O(2)	6915(2)	556(6)	6316(1)	25(1)
C(2)	5361(2)	3916(7)	6561(1)	17(1)
C(3)	5998(2)	2007(7)	6258(1)	18(1)
C(4)	3429(2)	4354(7)	5494(1)	16(1)
C(5)	3583(2)	3244(8)	5060(1)	19(1)
C(8)	1524(2)	6358(8)	5174(1)	21(1)
C(7)	1679(2)	5225(8)	4740(1)	22(1)
C(6)	2710(2)	3694(8)	4684(1)	20(1)
C(9)	2398(2)	5924(7)	5549(1)	18(1)
C(10)	5720(2)	4709(8)	7010(1)	19(1)
C(11)	6818(2)	3923(7)	7273(1)	19(1)
C(12)	7107(2)	5019(7)	7713(1)	18(1)
C(13)	8210(2)	4655(7)	8015(1)	16(1)
C(14)	9185(2)	3223(7)	7875(1)	20(1)
C(15)	10221(2)	3123(8)	8176(1)	21(1)
C(16)	10314(2)	4414(8)	8619(1)	21(1)
C(17)	9353(2)	5776(8)	8764(1)	21(1)
C(18)	8313(2)	5926(7)	8462(1)	18(1)

Table S4. Bond lengths [Å] and angles [°] for 1a.

O(1)-C(1)	1.389(3)
O(1)-C(3)	1.393(3)
N(1)-C(1)	1.289(3)
N(1)-C(2)	1.402(3)
C(1)-C(4)	1.452(4)
O(2)-C(3)	1.207(3)
C(2)-C(10)	1.361(4)
C(2)-C(3)	1.469(4)
C(4)-C(9)	1.398(4)
C(4)-C(5)	1.400(4)
C(5)-C(6)	1.393(4)
C(5)-H(5)	0.95
C(8)-C(9)	1.391(4)
C(8)-C(7)	1.404(4)
C(8)-H(8)	0.95
C(7)-C(6)	1.392(4)
C(7)-H(7)	0.95
C(6)-H(6)	0.95
C(9)-H(9)	0.95
C(10)-C(11)	1.427(4)
C(10)-H(10)	0.95
C(11)-C(12)	1.358(4)
C(11)-H(11)	0.95
C(12)-C(13)	1.455(4)
C(12)-H(12)	0.95
C(13)-C(18)	1.400(4)
C(13)-C(14)	1.408(4)
C(14)-C(15)	1.386(4)
C(14)-H(14)	0.95
C(15)-C(16)	1.392(4)
C(15)-H(15)	0.95
C(16)-C(17)	1.390(4)
C(16)-H(16)	0.95
C(17)-C(18)	1.392(4)
C(17)-H(17)	0.95
C(18)-H(18)	0.95

C(1)-O(1)-C(3)	105.39(19)
C(1)-N(1)-C(2)	105.6(2)
N(1)-C(1)-O(1)	115.8(2)
N(1)-C(1)-C(4)	127.3(2)
O(1)-C(1)-C(4)	116.9(2)
C(10)-C(2)-N(1)	124.4(2)
C(10)-C(2)-C(3)	127.3(2)
N(1)-C(2)-C(3)	108.2(2)
O(2)-C(3)-O(1)	121.2(2)
O(2)-C(3)-C(2)	133.8(2)
O(1)-C(3)-C(2)	105.0(2)
C(9)-C(4)-C(5)	120.0(2)
C(9)-C(4)-C(1)	118.7(2)
C(5)-C(4)-C(1)	121.3(2)
C(6)-C(5)-C(4)	120.0(2)
C(6)-C(5)-H(5)	120.0
C(4)-C(5)-H(5)	120.0
C(9)-C(8)-C(7)	119.9(2)
C(9)-C(8)-H(8)	120.0
C(7)-C(8)-H(8)	120.0
C(6)-C(7)-C(8)	120.1(3)
C(6)-C(7)-H(7)	120.0
C(8)-C(7)-H(7)	120.0
C(7)-C(6)-C(5)	120.0(3)
C(7)-C(6)-H(6)	120.0
C(5)-C(6)-H(6)	120.0
C(8)-C(9)-C(4)	119.9(2)
C(8)-C(9)-H(9)	120.0
C(4)-C(9)-H(9)	120.0
C(2)-C(10)-C(11)	125.8(3)
C(2)-C(10)-H(10)	117.1
C(11)-C(10)-H(10)	117.1
C(12)-C(11)-C(10)	120.8(3)
C(12)-C(11)-H(11)	119.6
C(10)-C(11)-H(11)	119.6
C(11)-C(12)-C(13)	127.5(2)
C(11)-C(12)-H(12)	116.2
C(13)-C(12)-H(12)	116.2
C(18)-C(13)-C(14)	118.6(2)
C(18)-C(13)-C(12)	117.9(2)

C(14)-C(13)-C(12)	123.5(2)
C(15)-C(14)-C(13)	120.2(2)
C(15)-C(14)-H(14)	119.9
C(13)-C(14)-H(14)	119.9
C(14)-C(15)-C(16)	120.7(3)
C(14)-C(15)-H(15)	119.6
C(16)-C(15)-H(15)	119.6
C(17)-C(16)-C(15)	119.7(2)
C(17)-C(16)-H(16)	120.2
C(15)-C(16)-H(16)	120.2
C(16)-C(17)-C(18)	119.9(3)
C(16)-C(17)-H(17)	120.0
C(18)-C(17)-H(17)	120.0
C(17)-C(18)-C(13)	120.9(2)
C(17)-C(18)-H(18)	119.5
C(13)-C(18)-H(18)	119.5

Table S5. Anisotropic displacement parameters ($Å^2$x 10^3) for 1a. The anisotropic displacement factor exponent takes the form: $-2\pi^2[h^2a^{*2}U^{11} + ... + 2 h k a^* b^* U^{12}]$

	U^{11}	U^{22}	U^{33}	U^{23}	U^{13}	U^{12}
O(1)	16(1)	24(1)	18(1)	0(1)	6(1)	4(1)
N(1)	15(1)	18(1)	19(1)	0(1)	3(1)	3(1)
C(1)	16(1)	16(1)	21(1)	2(1)	6(1)	0(1)
O(2)	18(1)	33(1)	23(1)	-1(1)	4(1)	7(1)
C(2)	16(1)	18(1)	18(1)	0(1)	5(1)	0(1)
C(3)	16(1)	21(1)	17(1)	0(1)	5(1)	1(1)
C(4)	17(1)	14(1)	19(1)	1(1)	6(1)	-1(1)
C(5)	18(1)	19(1)	21(1)	0(1)	6(1)	1(1)
C(8)	20(1)	20(1)	23(1)	0(1)	6(1)	2(1)
C(7)	21(1)	24(1)	21(1)	2(1)	3(1)	2(1)
C(6)	20(1)	20(1)	19(1)	-1(1)	3(1)	0(1)
C(9)	17(1)	19(1)	19(1)	0(1)	4(1)	1(1)
C(10)	20(1)	20(1)	18(1)	-1(1)	5(1)	-1(1)
C(11)	19(1)	17(1)	20(1)	0(1)	4(1)	1(1)
C(12)	18(1)	18(1)	20(1)	1(1)	4(1)	-1(1)
C(13)	16(1)	16(1)	16(1)	0(1)	4(1)	-1(1)
C(14)	22(1)	18(1)	19(1)	0(1)	6(1)	2(1)
C(15)	19(1)	22(1)	22(1)	3(1)	6(1)	3(1)
C(16)	17(1)	25(1)	21(1)	2(1)	4(1)	-2(1)
C(17)	19(1)	24(1)	19(1)	-2(1)	4(1)	-4(1)
C(18)	17(1)	20(1)	19(1)	-2(1)	6(1)	-2(1)

Table S6. Torsion angles [°] for 1a.

C(2)-N(1)-C(1)-O(1)	0.4(3)
C(2)-N(1)-C(1)-C(4)	-179.5(3)
C(3)-O(1)-C(1)-N(1)	0.5(3)
C(3)-O(1)-C(1)-C(4)	-179.7(2)
C(1)-N(1)-C(2)-C(10)	176.3(3)
C(1)-N(1)-C(2)-C(3)	-1.0(3)
C(1)-O(1)-C(3)-O(2)	178.9(3)
C(1)-O(1)-C(3)-C(2)	-1.0(3)
C(10)-C(2)-C(3)-O(2)	4.1(5)
N(1)-C(2)-C(3)-O(2)	-178.6(3)
C(10)-C(2)-C(3)-O(1)	-176.0(3)
N(1)-C(2)-C(3)-O(1)	1.3(3)
N(1)-C(1)-C(4)-C(9)	-4.7(4)
O(1)-C(1)-C(4)-C(9)	175.5(2)
N(1)-C(1)-C(4)-C(5)	175.7(3)
O(1)-C(1)-C(4)-C(5)	-4.2(4)
C(9)-C(4)-C(5)-C(6)	-0.2(4)
C(1)-C(4)-C(5)-C(6)	179.5(3)
C(9)-C(8)-C(7)-C(6)	-0.6(4)
C(8)-C(7)-C(6)-C(5)	0.8(4)
C(4)-C(5)-C(6)-C(7)	-0.5(4)
C(7)-C(8)-C(9)-C(4)	-0.1(4)
C(5)-C(4)-C(9)-C(8)	0.5(4)
C(1)-C(4)-C(9)-C(8)	-179.2(3)
N(1)-C(2)-C(10)-C(11)	-174.6(3)
C(3)-C(2)-C(10)-C(11)	2.2(5)
C(2)-C(10)-C(11)-C(12)	176.2(3)
C(10)-C(11)-C(12)-C(13)	-175.3(3)
C(11)-C(12)-C(13)-C(18)	-178.8(3)
C(11)-C(12)-C(13)-C(14)	3.8(5)
C(18)-C(13)-C(14)-C(15)	-0.7(4)
C(12)-C(13)-C(14)-C(15)	176.8(3)
C(13)-C(14)-C(15)-C(16)	0.3(4)
C(14)-C(15)-C(16)-C(17)	0.9(4)

C(15)-C(16)-C(17)-C(18)	-1.7(4)
C(16)-C(17)-C(18)-C(13)	1.3(4)
C(14)-C(13)-C(18)-C(17)	-0.2(4)
C(12)-C(13)-C(18)-C(17)	-177.7(3)

6.- Computational Details

6.1.- Computational methods

The range separated dispersion-corrected ωB97X-D[1,2] functional and the 6-31+G(d)[3,4,5,6,7] basis set were used to optimize the geometries of all stationary points. Vibrational frequency calculations were used to confirm that stationary points were either minima or first-order saddle points on the potential energy surface and to obtain frequencies used to calculate thermochemistry values with the *GoodVibes*[8] program. For more information about calculating thermodata, see the *Thermochemical Data Calculation with GoodVibes* section. Energies were refined with single-point energy calculations at the ωB97X-D/def2-QZVPP level using the optimized structures. In all cases, the calculations included the integral equation formalism variant of the polarizable continuum model (IEF-PCM)[9,10,11,12,13] with the SMD[14] solvation model (solvent=dichloromethane) to account for solvent effects.

Gaussian 16[15] was employed for all density functional theory (DFT) calculations, using an "ultrafine" pruned (99,590) grid for numerical integration of the exchange-correlation functional and its derivatives (default in version 16). The display settings of *Pymol* created by Prof. Robert Paton were used.[16] Natural spin populations were computed using natural population analysis (NPA) with *NBO 7.0*,[17] interfaced to *Gaussian 16*. The *Jprogdyn*[18] program was used to perform molecular dynamics trajectories, interfaced to *Gaussian 16*.

AQME[19] was used to automate all the tasks related to generation, calculations and analysis of static DFT calculations, including 1) generation of molecular coordinates with the *CREST* program,[20] 2) creation of *Gaussian 16* inputs, and 3) analysis of optimization and frequency jobs with cclib[21] (see the *Automation with AQME* section).

6.2.- Thermochemical data and molecular coordinates obtained with *GoodVibes*

The final step of the automated workflow uses the *GoodVibes* program to introduce quasi-harmonic (QHA) corrections to the computed vibrational entropies using a frequency cut-off value of 100.0 cm^{-1}, following the model proposed by Grimme[22] at 298.15 K. Also, a correction for the change in standard state from gas phase at 1 atm to a 1 M solution was introduced (option "-c 1" in *GoodVibes*).[23]

All the thermochemical data including absolute energies, zero-point energies (ZPE) and T·S, among other parameters, at the ωB97X-D/6-31+G(d) level, as well as the absolute energies, corrected final G and relative G obtained with ωB97X-D/def2-QZVPP, were generated in an automated way using *GoodVibes* and tabulated in a separate file of the ESI (*Thermochemistry.dat* in the *Extra_ESI.zip* file). Molecular coordinates were generated similarly using the "--xyz" option (*Molecular_coordinates.dat* in the *Extra_ESI.zip* file).

6.3.- Automation with *AQME*

AQME version used: 1.4.8.

Reproduce the workflow: in a SLURM-based high-performance computing (HPC) cluster, run "sbatch AQME_script.sh". The *AQME_script.sh*, *CSEARCH_SMILES.csv* and *Grel.yaml* files are required. In the Centro de Supercomputación de Galicia (CESGA)'s Finis Terrae III cluster, the whole workflow ended in approximately eight hours (elapsed time) when using 48 processors.

Data availability: the raw outputs of this workflow obtained from running *AQME_script.sh*, the input *AQME_script.sh*, *CSEARCH_SMILES.csv* and *Grel.yaml* files, and the *Mol_visualizer.ipynb* notebook used to set constraints can be found in the *ioChem-DB* database[24] (https://iochem-

bd.bsc.es/browse/handle/100/281001, file name: *AQME_workflow.zip*). Separately, the OPT+FREQ calculations were also uploaded to *ioChem-DB* to allow for the 3D visualization of the structures and their vibrational modes (DOI: 10.19061/iochem-bd-6-227).

This automated workflow generated the following files from *AQME*, *cclib* and *GoodVibes*:

- Processed LOG output files from OPT+FREQ calculations and single-point energy corrections from *Gaussian 16* (folder: /QCALC/success)
- JSON files with *cclib* properties and calculation information of the output files (folder: /QCALC/success/json_files)
- Raw thermochemical data from *GoodVibes* (*Goodvibes_output.dat* file, in the parent directory)
- XYZ files generated by *GoodVibes* (*Goodvibes_output.xyz* file, in the parent directory)

<u>Workflow steps:</u>

Step	AQME module	Task	External program used
1	None	Draw structures in ChemDraw and copy SMILES in the *CSEARCH_SMILES.csv* file.	*ChemDraw* and *Microsoft Excel*
2	None	Determining atom numbering to apply constraints in dihedral angles using the *Mol_visualizer.ipynb* notebook. The constraints are needed to model the major isomer (*E,Z*) selectively and to set the dihedral angles for rotation transition states.	*Visual Studio Code*
3	CSEARCH	Conformer sampling.	*CREST*
Command line in the *AQME_script.sh* file:<br>python -m aqme --csearch --program "crest" --input "CSEARCH_SMILES.csv" --nprocs "$totalcpu"			
4	QPREP	Generation of *Gaussian* input files for OPT+FREQ calculations.	None
Command lines in the *AQME_script.sh* file (different for ground and transition states):<br>python -m aqme --qprep --program "gaussian" --files "CSEARCH/S0*.sdf" --qm_input "opt=(calcfc) freq=noraman wb97xd/6-31+g(d) scrf=(smd,solvent=dichloromethane)" --mem "$childmem" --nprocs "$childcpu"<br>python -m aqme --qprep --program "gaussian" --files "CSEARCH/T1*.sdf" --qm_input "opt=(calcfc) freq=noraman wb97xd/6-31+g(d) scrf=(smd,solvent=dichloromethane)" --mem "$childmem" --nprocs "$childcpu"<br>python -m aqme --qprep --program "gaussian" --files "CSEARCH/TS*.sdf" --qm_input "opt=(calcfc,ts,noeigen,maxstep=5) freq=noraman wb97xd/6-31+g(d) scrf=(smd,solvent=dichloromethane)" --mem "$childmem" --nprocs "$childcpu"			
5	None	Run *Gaussian* OPT+FREQ jobs.	*Gaussian 16*
Command lines in the *AQME_script.sh* file:			

<table>
<tr><td colspan="4">gexc "$excdir/QCALC" $jbatch</td></tr>
<tr><td>6</td><td>QCORR</td><td>Processing of Gaussian output files and automatic creation of new input files to fix errors and imaginary frequencies.</td><td>cclib</td></tr>
<tr><td colspan="4">Command line in the AQME_script.sh file:
python -m aqme --qcorr --files "$excdir/QCALC/*log" --mem "$childmem" --nprocs "$childcpu" --freq_conv 'opt=(calcfc,maxstep=5)' --isom_type 'com' --isom_inputs "$excdir/QCALC" --amplitude_ifreq 0.3</td></tr>
<tr><td>7</td><td>None</td><td>Run corrected inputs with Gaussian (not necessary in the example provided, as all the initial Gaussian jobs ended successfully).</td><td>Gaussian 16</td></tr>
<tr><td colspan="4">Command line in the AQME_script.sh file:
gexc "$excdir/QCALC/failed/run_1/fixed_QM_inputs" $jbatch</td></tr>
<tr><td>8</td><td>QCORR</td><td>Processing of the second round of Gaussian output files (not necessary in the example provided, as all the initial Gaussian jobs ended successfully).</td><td>cclib</td></tr>
<tr><td colspan="4">Command line in the AQME_script.sh file:
python -m aqme --qcorr --files "$excdir/QCALC/failed/run_1/fixed_QM_inputs/*.log" --mem "$childmem" --nprocs "$childcpu" --isom_type 'com' --isom_inputs "$excdir/QCALC/failed/run_1/fixed_QM_inputs"</td></tr>
<tr><td>9</td><td>QPREP</td><td>Generation of Gaussian input files for single-point energy corrections.</td><td>None</td></tr>
<tr><td colspan="4">Command line in the AQME_script.sh file:
python -m aqme --qprep --program gaussian --files "$excdir/QCALC/success/*.log" --destination "$excdir/QCALC/success" --qm_input 'wb97xd/def2qzvpp scrf=(smd,solvent=dichloromethane)' --mem "$childmem" --nprocs "$childcpu" --suffix "SPC"</td></tr>
<tr><td>10</td><td>None</td><td>Run Gaussian single-point energy jobs.</td><td>Gaussian 16</td></tr>
<tr><td colspan="4">Command line in the AQME_script.sh file:
gexc "$excdir/QCALC/success" $jbatch</td></tr>
<tr><td>11</td><td>None</td><td>Thermochemistry calculation, generation of relative G values, analysis of imaginary frequencies and creation of an XYZ file containing all the molecular coordinates.</td><td>GoodVibes</td></tr>
<tr><td colspan="4">Command line in the AQME_script.sh file:</td></tr>
</table>

```
python -m goodvibes --xyz -c 1 "$excdir/QCALC/success/*.log" --spc SPC --imag --pes Grel.yaml
```

6.4.- MD simulations

MD trajectories were run starting from **T1-1a-EZ_rdkit_conf_1** and **T1-4_rdkit_conf_1**, in which the θ dihedral angle was tracked (Figure 9B). Negative θ values were made positive in the analysis to allow for a better comparison of the θ variations between **1a** and **4**. In total, 12 trajectories of 1000 fs were considered for each species. The SMD implicit solvation model was employed in the simulations instead of explicit solvent molecules. This approach assumes that the energy exchange between solute and dichloromethane is negligible since the time scale of the simulations is considerably inferior to previously measured solute-solvent energy exchanges (on the order of 10 to a few hundred picoseconds).[25] All the xyz coordinates and θ values are included in the *Extra_ESI.zip* file uploaded with the ESI. Additionally, a Jupyter Notebook with the analysis workflow is provided to increase the transparency and reproducibility of the MD protocol.

The input used in Jprogdyn is described below (only the forward direction was considered).

```
### Jprogdyn Configuration File ###

# File/Directory Locations
working_directory              : use_current
frequency_directory            : STATIC_FILES
frequency_file                 : T1-1a-EZ_rdkit_conf_1_HP.log
gaussian_directory             : gaussian
gaussian_max_filenames         : 10000

# Threading Options
number_of_simultaneous_trajectories      : 4

# Gaussian Options
number_of_processors_per_trajectory      : 8
memory_per_trajectory                    : 16
gaussian_force_route_card                : uwb97xd/6-31+g(d) scrf=(smd,solvent=dichloromethane) pop=none
gaussian_force_footer                    : @blank

# Trajectory Options
job_type                                 : trajectory
trajectory_type                          : reaction
number_of_total_trajectories             : 4
```

checkpoint_directory : checkpoints

checkpoint_prefix : 1a

checkpoint_interval : 5

temperature : 298.15

timestep : 1.0

number_of_forward_points : 1000

number_of_backward_points : 1

Initialization Options

maximum_number_of_initialization_attempts : 50

harmonic_tolerance : 0.0005

scale_factor : 1.0

vibrational_initialization_default : quasiclassical

vibrational_initialization_override : 0:ts_positive

rotational_initialization_type : classical

Reaction Trajectory Termination Conditions

Note: these options will be ignored if trajectory_type is set to "nmr."

termination_condition : no_termination_conditions

NMR Calculations

Note: these options will be ignored if trajectory_type is set to "reaction".

nmr_point_interval : 8

shieldings_file : @blank

gaussian_nmr_route_card : b3lyp/cc-pvdz

gaussian_nmr_footer : @blank

symmetry_groups : @blank

Analysis Options

Note: these options will only be processed if job_type is set to "analysis."

analysis_directory : analysis

make_molden_movies : yes

summarize_trajectories_to_screen : yes

summary_interval : 20

analysis_coordinate : torsion, 2, 12, 13, 14, oxal-CC

write_analysis_csv : yes

6.5.- T_1 rotations of 1a and 4

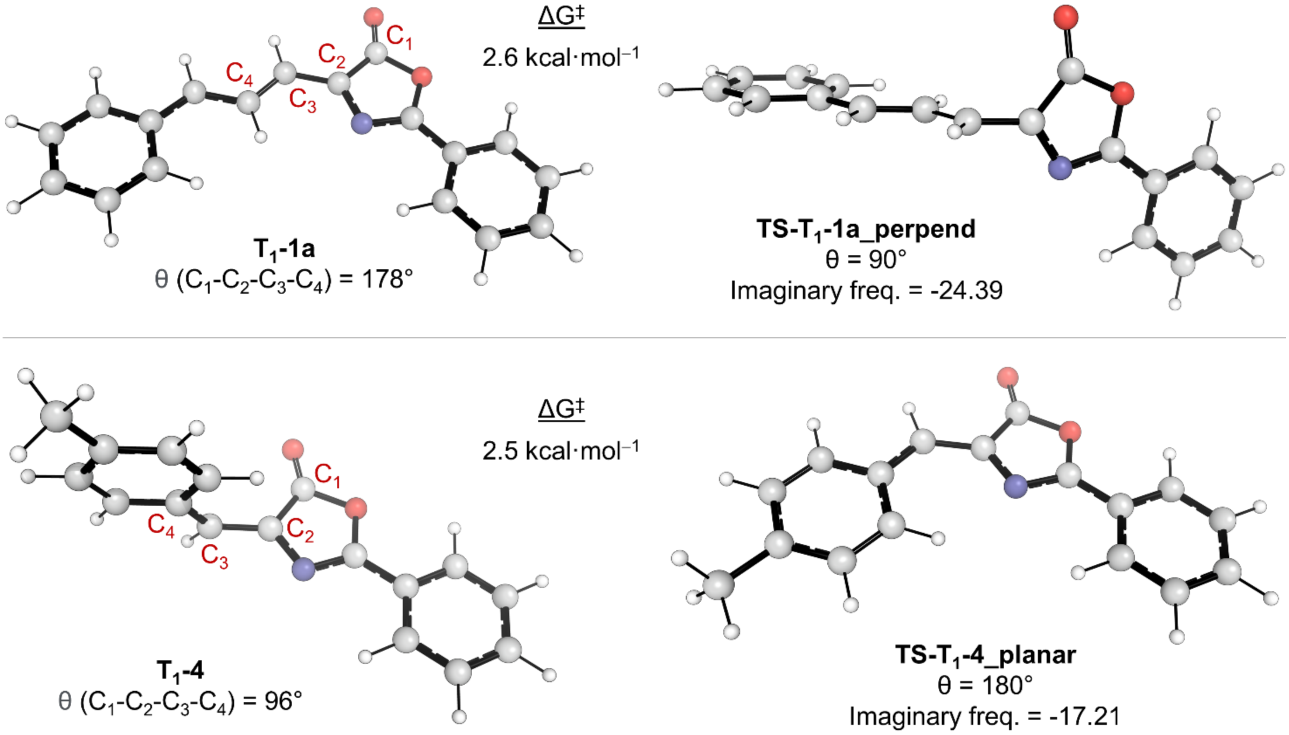

Figure S94. Stationary minimum points and transition structures found for oxazolones **1a** and **4** in T_1 state, along with the energy barrier from the two systems ($\Delta G^{\ddagger}$).

References

[1] A. D. Becke, Density-functional thermochemistry. V. Systematic optimization of exchange-correlation functionals. *J. Chem. Phys*. **107**, 8554-8560, doi: 10.1063/1.475007 (1997).

[2] J.-D. Chai, M. Head-Gordon, Long-range corrected hybrid density functionals with damped atom–atom dispersion corrections. *Phys. Chem. Chem. Phys*. **10**, 6615-6620, doi: 10.1039/B810189B (2008).

[3] Rassolov, V. A., Ratner, M. A., Pople, J. A., Redfern, P. C., Curtiss, L. A. 6-31G* basis set for third-row atoms. *J. Comput. Chem.* **22**, 976-984, doi: 10.1002/jcc.1058 (2001).

[4] Francl, M. M., Pietro, W. J., Hehre, W. J., Binkley, J. S., Gordon, M. S. DeFrees, D. J. & Pople, J. A. Self-consistent molecular-orbital methods. 23. A polarization-type basis set for second-row elements. *J. Chem. Phys.* **77**, 3654-3665, doi: 10.1063/1.444267 (1982).

[5] Hariharan, P. C. & Pople, J. A. Influence of polarization functions on molecular-orbital hydrogenation energies. *Theor. Chim. Acta* **28**, 213-222, doi: 10.1007/BF00533485 (1973).

[6] Hehre, W. J., Ditchfield, R. & Pople, J. A. Self-consistent molecular-orbital methods. 12. Further extensions of Gaussian-type basis sets for use in molecular-orbital studies of organic-molecules. *J. Chem. Phys.* **56**, 2257-2261, doi: 10.1063/1.1677527 (1972).

[7] Clark, T., Chandrasekhar, J., Spitznagel, G. W. & Schleyer, P. Von R. Efficient diffuse function-augmented basis sets for anion calculations. III. The 3-21+G basis set for first-row elements, Li–F. *J. Comput. Chem.* **4**, 294-301. doi: 10.1002/jcc.540040303 (1983).

[8] *GoodVibes*, version 3.2. Luchini, G., Alegre-Requena, J. V., Funes-Ardoiz, I. & Paton, R. S. GoodVibes: automated thermochemistry for heterogeneous computational chemistry data. *F1000Research* **9**, 291, doi: 10.12688/f1000research.22758.1 (2020).

[9] Cances, E., Mennucci, B. & Tomasi J. A new integral equation formalism for the polarizable continuum model: Theoretical background and applications to isotropic and anisotropic dielectrics. *J. Chem. Phys.* **107**, 3032-3041, doi: 10.1063/1.474659 (1997).

[10] Mennucci, B., Cances, E. & Tomasi, J. Evaluation of solvent effects in isotropic and anisotropic dielectrics and in ionic solutions with a unified integral equation method: Theoretical bases, computational implementation, and numerical applications. *J. Phys. Chem. B* **101**, 10506-10517, doi: 10.1021/jp971959k (1997).

[11] Scalmani, G. & Frisch, M. J. Continuous surface charge polarizable continuum models of solvation. I. General formalism. *J. Chem. Phys.* **132**(11), 114110, doi: 10.1063/1.3359469 (2010).

[12] Tomasi, J., Mennucci, B. & Cances, E. The IEF version of the PCM solvation method: an overview of a new method addressed to study molecular solutes at the QM ab initio level. *Journal of Molecular Structure-Theochem* **464**, 211-226, doi: 10.1016/S0166-1280(98)00553-3 (1999).

[13] Mennucci, B. & Tomasi, J. Continuum solvation models: A new approach to the problem of solute's charge distribution and cavity boundaries. *J. Chem. Phys.* **106**, 5151-5158, doi: 10.1063/1.473558 (1997).

[14] Marenich, A. V., Cramer, C. J. & Truhlar, D. G. Universal solvation model based on solute electron density and on a continuum model of the solvent defined by the bulk dielectric constant and atomic surface tensions. *J. Phys. Chem. B* **113**, 6378-6396, doi: 10.1021/jp810292n (2009).

[15] Gaussian 16, Revision C.01, Frisch, M. J., Trucks, G. W., Schlegel, H. B., Scuseria, G. E., Robb, M. A., Cheeseman, J. R., Scalmani, G., Barone, V., Petersson, G. A., Nakatsuji, H., Li, X., Caricato, M., Marenich, J., Bloino, A., Janesko, B. G., Gomperts, R., Mennucci, B., Hratchian, H. P., Ortiz, J. V., Izmaylov, A. F., Sonnenberg, J. L., Williams-Young, D., Ding, F., Lipparini, F., Egidi, F., Goings, J., Peng, B., Petrone, A., Henderson, T., Ranasinghe, D., Zakrzewski, V. G., Gao, J., Rega, N., Zheng, G., Liang, W., Hada, M., Ehara, M., Toyota, K., Fukuda, R., Hasegawa, J., Ishida, M., Nakajima, T., Honda, Y., Kitao, O., Nakai, H., Vreven, T., Throssell, K., Montgomery, Jr., J. A., Peralta, J. E., Ogliaro, F., Bearpark, M., Heyd, J. J., Brothers, E., Kudin, K. N., Staroverov, V. N., Keith, T., Kobayashi, R., Normand, J., Raghavachari, K., Rendell, A., Burant, J. C., Iyengar, S. S., Tomasi, J., Cossi, M., Millam, J. M., Klene, M., Adamo, C., Cammi, R., Ochterski, J. W., Martin, R. L., Morokuma, K., Farkas, O., Foresman, J. B. & Fox, D. J. Gaussian, Inc., Wallingford CT, 2016.

[16] https://gist.github.com/bobbypaton.

[17] *NBO*, version 7.0, Glendening, E. D., Badenhoop, J. K., Reed, A. E., Carpenter, J. E., Bohmann, J. A., Morales, C. M., Karafiloglou, P., Landis, C. R. & Weinhold, F. Theoretical Chemistry Institute, University of Wisconsin, Madison WI, 2018.

[18] Kwan, E. E. & Liu, R. Y. Enhancing NMR Prediction for Organic Compounds Using Molecular Dynamics. *J. Chem. Theory Comput.* **11***,* 5083-5089, doi: 10.1021/acs.jctc.5b00856 (2015).

[19] AQME v1.4, Alegre-Requena, J. V., Sowndarya, S., Pérez-Soto, R., Alturaifi, T. & Paton, R. AQME: Automated Quantum Mechanical Environments for Researchers and Educators. *Wiley Interdiscip. Rev. Comput. Mol. Sci.*, doi: 10.1002/wcms.1663 (2023).

[20] CREST, version 2.12. Pracht, P., Bohle, F. & Grimme, S. Automated exploration of the low-energy chemical space with fast quantum chemical methods, *Phys. Chem. Chem. Phys*. **22**, 7169-7192, doi: 10.1039/C9CP06869D (2020).

[21] cclib, version 1.7.2. O'Boyle, N. M., Tenderholt, A. L. & Langner, K. M. cclib: a library for package-independent computational chemistry algorithms. *J. Comp. Chem.* **29**, 839-845, doi: 10.1002/jcc.20823 (2008).

[22] Grimme, S. Supramolecular binding thermodynamics by dispersion-corrected density functional theory. *Chem. Eur. J.* **18**, 9955-9964, doi: 10.1002/chem.201200497 (2012).

[23] Bryantsev, V. S., Diallo, M. S. & Goddard III, W. A. Calculation of solvation free energies of charged solutes using mixed cluster/continuum models. *J. Phys. Chem. B* **112**, 9709-9719, doi: 10.1021/jp802665d (2008).

[24] Álvarez-Moreno, M., de Graaf, C., López, N., Maseras, F., Poblet, J. M. & Bo, C. Managing the Computational Chemistry Big Data Problem: The ioChem-BD Platform. *J. Chem. Inf. Model.* **55**, 1, 95-103, doi: 10.1021/ci500593j (2015).

[25] Essafi, S. & Harvey, J. N. Rates of molecular vibrational energy transfer in organic solutions. *J. Phys. Chem. A* **122**, 3535-3540, doi: 10.1021/acs.jpca.7b12563 (2018).
